# Characterization and Prediction of Haploinsufficiency Using Systems-Level Gene Properties in Yeast

**DOI:** 10.1534/g3.113.008144

**Published:** 2013-11-01

**Authors:** Matthew Norris, Simon Lovell, Daniela Delneri

**Affiliations:** Faculty of Life Sciences, University of Manchester, Manchester, Lancashire, M13 9PT, United Kingdom

**Keywords:** haploinsufficiency, prediction, machine-learning, correlation, genome

## Abstract

Variation in gene copy number can significantly affect organism fitness. When one allele is missing in a diploid, the phenotype can be compromised because of haploinsufficiency. In this work, we identified associations between *Saccharomyces cerevisiae* gene properties and genome-scale haploinsufficiency phenotypes from previous work. We compared the haploinsufficiency profiles against 23 gene properties and found that genes with higher level of connectivity (degree) in a protein–protein interaction network, higher genetic interaction degree, greater gene sequence conservation, and higher protein expression were significantly more likely to be haploinsufficient. Additionally, haploinsufficiency showed negative relationships with cell cycle regulation and promoter sequence conservation.

We exploited the association between *Saccharomyces cerevisiae* gene properties and genome-scale haploinsufficiency (HI) phenotypes using linear discriminant analysis to predict HI in existing data and to guide experimental identification of six novel haploinsufficient phenotypes, previously undetected in genome-scale screenings. Using a similar approach, we identified significant relationships between haploinsufficiency and two gene properties in *Schizosaccharomyces pombe*, relationships that hold despite the lack of conserved HI between *Saccharomyces cerevisiae* and *Sz. pombe* ortholog pairs. These data suggest associations between haploinsufficiency and gene properties are conserved among hemiascomycetes yeasts. The relationships and predictive model presented here are a useful step toward understanding haploinsufficiency and its underlying mechanisms.

A gene is considered haploinsufficient when a reduction of the gene copy number from 2 to 1 induces a growth defect. HI may be caused by a lower amount of protein being produced, such that it is insufficient to perform the biological function effectively. Moreover, if the protein is part of a larger network, then stoichiometry among different members of a protein complex can be altered. Alternatively, if a protein is an enzyme with a high flux control coefficient, the entire metabolic pathway efficiency can be disrupted.

The availability of a comprehensive *S. cerevisiae* knockout library has enabled studies of HI across the genome. A previous work determined the HI profile of almost all genes in yeast cultures grown in rich and minimal medium (Deutschbauer *et al.* 2005). The study showed that approximately 3% of yeast genes display HI, comprising 98 essential genes and 86 nonessential genes. Interestingly, the fitness distributions of essential and nonessential HI strains are approximately the same, and only 98 out of 1102 essential genes are HI in rich medium. These observations suggest that the relationship between gene essentiality and HI is not simple.

More recently, HI phenotypes have been studied in chemostat, where nutrients and pH were rigorously kept constant throughout the competition. The HI profile was analyzed in cultures grown on complex natural media, such as in grape juice extract, and in different chemical-defined media limited for carbon, nitrogen, and phosphate (Delneri *et al.* 2008). Although in grape juice a small proportion of genes were found to be HI, in the nutrient-limited medium a much larger number of HI genes were detected (10–20% of the genome).

It is, however, likely that in rich medium, some HI phenotypes were missed because of the large-scale “top down” nature of such experiments. These data are obtained by simultaneously competing 6000 different mutant strains, and therefore are likely to contain “false-negative” observations because of interactions between strains in the culture. Because all the knockout strains share the same environment during the competition, different metabolites can be excreted by the different mutants, altering the fitness of the other strains independently from the environmental limitation imposed. Studies of the yeast exometabolome have shown how complex and diverse the pattern of metabolites in the supernatant of different yeast mutants can be (Allen *et al.* 2003). To avoid strain interactions, HI profiles should ideally be recorded by comparing growth curves of monocultures, because these contain only the mutant strain being studied. One-to-one competition experiments with a reference strain may also be better than pooled competition experiments in this respect, although results from such experiments may still be affected by interactions between the reference strain and the mutant. Another issue with genome-scale studies is that the significance of HI identification is determined by levels of agreement between biological replicates, and genuine HI assignments can be disregarded if one biological replicate is of low quality.

In our study, we set out to explore cases in which genuine HI phenotypes may have been missed in earlier genome-scale experiments. We searched for these cases using a computational model that, for each gene, calculates an inferred probability of the gene being HI. The model infers HI probabilities by exploiting relationships between previously observed HI and systems-level gene properties. Our modeling approach based on gene properties is similar to a study published recently in which gene properties were used to predict genetic interaction (GI) degree in *S. cerevisiae* and *Sz. pombe* (Koch *et al.* 2012). Many gene properties correlated with GI degree in both organisms, despite the lack of GI degree conservation between *S. cerevisiae* and *Sz. pombe* ortholog pairs.

In this work, we chose a set of candidate genes that had high model-inferred HI probability but were not found to be HI in an earlier genome-scale screen. We have opted to determine HI phenotypes using monoculture experiments, because this method avoids phenotypic effects caused by strain interactions. The gene prioritization approach allowed us to find novel phenotypes without resorting to the laborious process of measuring monoculture growth curves of strains hemizygous for all known yeast genes.

To build our model, we compared HI profiles in six nutrient environments against 23 systems-level gene properties. For many of these gene properties, values differed significantly between HI and non-HI genes. We used six gene properties that associated strongly with HI but had low correlation between them to build a machine-learning model for predicting HI. Many gene properties such as protein interaction degree (Jeong *et al.* 2001; He and Zhang 2006; Zotenko *et al.* 2008) have been shown to positively correlate with gene essentiality. Because essential genes are over-represented among HI genes (Deutschbauer *et al.* 2005), we investigated the possibility that gene essentiality might be a confounding factor between HI and gene properties used in our model. Our data show that in most cases, gene essentiality does not fully explain the relationships between HI and gene properties. This finding suggests that there is an interesting difference between the mechanisms of HI and gene essentiality.

Our model used the established linear discriminant analysis (LDA) method (Hastie *et al.* 2009). Training of the model was performed using HI profiles in rich, minimal, carbon-limited, nitrogen-limited, phosphate-limited, and grape juice environments (Deutschbauer *et al.* 2005; Delneri *et al.* 2008).

We found that the models constructed for rich and minimal media were capable of predicting HI in these environments effectively. Of the mutants that were not identified as HI in the large-scale rich medium study, we selected 23 genes with the highest probabilities of HI according to our model. We tested the corresponding mutants for HI in rich medium by measuring and comparing growth curves of monocultures. Interestingly, 26% of our candidate genes showed a clear HI phenotype, validating our prediction method for use in refining HI phenotype profiles in *S. cerevisiae*.

To explore whether the relationships between gene properties and HI found in *S. cerevisiae* hold for other organisms, we examined ORF conservation and protein interaction degree in *Sz. pombe* and found weak but significant relationships with HI in rich medium. We argue that our HI prediction method could be applied to other species, because two *S. cerevisiae* associations are also found in *Sz. pombe*; these are seen even though HI profiles are not conserved between *S. cerevisiae* and *Sz. pombe* orthologs. The associations uncovered here between gene properties and HI are valuable in terms of identifying novel HI phenotypes and understanding underlying HI mechanisms.

## Materials and Methods

### Haploinsufficiency data

Haploinsufficiency data for yeast peptone dextrose (YPD) and minimal media (MM) were obtained from a previous study (Deutschbauer *et al.* 2005). Genes identified as significantly HI in the earlier study were considered HI in this work. Haploinsufficiency data for carbon-limited, nitrogen-limited, phosphate-limited, and grape juice environments were also accessed (Delneri *et al.* 2008). As before, genes identified as significantly HI were considered HI in this work. ORF names from the two data sources were standardized to the most up-to-date *Saccharomyces* Genome Database (SGD) gene identifier (Cherry *et al.* 2011). In some cases, because of gene name changes, gene identifiers from data sources mapped to either no SGD ORF or to multiple SGD ORF identifiers; such genes were excluded from the analysis. Genes identified as dubious in SGD were also disregarded.

### Gene property data

For each gene property, standardization of gene names from the appropriate data source was performed as described. Genes that did not appear in either of the two genome-scale HI studies were excluded. Gene essentiality data for YPD medium were obtained from a previous study (Giaever *et al.* 2002).

The Perl Graph::Undirected library was used to calculate genetic interaction degree and betweenness centrality (Freeman 1977) from the stringent, intermediate, and lenient cut-off datasets available in the Drygin database (Koh *et al.* 2009). Scores for mRNA expression variation through the yeast cell cycle were obtained from a previous study (Spellman *et al.* 1998). Summed intensities describing combined haploid and diploid abundance of protein were obtained from a previous proteomics study (de Godoy *et al.* 2008).

Interaction network data were downloaded from the BioGRID database (Stark 2006) on 26 January 2012. Two filtered datasets were produced alongside an unfiltered dataset; these excluded reactions reported less than either twice or three times in the literature. Both the filtered and the unfiltered datasets only included interactions reported as physical, thereby excluding genetic interactions. Degree and betweenness (Freeman 1977) for physical interactions were then calculated using the Perl Graph::Undirected library.

*S. paradoxus* strain CBS432 sequence data were retrieved from the *Saccharomyces* Genome Resequencing project website (Liti *et al.* 2009). Sequences of the *S. kudriavzevii* strain IFO 1802 and the *S. bayanus* strain MCYC623 were obtained from two studies (Scannell *et al.* 2011; Kellis *et al.* 2003). The *S. cerevisiae* sequence was the SGD reference strain, S288C (Cherry *et al.* 2011). Alignments were performed using the BioPerl library; for each gene, cDNAs from *S. cerevisiae* and a selected clade member were first translated into protein, followed by alignment using dynamic programming (with the BioPerl dpAlign function, default parameters), and then projection of protein sequence alignments back to DNA. DNA and protein percentage identity, *dN*, *dS*, and *dN/dS* (Hurst 2002) were then calculated.

Because of the high rate of evolution for promoter regions, sequence comparisons were performed between *S. cerevisiae* and *S. paradoxus*, the most closely related *sensu stricto* (Scannell *et al.* 2011) species according to current knowledge. Promoter sequence was defined as the region spanning 500 bp upstream of the ORF start codon, but trimmed such that it did not overlap adjacent ORFs. Although consideration of transcription start sites (TSS) would be advantageous, these were not considered as gene boundaries because of unavailable TSS annotations for *S. paradoxus*. Hence, promoter regions used in this work were inclusive approximations based on the current knowledge of ORF boundaries. Sequences were aligned using dynamic programming with the BioPerl dpAlign method (default parameters) and percentage identity was calculated.

After standardization of gene names as described, gene property data from the sources described were combined into a single table with genes as rows and properties as columns, allowing data to be easily manipulated. The resultant table, used in statistics tests and LDA model generation, is provided in Supporting Information, File S1.

### Statistical tests

To determine whether gene property value distributions were significantly different between HI and non-HI genes, the Mann-Whitney *U*-test (Mann and Whitney 1947) was performed using the wilcox.test() function from the R “stats” package to produce estimated *p*-values. To provide information about the difference between the means of HI and non-HI gene properties, *z*-scores were also calculated. To assess the predictive ability of single gene properties, receiver-operating characteristic (ROC) plot areas under curves (AUCs) were calculated using the R “ROCR” package and 20 repeats of five-fold cross-validation were performed as described. Pairwise relationships between gene properties were assessed by determining Pearson product-moment coefficient (Pearson 1896) using the R statistics cor.test() function.

In many cases described in this text, the variance-stabilizing transformation log10(*x* + 0.5) was applied to gene properties with values that followed a log-normal distribution. Properties transformed in this manner include protein–protein interaction (PPI) degree and betweenness, genetic interaction (GI) degree, mRNA expression variation, and protein expression levels.

### HI prediction in *S. cerevisiae*

LDA was performed using the lda() function from the R package “MASS.” This method assumes data are normally distributed. Many gene properties had values following a log-normal distribution, so variance-stabilizing transformations were applied as described. Overfitting was avoided by performing five-fold cross validation, with 20 repeats to overcome variability. Five-fold cross-validation was chosen to ensure a reasonably large sample size in the left-out set.

Several methods were attempted to deal with incomplete cases in which the properties of genes had missing values. The simplest method removed genes with missing data. Another method involved drawing the posterior probability of a gene’s haploinsufficiency from the most complete simpler model when missing values were encountered. In other words, if a gene had four properties, A, B, C, and D, and B was missing, then the posterior probability of HI was taken from the model that considered the properties A, C, and D.

Imputation methods to estimate missing values were also examined. Expectation maximization (EM) (Do and Batzoglou 2008) and multiple imputation (MI) (Kenward and Carpenter 2007) were performed using functions from the R “mix” package. MI and EM were performed separately for each cross-validation fold. EM and MI methods were used with selected gene properties along with observed haploinsufficiency as a Boolean categorical variable. MI was also used as an alternative method. For each gene property, missing values were set to the median of that property within either the HI or the non-HI category based on the gene’s observed haploinsufficiency. When cross-validation and missing value imputation were performed together, the left-out fold was hidden from the imputation process.

After producing LDA models, the R predict() function was used to generate posterior probabilities, describing the likelihood of particular genes being either HI or non-HI. To assess level of agreement between predicted HI likelihoods and HI observations, ROC curves were produced using the R “ROCR” package. False-positive rate (FPR) and true-positive rate (TPR) were calculated by sweeping a posterior probability cut-off. In cases in which cross-validation was performed, 5 × 20 ROC curves were produced.

### Analysis of HI in *Sz. pombe*

PPI data were downloaded from the BioGRID website (Stark 2006), and predicted GI degrees were obtained from a previous study (Koch *et al.* 2012). cDNA sequence data for *Sz. pombe*, *Sz. octosporus*, and *Sz. cryophilus* (Rhind *et al.* 2011) were downloaded from the Broad Institute website (http://www.broadinstitute.org/). Orthologous cDNAs were then aligned using the same algorithm as described for *S. cerevisiae*. DNA and protein percentage identity, *dN*, *dS*, and *dN*/*dS*, for *Sz. pombe* against *Sz. octosporus* and *Sz. pombe* against *Sz. cryophilus* were then calculated and considered as gene properties. These gene properties were compared against HI data from a previous work (Kim *et al.* 2010). Genes with fitness relative to wild-type (WT) less than 0.95 and *p*-value < 0.05 were considered significantly HI. Relationships between sequence conservation statistics and HI were examined through the Mann-Whitney *U*-test, calculation of *z*-scores, and drawing of ROC curves as described for *S. cerevisiae*. We used orthology data from PomBase (Wood *et al.* 2012) to determine the level of rich medium HI phenotype overlap between *S. cerevisiae* and *Sz. pombe* orthologs.

### Strains and growth media

The *S. cerevisiae* BY4743 WT strain and hemizygous mutants for genes of interest were obtained from a genome-wide deletion collection (EUROSCARF http://web.uni-frankfurt.de/fb15/mikro/euroscarf/index.html).

YPD medium was prepared using 2% (w/v) peptone, 1% (w/v) yeast extract, and 2% (w/v) glucose. YPDA was made as YPD, but with the addition of 1% (w/v) agar. Synthetic-defined F1 medium was prepared as described previously (Delneri 2011) with the following per liter: 62 mg inositol; 14 mg thiamine HCl; 4 mg pyridoxine; 4 mg calcium panthothenate; 0.3 mg biotin; 70 µg ZnSO_4_(H_2_O)_7_; 10 µg CuSO_4_(H_2_O)_5_; 10 µg H_3_BO_3_; 10 µg KI; 50 µg FeCl_3_(H_2_O)_6_; 3.13 g (NH_4_)_2_SO_4_; 2 g KH_2_PO_4_; 0.55 g MgSO_4_(H_2_O)_7_; 0.1 g NaCl; 90 mg CaCl_2_(H_2_O)_2_; and 2% w/v glucose. To produce F1 medium with carbon limitation, the F1 medium mixture was modified by changing the glucose concentration to 0.25% (w/v). F1 medium with nitrogen limitation was produced as the synthetic-defined F1 medium, but with (NH_4_)_2_SO_4_ concentration changed to 0.46 g/liter. All F1 medium preparations were supplemented with the following per liter: 20 mg histidine; 100 mg leucine; 30 mg lysine; 20 mg methionine; and 20 mg uracil.

### Experimental determination of HI in *S. cerevisiae*

To assay for HI, strains were plated onto YPDA medium. After 48 hr, strains were inoculated into overnight cultures, comprising either 5 ml YPD for obtaining growth curves in YPD medium or 5 ml synthetic-defined F1 medium for testing in F1 media with carbon or nitrogen limitation. These cultures were left in a shaker-incubator at 30° for 20 hr. The next day, cultures were diluted to a final OD_595_ of 0.1 in either YPD, nitrogen-limited media, or carbon-limited media as appropriate. The diluted cultures were then distributed in a 96-well plate (240 µL per well) with six replicates per strain. The outermost wells were filled only with sterile medium to test for cross-contamination of culture between wells. The positions of all the biological replicates of the mutant strains were randomized on the plate to minimize bias. Growth curves were obtained by measuring absorbance at 595 nM in 5-min intervals for 24 hr with shaking at each interval using a BMG Labtech FLUOstar OPTIMA plate reader. The average absorbance of the wells containing sterile medium was subtracted from absorbance of each well containing culture. The lag phase, growth rate, and biomass of the growth curves were then compared between mutant and WT strains.

To analyze the data and determine growth defects, we calculated the area under the growth curve (AUGC) across each strain and replicate. AUGCs were calculated for the first 15 hr of growth in YPD, the first 25 hr of growth in nitrogen-limited medium, and the first 23 hr of growth in carbon-limited medium. These time intervals were chosen to capture the region of the growth curve before the stationary phase. Using Welch *t*-test, we calculated *p*-values for the significance of the difference between the mutant and WT AUGC sets. The set of *p*-values for all mutants *vs.* WT was then corrected using the Benjamini–Hochberg procedure (Benjamini and Hochberg 1995) to control for false discoveries.

## Results

### Strategy to assess relationships between gene properties and HI in *S. cerevisiae*

**Figure 1:**
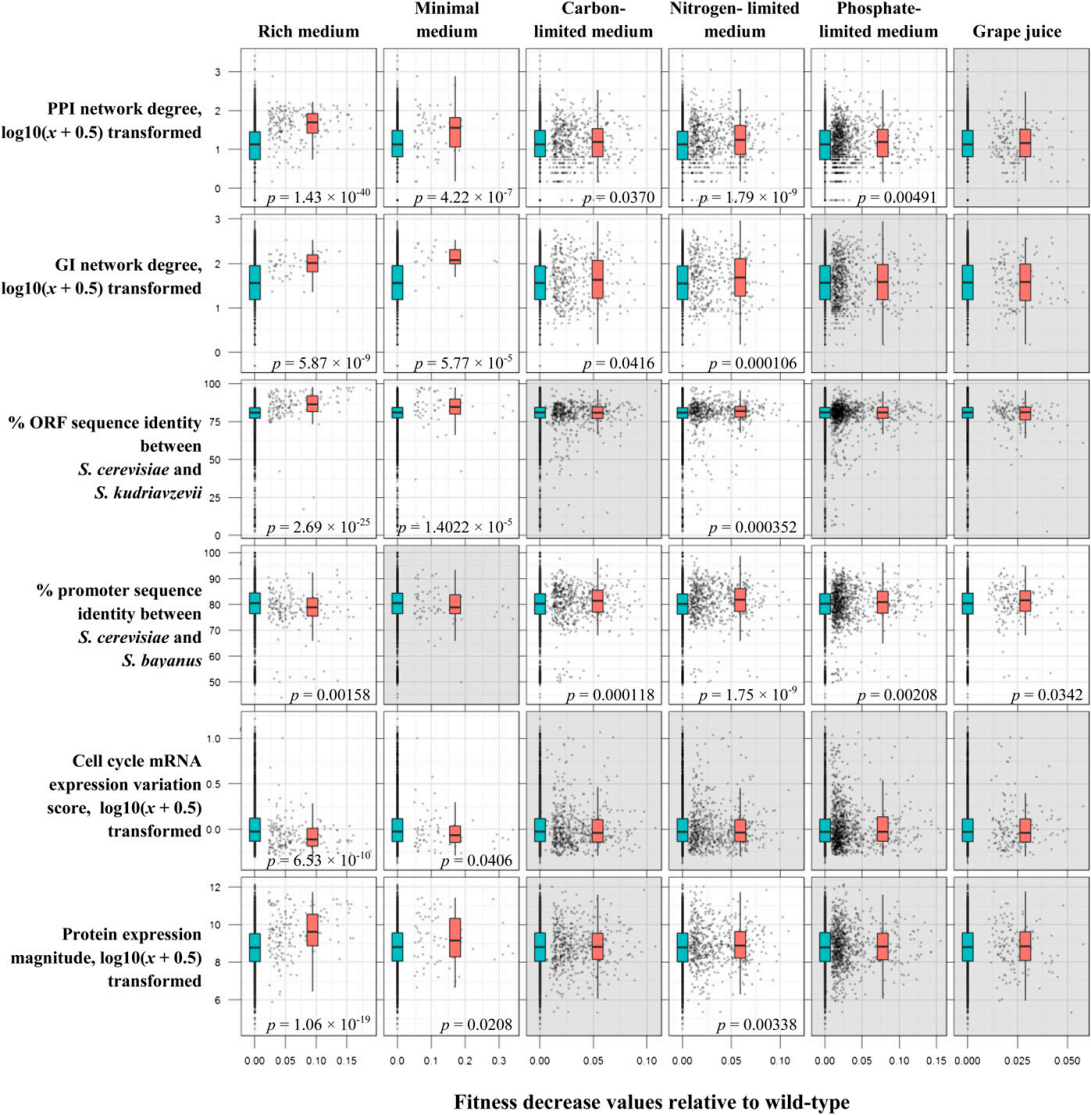
Distributions of gene property values with respect to HI phenotypes in six different media. To find relationships between HI and gene properties, the gene property data were divided into HI and non-HI groups. The significance of the difference between HI and non-HI property values was then estimated through the Mann-Whitney *U*-test (significant differences are indicated with white panels and *p*-values). The X axis indicates fitness loss values relative to wild-type (WT) in six nutrient environments, whereas the Y axis describes gene property values. We have visualized the raw HI fitness values using a scatter plot, with each dot representing an individual gene. The overlaid box plots represent non-HI (blue) and HI (red) gene property distributions. The box represents the upper and lower quartiles, and the central line represents the median. Whiskers represent the lowest point within the 1.5 interquartile range (IQR) of the lower quartile and represent the highest point within 1.5 IQR of the upper quartile.

We investigated associations between 23 gene properties (Table 1) and HI detected in six different nutrient environments. After comparing HI and non-HI gene property value distributions to identify gene properties significantly associated with HI, we chose six gene properties that were suitable for building a machine-learning model. Comparisons between these properties and HI across six nutrient environments are shown in Figure 1.

**Table 1:** List of the 23 gene properties that were considered for LDA model building

Gene Property	Description
Protein–protein interaction degree, ≥1× reported	Generated by calculating degree and betweenness for each gene according to physical interactions in the BioGRID (Stark 2006) database after filtering to remove interactions reported less than once, twice, or three times
Protein–protein interaction degree, ≥2× reported	
Protein–protein interaction degree, ≥3× reported	
Protein–protein interaction betweenness, ≥1× reported	
Protein–protein interaction betweenness, ≥2× reported	
Protein–protein interaction betweenness, ≥3× reported	
Genetic interaction degree, lenient cut-off	Generated from genetic interaction data in the DRYGIN (Koh *et al.* 2009) database. There are three data sets provided by the aforementioned work, generated from “lenient,” “intermediate,” and “stringent” cut-offs. These data sets were tested for predictive ability separately.
Genetic interaction degree, intermediate cut-off	
Genetic interaction degree, stringent cut-off	
Genetic interaction betweenness, intermediate cut-off	
Genetic interaction betweenness, stringent cut-off	
ORF protein sequence identity Sc ↔ Sp	Calculated by examining protein sequence conservation between *S. cerevisiae* and *S. paradoxus* for each gene
ORF DNA sequence identity Sc ↔ Sp	Gene DNA sequence identity between *S. cerevisiae* and *S. paradoxus*
ORF DNA dN/dS Sc ↔ Sp	dN/dS calculated by comparing ORF sequence between *S. cerevisiae* and *S. paradoxus*
ORF protein sequence identity Sc ↔ Sk	Calculated by examining protein sequence conservation between *S. cerevisiae* and *S. kudriavzevii* for each gene
ORF DNA sequence identity Sc ↔ Sk	Gene DNA sequence identity between *S. cerevisiae* and *S. kudriavzevii*
ORF DNA dN/dS Sc ↔ Sk	dN/dS calculated by comparing ORF sequence between *S. cerevisiae* and *S. kudriavzevii*
ORF protein sequence identity Sc ↔ Sb	Calculated by examining protein sequence conservation between *S. cerevisiae* and *S. bayanus* for each gene
ORF DNA sequence identity Sc ↔ Sb	Gene DNA sequence identity between *S. cerevisiae* and *S. bayanus*
ORF DNA dN/dS Sc ↔ Sb	dN/dS calculated by comparing ORF sequence between *S. cerevisiae* and *S. bayanus*
Promoter DNA sequence identity Sc ↔ Sb	Calculated by comparing the noncoding region upstream of the ORF between *S. cerevisiae* and *S. bayanus*.
Cell-cycle mRNA expression variation	mRNA expression variation scores were obtained from a previous study (Spellman *et al.* 1998)
Proteomics summed intensity	This value represents the level of protein expression as the combined sum of haploid and diploid protein abundance from a previous study (de Godoy *et al.* 2008)

The first column gives the gene property name, and the second column describes the source of the gene property data.

**Figure 2:**
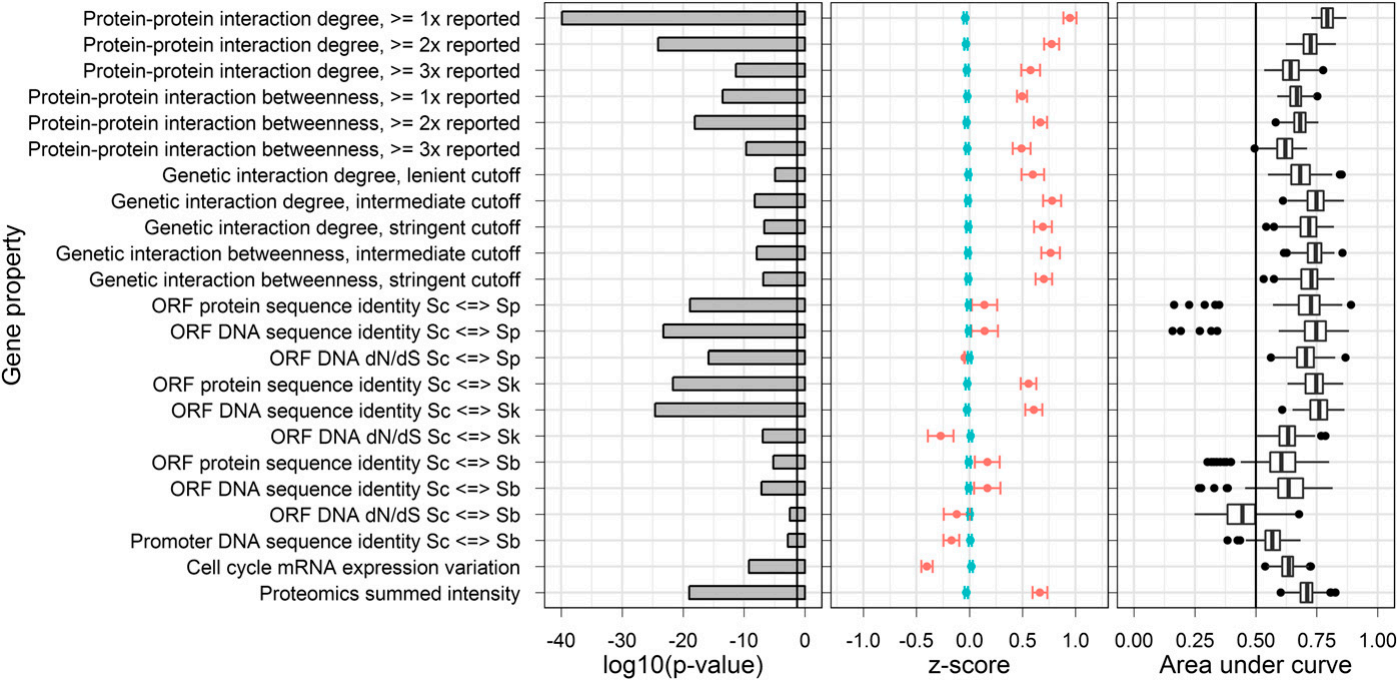
Relationships between HI and non-HI gene properties in rich medium. (A) The *p*-values testing the difference between HI and non-HI gene property value distributions. These are on a log10 scale and are as estimated by the Mann-Whitney *U*-test. The vertical line shows a *p*-value of 0.05. (B) Mean *z*-scores of HI (red) and non-HI (blue) gene properties. Error bars represent the SEM. (C) The receiver-operating characteristic (ROC) area under curve (AUC) distributions. These were generated using cross-validation (see Materials and Methods). Whiskers represent the lowest point within 1.5 interquartile range (IQR) of the lower quartile and the highest point within 1.5 IQR of the upper quartile. Dots represent outliers of the aforementioned ranges. The vertical line in the center of the chart represents the random expectation for the ROC plot.

To select particular gene properties suitable for building a model, we performed a number of analyses. For each of the 23 candidate gene properties, the difference between the HI and non-HI gene property value means was examined by classifying each mutant as either HI or non-HI in each environment. For each analysis subsequently performed, genes were removed when they had missing gene property values. The *z*-scores were calculated, which describe the number of SDs the HI and non-HI gene property means are below or above the population mean. A positive *z*-score for the HI group indicates that HI genes tend to have greater gene property values, with a negative *z*-score indicating tendency of HI genes to have lower gene property values. Estimated *p*-values for the significance of the difference between HI and non-HI gene property distributions were obtained through the Mann-Whitney *U*-test. The *p*-values and *z*-scores for rich medium are summarized in Figure 2A and Figure 2B. The *p*-values and *z*-scores for all six nutrient environments examined are reported in Figure S1.

Our data generally show that gene properties associate best with HI scored in rich medium (Figure 1). Relationships in minimal medium are moderate, and associations with HI in the limited nutrient environments including grape juice are much weaker. However, HI measured in nitrogen-limited medium interestingly associates significantly with several gene properties, and many of these relationships are not present in the other two limited media.

The observation that gene property values associate with rich medium HI more than the other media HI could be a consequence of most gene property values being obtained from rich medium experiments, making them more relevant to that environment. The strong relationships in rich medium might also be a consequence of the yeast laboratory strain being better adapted to rich medium. Therefore, we mainly focused on rich medium phenotypes and their relationships with the gene properties.

In rich medium, protein interaction network degree associates strongest with HI. Other gene properties that show strong relationships with HI in this medium include GI network degree, ORF sequence identity, and protein expression magnitude. We observed weaker associations with HI for promoter sequence conservation and mRNA expression through the cell cycle. Interestingly, although promoter sequence conservation showed a weak relationship with HI, we found significant relationships in five out of six environments, although this result should be interpreted with caution, because there is a strong link between promoter conservation and gene essentiality.

To check the predictive power of the 23 gene properties, we performed ROC curve analysis on each gene property and nutrient environment combination. We generated 100 ROC curves through 20 repeats of five-fold cross-validation. For each of the 100 ROC curves, we calculated the AUC. The distributions of these AUC data show that predictive abilities of gene properties are strongest for rich medium HI. Figure 2C shows gene property prediction effectiveness in rich medium, and Figure S1 shows details of this analysis in all six environments.

We focused on HI prediction in rich medium because the gene properties associate strongest with HI in this environment. To determine gene properties suitable for machine-learning model construction, we selected properties with an AUC higher than 0.5, because this represents the neutral expectation under a random model. Some properties could be expressed in several ways, an example being GI degree, in which calculations came from “lenient,” “intermediate,” and “stringent” cut-off datasets (see Materials and Methods for full details of gene property variants). When variants of gene properties existed, the variant with the best predictive ability was carried forward and the others were discarded.

Using the method described, we selected eight gene properties as candidates for building a prediction model. These properties included protein expression level, mRNA cell-cycle regulation score, promoter sequence identity between *S. cerevisiae* and *S. paradoxus*, cDNA sequence identity between *S. cerevisiae* and *S. kudriavzevii*, degree and betweenness in a genetic interaction network, PPI network degree calculated using the unfiltered BioGRID interaction network, and PPI network betweenness calculated using the BioGRID network previously filtered to remove interactions reported less than twice.

### Relationships between the gene properties

To build a machine-learning model, we sought sets of gene properties that each contributed unique information. To provide initial clues about the uniqueness of gene properties, we correlated each gene property pair using the Pearson method. These correlations were completed after performing variance-stabilizing transformations when appropriate (see Materials and Methods). Most gene property pairs showed weak correlation (Figure S2), and these properties were considered later for building the machine-learning model. PPI and GI network betweenness were excluded from our model, because we observed a very strong correlation between GI degree and betweenness and a similar strong correlation between PPI degree and betweenness. In both cases, betweenness was discarded because it yielded the lower AUC (Figure 2C). The correlation between degree and betweenness for both the GI and PPI networks suggests that genes and proteins with large numbers of interactions are considerably more likely to be central in their networks.

To demonstrate the uniqueness of gene properties, we produced models with different gene properties left out. We found that models incorporating all chosen gene properties (*i.e.*, those with low correlations between them) performed best, demonstrating that each gene property contributes unique information to the model.

### Associations between gene essentiality, HI, and gene properties

The rich medium study performed previously, on which most of our analysis is based (Deutschbauer *et al.* 2005), showed that HI genes have a tendency to be essential. We sought to determine whether gene essentiality exhibits a confounding effect between HI and our chosen gene properties, because some properties, such as PPI degree (Jeong *et al.* 2001; He and Zhang 2006; Zotenko *et al.* 2008), have been shown to correlate with gene essentiality.

**Figure 3:**
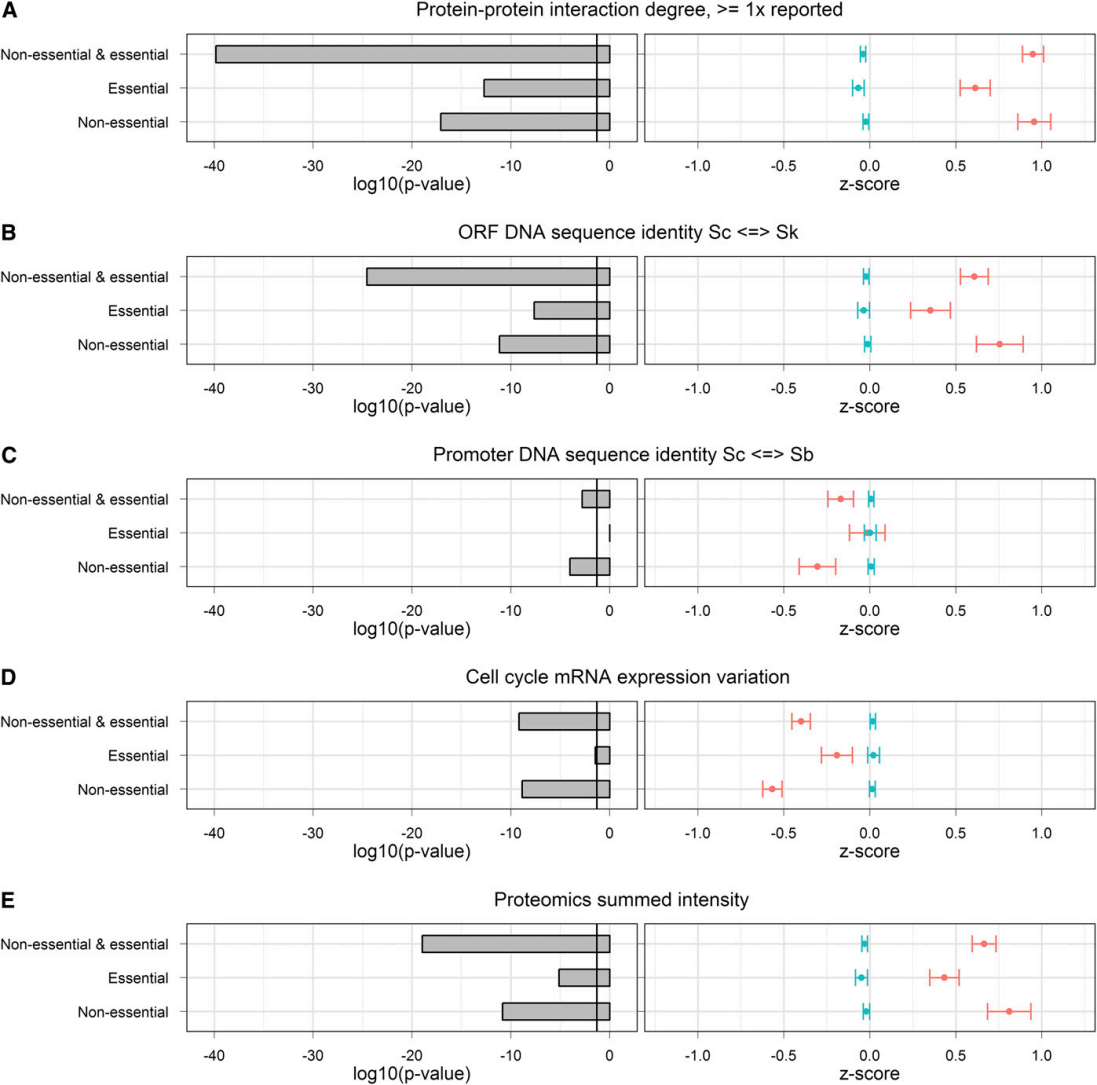
Relationships between five gene properties and HI, stratified according to gene essentiality. The *p*-values and *z*-scores represent the differences between the distributions of HI and non-HI gene property values among nonessential and essential, essential, and nonessential gene sets. Error bars represent the SEM. Gene properties include (A) PPI network degree, (B) ORF sequence identity between *S. cerevisiae* and *S. kudriavzevii*, (C) promoter sequence identity between *S. cerevisiae* and *S. bayanus*, (D) mRNA expression variation through the cell cycle, and (E) protein expression level.

We investigated this by stratifying the data according to essentiality in rich media. Within each of the two resulting strata, corresponding to either essential or nonessential genes, we analyzed the relationships between HI and each gene property. The results of this analysis for five out of six gene properties are shown in Figure 3. Genetic interaction degree is missing from the analysis because the data we used for this gene property (Costanzo *et al.* 2010) only include interactions between homozygous-null mutants and thus do not contain any information about essential genes.

Among nonessential genes, all five gene properties associate significantly with HI. For essential genes, four gene properties associate significantly with HI, with the remaining relationship involving promoter sequence identity being insignificant. Because HI associates with PPI degree, gene sequence conservation, cell-cycle expression variation, and protein abundance among both essential and nonessential genes, we can infer that although a confounding effect exists between gene essentiality and HI (Figure 3), gene essentiality does not fully explain the relationships between the gene properties and HI. Although the association between promoter sequence identity and HI among essential genes was insignificant, suggesting a strong confounding effect, we included this property in our model because it improves predictive performance.

### Construction of LDA model for HI prediction in *S. cerevisiae*

Using the selection methods, we ultimately chose six unique gene properties that we inferred would be effective at building our HI prediction model. These properties were protein abundance, mRNA expression variation through the cell cycle, DNA percentage identity of the promoter between *S. cerevisiae* and *S. paradoxus*, ORF DNA percentage identity between *S. cerevisiae* and *S. kudriavzevii*, GI degree from DRYGIN, and PPI degree from BioGRID calculated using the unfiltered network.

The model with the best combination of gene properties was established by producing an LDA model for each possible gene property combination. Every model thus produced was validated through 20 repeats of five-fold cross-validation (see Materials and Methods), yielding 100 ROC curves.

All gene properties included in our model have missing data for some genes (Table 2). To handle missing data, we tested five methods, including rolling back to a simpler model, excluding incomplete cases, EM algorithm, MI, and median imputation (see Materials and Methods). We examined model performance by calculating the AUC in the region of the ROC plot where false-positive rate is no larger than 0.1 (FPR ≤ 0.1 AUC). A high FPR ≤ 0.1 AUC minimizes the FPR and indicates a model in which genes with the highest posterior probabilities are most likely to be HI. High FPR ≤ 0.1 AUC models therefore allow genes to be ranked by their posterior probabilities such that genes at the top of the list are highly enriched for HI.

**Table 2:** List of the 6 gene properties used in the 6GP model showing proportion of gene property data missing across the yeast genome

Gene Property	Proportion of Genes with No Data (%)
PPI network degree	2.02
GI network degree	34.31
% ORF sequence identity	10.48
% Promoter sequence identity	5.00
Cell-cycle mRNA expression variation	0.50
Protein expression magnitude	17.65

PPI, protein–protein interaction; GI, genetic interaction.

**Figure 4:**
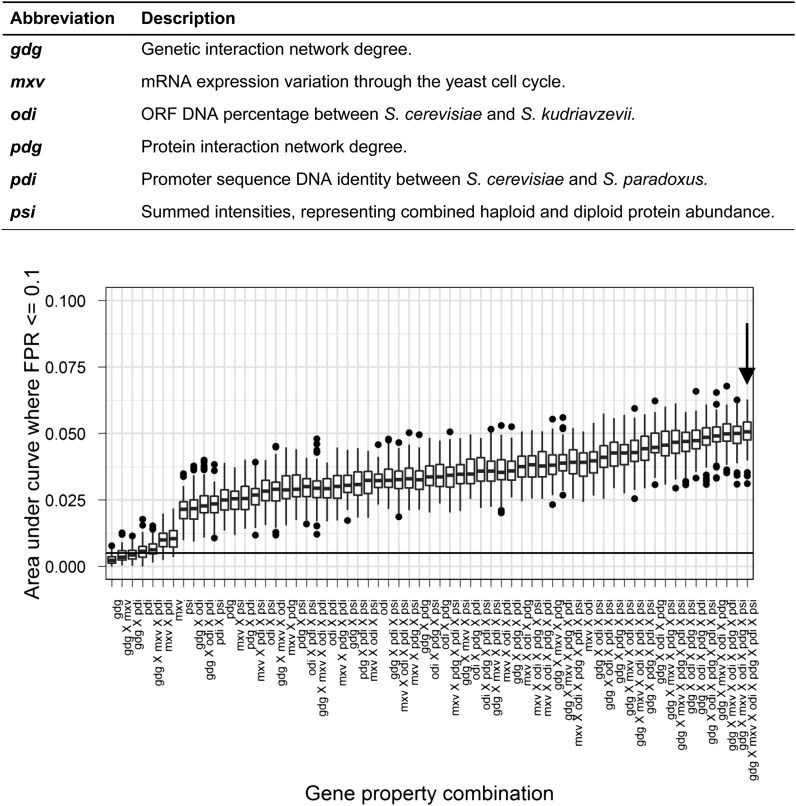
False-positive rate (FPR) ≤ 0.1 area under curve (AUC) distributions across all combinations of gene properties, using median imputation. This demonstrates that model performance tends to increase as more gene properties are added. Our candidate six gene properties (6GP) model is highlighted with an arrow. The three letter codes identify gene properties and are described in the legend. Distributions are for 100 receiver-operating characteristic (ROC) curves generated during cross-validation (see Materials and Methods). Whiskers represent the lowest point within 1.5 interquartile range (IQR) of the lower quartile and the highest point within 1.5 IQR of the upper quartile. Dots represent outliers of the aforementioned ranges. The black horizontal line represents the random expectation from the ROC plot.

The missing value handling methods produced similar ROC curves, showing that prediction quality is largely independent of the imputation method used. We chose median imputation to produce our candidate models because it yields a high FPR ≤ 0.1 AUC and has low cross-validation AUC variation. The FPR ≤ 0.1 AUC distributions for our median imputation models incorporating all possible combinations of gene properties are presented in Figure 4. These distributions are shown for all imputation methods tested in Figure S3.

**Figure 5:**
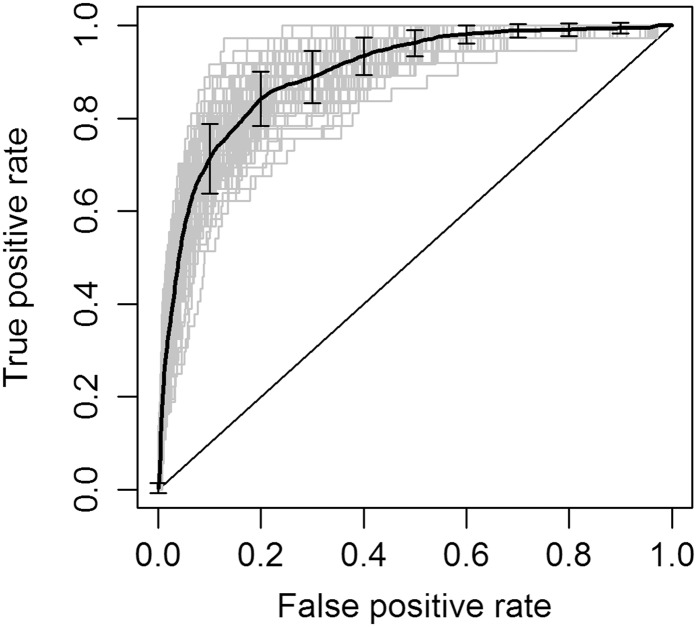
Performance of the six gene property (6GP) candidate model. Receiver-operating characteristic (ROC) curve of the best model (6GP), which combines the six gene properties described in the text. The dark line shows the average of 100 ROC curves, with error bars indicating 1 SD. Gray lines represent 100 ROC curves produced during cross-validation superimposed.

We observed a positive correlation between number of gene properties in the model and the FPR ≤ 0.1 AUC, indicating that each gene property contributes useful information for predicting HI. The model incorporating all six gene properties (6GP) was chosen as our candidate for prediction because it has the highest value for FPR ≤ 0.1 AUC. The ROC curve for the 6GP model is shown in Figure 5. We used this model to produce posterior probabilities of HI for all yeast genes, which are listed in File S2.

### Prediction of HI and experimental verification of HI in *S. cerevisiae*

We used the LDA 6GP model to rank each gene by its inferred probability of being HI. To test whether the model could infer novel and previously undetected HI phenotypes, we selected 23 candidate genes that had not been identified as HI in the previous large-scale study (Deutschbauer *et al.* 2005) but had the highest probability scores according to our model. Assays for HI were performed for all candidate genes. We found fitness defects for 6 out of 23 candidates in rich medium, identified 5 out of 23 candidates as HI in F1 medium with nitrogen limitation, and found HI in 1 out of 23 candidate genes for carbon-limited medium.

To identify significantly HI strains, we calculated an AUGC for each replicate well containing either mutant or WT culture. The *p*-value, describing the difference between mutant and WT AUGC distributions, was then calculated using the Welch *t*-test. We have reported the ratio between WT and mutant AUGC means as a quantitative measure of fitness for each candidate gene in Table 3 together with *p*-values for these differences as calculated using the Welch *t*-test and corrected using the Benjamini-Hochberg procedure.

**Table 3:** Summary of phenotypes for the 23 candidate genes tested

	AUGC Mutant/AUGC WT (*p*-value)		
Gene	Rich Media	F1 Nitrogen-Limited	F1 Carbon-Limited
*BEM2*	1.011 (0.315)	0.987 (0.462)	0.972 (0.544)
*ASC1*	1.017 (0.627)	1.008 (0.445)	0.972 (0.242)
*RPL16A*	1.007 (0.590)	0.999 (0.962)	1.002 (0.996)
*RPL8B*	0.999 (0.952)	0.995 (0.445)	1.007 (0.976)
*UBI4*	0.967 (5.33 × 10^−2^)	0.996 (0.682)	1.015 (0.841)
*RPL8A*	1.009 (0.698)	1.008 (0.431)	1.057 (6.82 × 10^−2^)
*UBP3*	1.003 (0.899)	1.009 (0.642)	1.003 (0.976)
*EFT2*	1.004 (0.794)	1.019 (0.104)	1.027 (0.544)
*RPS6B*	0.908 (8.31 × 10^−4^)*	0.965 (1.35 × 10^−4^)*	1.000 (0.996)
*BCY1*	0.903 (3.87 × 10^−5^)*	0.925 (5.39 × 10^−5^)*	0.874 (3.53 × 10^−4^)
*BRE5*	0.947 (1.48 × 10^−2^)*	0.956 (1.00 × 10^−3^)*	0.963 (0.107)
*RPS19B*	0.998 (0.899)	0.991 (0.404)	0.959 (0.159)
*RPL3*	0.996 (0.821)	0.973 (0.158)	0.978 (0.611)
*ADH1*	0.998 (0.922)	0.993 (0.445)	0.998 (0.976)
*GCN5*	0.966 (3.47 × 10^−2^)*	0.982 (0.445)	0.982 (0.752)
*ERG6*	0.990 (0.627)	1.003 (0.720)	1.016 (0.840)
*SEC28*	0.994 (0.698)	0.948 (3.33 × 10^−4^)*	0.990 (0.824)
*RPL28*	0.994 (0.718)	1.001 (0.992)	1.000 (0.996)
*HOM6*	1.035 (5.10 × 10^−2^)	1.003 (0.791)	0.995 (0.958)
*HMO1*	0.994 (0.846)	0.970 (3.47 × 10^−2^)*	0.991 (0.841)
*PHO23*	1.036 (0.118)	0.986 (0.158)	0.989 (0.824)
*RPL10*	0.942 (1.48 × 10^−2^)*	0.986 (0.104)	0.990 (0.841)
*RPN10*	0.961 (1.48 × 10^−2^)*	0.994 (0.431)	1.002 (0.996)

The first column shows the gene names and the remaining columns describe phenotypes in rich medium (YPD), F1 medium with nitrogen limitation, and F1 medium with carbon limitation. Phenotypes are described according to the average area under growth curve (AUGC) relative to the average wild-type (WT) AUGC. The number in brackets is a *p*-value representing the significance of the difference between mutant and WT AUGCs, calculated as described in Materials and Methods.

* Significantly HI phenotypes, *i.e.*, those with *p*-value < 0.05.

In addition to the 23 candidates, we selected three positive control genes that were detected as significantly HI in the earlier rich medium study. These included two genes, *TUB1* and *RPL25*, displaying severe fitness loss in heterozygosis (relative fitness losses of 0.921 and 0.818, respectively), and *RPN11*, displaying a weak but significant HI phenotype (with a relative fitness loss of 0.971). As negative controls, we selected 24 genes that had both low HI likelihood according to the model and no significant HI phenotype in the earlier experimental work. As background controls, we randomly selected 42 genes from the entire genome to produce an estimate of the rate of HI in the genome, as measured via rich medium monocultures. All controls were tested for HI using the same method used with the candidate genes.

Only 1 out of 42 background control genes showed a significant HI phenotype, in line with the expected frequency of HI as described in previous studies (Table S2). Growth curves for all significantly HI strains, corresponding to both candidate genes and controls, are shown in Figure S4

Our candidate strains each carried mutations in genes that were previously undetected in the large-scale rich medium HI profiling study (Deutschbauer *et al.* 2005). This result confirms that our proposed method for choosing HI candidates has a very good predictive power, because 26% of the candidate genes from the model were found to be HI compared to a total of 3% HI genes detected in the earlier rich medium experiment and 2.3% HI gene coverage among our randomly selected background controls. All of our negative control genes showed no significant growth rate difference from the WT BY4743 strain, demonstrating that our new phenotype identifications are unlikely to be attributable to experimental sample variation.

The HI-positive controls, *RPL25* and *TUB1*, showed pronounced and significant haploinsufficiency. *RPN11*, used as a weak haploinsufficiency-positive control, appeared to grow slightly slower than the WT strain, although the difference was not significant. These data suggest that the experimental method used may miss some weakly HI genes; therefore, the extent of the verification of the HI from our model is probably conservative.

We tested the 23 candidate genes in F1 media with nitrogen and carbon limitation, in addition to our rich medium studies, to see whether the new HI phenotypes are displayed in other environments (Table 3). The gene *BCY1* showed a HI phenotype in all three environments tested, and the genes *RPS6B*, *BCY1*, and *BRE5* showed HI phenotypes in both rich medium and the synthetically defined media with nitrogen limitation. Interestingly, in F1 medium with nitrogen limitation, we found another two candidate genes to be HI, *SEC28* and *HMO1*.

### Analysis of HI in *Sz. pombe*

**Figure 6:**
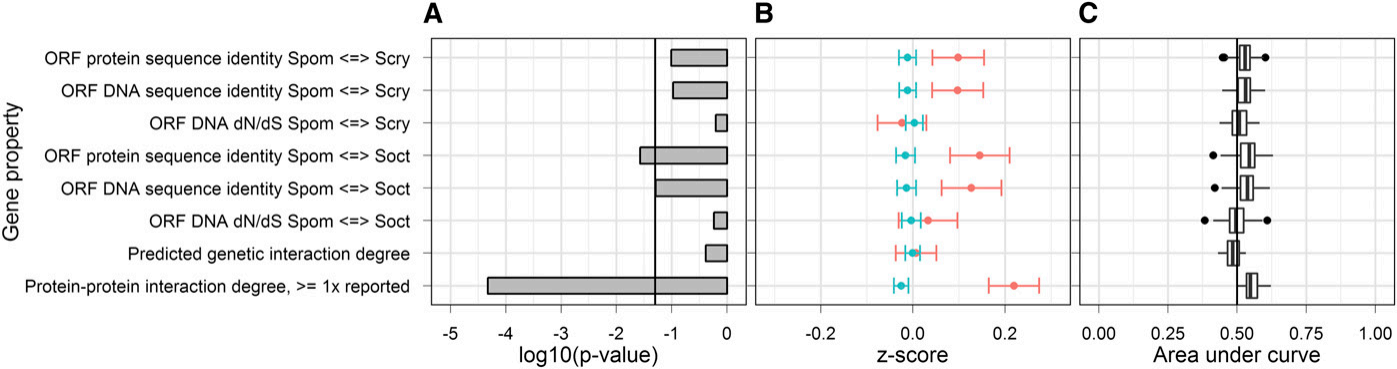
Relationships between HI and non-HI gene properties for *Sz. pombe* in rich medium. (A) The *p*-values testing the difference between HI and non-HI gene property value distributions. These are on a log10 scale and are as estimated by the Mann-Whitney *U*-test. The vertical line shows a *p*-value of 0.05. (B) Mean *z*-scores of HI (red) and non-HI (blue) gene properties. Error bars represent the SEM. (C) The receiver-operating characteristic (ROC) area under curve (AUC) distributions. These were generated using cross-validation (see Materials and Methods). Whiskers represent the lowest point within 1.5 interquartile range (IQR) of the lower quartile and the highest point within 1.5 IQR of the upper quartile. Dots represent outliers of the aforementioned ranges. The vertical line in the center of the chart represents the random expectation for the ROC plot.

In addition to our work performed on *S. cerevisiae*, we examined the available HI data for *Sz. pombe* (Kim *et al.* 2010). In particular, we looked at relationships between HI and DNA sequence conservation, PPI degree from BioGRID (Stark 2006), and predicted GI degree from a recent study (Koch *et al.* 2012). Other biological properties were not examined because of unavailability of comprehensive data in *Sz. pombe*. To determine gene sequence conservation, we compared the ORF sequence data between *Sz. pombe* and two closely related species, *Sz. octosporus* and *Sz. cryophilus*. Using genome annotations, we performed ORF alignments and calculated sequence conservation statistics before comparing these statistics against *Sz. pombe* HI data. Interestingly, we found significant positive associations with HI for DNA and protein sequence identity between *Sz. pombe* and both *Sz. octosporus* and *Sz. cryophilus*, although correlation between HI and *dN*/*dS* was insignificant (Figure 6). We also found a significant moderate positive relationship between HI and PPI degree. The association between HI and predicted GI degree was not significant. In general, these preliminary results suggest that our HI prediction method based on the biological properties of the cell could be applied to other microorganisms.

We also examined the level of HI phenotype conservation between *S. cerevisiae* and *Sz. pombe* ortholog genes. A set of 2372 *Sz. pombe* genes was considered. This set contained 279 *Sz. pombe* and 93 *S. cerevisiae* HI genes. The set also only contained those genes with no duplicate gene orthologs in *S. cerevisiae* and was such that rich medium heterozygous-null mutant phenotypes were available from both organisms. We found that only 17 out of 2372 genes were HI in both *S. cerevisiae* and *Sz. pombe*. This result shows that orthology is not informative of HI in these two yeasts because HI does not tend to be conserved between members of orthologous gene pairs.

## Discussion

In this work, we identified several gene properties with significant differences between HI and non-HI genes. We exploited these associations by building a machine-learning model that prioritizes candidate genes according to their inferred HI probabilities. By prioritizing candidate genes, we discovered novel HI phenotypes that were not detected previously in genome-scale screens. In addition, the relationships uncovered here between HI and gene properties provide useful clues in terms of understanding HI mechanisms.

The relationships identified here show that HI genes tend to be highly expressed and highly conserved, with large numbers of genetic and physical interactions. We have also shown that HI genes tend to have slightly less conservation at the promoter and slightly less cell-cycle regulation. The reduced cell-cycle regulation suggests that HI genes tend to be constitutively expressed (*i.e.*, “always on”), at least in terms of the cell cycle. One would expect hemizygosity of a constitutively expressed gene to cause a greater reduction in protein expression than a more highly regulated gene, because there are probably fewer regulatory mechanisms to respond to gene copy number variation in constitutively expressed genes. The relationship between transcriptional regulation and HI could be explored more fully by examining microarray data beyond the cell cycle.

We found that PPI degree associates most strongly with HI in rich medium. This relationship supports the idea that gene dosage is important for viability of protein complexes. During construction of our model, we tested three PPI networks, each of which was generated from physical interaction data in BioGRID. These included an unprocessed interaction network along with two stringent networks that only considered interactions reported at least two or three times. Interestingly, the unprocessed network performed best. We think the stringent networks performed less well because considerable amounts of data are excluded during the filtering process. Approximately 80% of interactions are removed when only considering interactions reported at least twice, whereas approximately 90% are removed when only considering interactions reported at least three times.

Our study demonstrates that some HI phenotypes are missing from existing genome-scale data. This could be attributable to interactions between heterogeneous strains sharing the same environment, disagreements between biological replicates, or assay sensitivity limitations. For example, there could be a dependency of HI phenotypes on the structure of the competing population and from general growth conditions. In fact, competition environments in which multiple mutants are grown together can be rather different from medium containing only monocultures or two strains. Six thousands mutants competing in the same nutritional context are likely to excrete several different types and quantities of metabolites (Allen *et al.* 2003), which could affect the growth of the population both in the negative direction and in the positive direction. Although the extent of this cross-feeding effect is not known, obtaining genome-scale monoculture HI profile data would be a useful step toward understanding it.

The level at which false-positives are present in large-scale genomic data is also not entirely known. Deutschbauer *et al.* (2005) tested 30 HI genes in individual growth assays to prove the validity of the molecular bar-coding to reproducibly score quantitative fitness differences as small as 2%.

HI phenotype differences could also arise from differences between experimental methods. This study and the two previous studies (Deutschbauer *et al.* 2005; Delneri *et al.* 2008) each used different experimental methods and metrics to identify HI genes. In the Deutschbauer *et al.* (2005) study, a batch serial dilution competition approach was used. Two independently constructed pools of hemizygous strains were diluted at five generation intervals. Hybridization tags were used in combination with a microarray and a regression-based approach to quantify differences between the growth rates of strains relative to WT. Genes were considered significantly HI when both replicates had a fitness value less than 0.95 and at least one tag in both replicates had a *p*-value < 0.05.

The Delneri *et al.* (2008) work used competition experiments in continuous cultures to identify HI genes via hybridization of the strain bar codes. The advantage of this method was that both growth rate and pH are kept constant, allowing a more sensitive identification of HI phenotypes. To determine significant HI strains, a regression-based method was used, followed by calculation of *p*-values. False discovery rates were estimated using the Benjamini-Hochberg method to produce *Q*-values. Growth rates with *Q* < 0.01 were considered statistically significant.

Both of these studies, based on strain-specific tag hybridization, are sensitive and quantitative in nature for the determination of HI. In our study, we measured growth curves of monocultures and used the difference between mutant and WT AUGC means as a quantitative measure of fitness. To determine the significance of the difference between these means, we calculated *p*-values using the Welch *t*-test, followed by correction using the Benjamini–Hochberg procedure. Despite the fact that growth curves are typically less sensitive than genome-wide competition experiments to detect fitness declines, we were successful in identifying new HI phenotypes.

Our gene properties associate most strongly with rich medium HI, with weaker relationships between gene properties and HI in minimal, nitrogen-limited, carbon-limited, phosphate-limited, and grape juice media. For some gene properties, this might be because property data were produced largely in experiments that used rich medium. Properties potentially affected by environmental conditions include PPI and GI degree, protein expression level, and magnitude of cell-cycle regulation. For example, the PPI data mostly comprise interactions reported through affinity capture methods, which are affected by the nutrient environment. The strong associations with HI in rich medium may also be attributable to the laboratory strains being more adapted to rich medium, thus resulting in gene properties showing stronger relationships in this environment. The associations between gene properties and HI are much weaker for carbon-limited, nitrogen-limited, phosphate-limited, and grape juice environments. This may be a result of different experimental conditions in the corresponding study (Delneri *et al.* 2008); in that work, the cell culture was held at maximum growth rate, whereas the rich medium study (Deutschbauer *et al.* 2005) and our study have not attempted to do this. In general, however, more experimental HI phenotype data, ideally using different strain backgrounds, would be necessary to determine the extent to which these conditions might affect HI phenotypes detected.

To investigate the sensitivity of our method for detecting small fitness differences, we selected the gene *RPN1* as a “weak” positive control. This gene had a subtle but significant HI phenotype in a previous rich medium study (fitness difference for *RPN1* = 0.971 *vs. TUB1* = 0.921 and *RPL25* = 0.818 for our two “strong” positive controls). When we tested this gene for HI using our growth curve assay, the fitness loss registered was very small and therefore not significant. This undetected HI might be either a consequence of the limited sensitivity of the method compared to the genomic bar code analysis or a consequence of the lack of yeast competitors in the monoculture. An improved sensitivity for HI phenotypes might, in fact, be achieved via one-to-one competition experiments, although such experiments could be affected by cross-feeding interactions between the reference strain and the mutant. These results show that our experimental validation of the model is likely to underestimate the HI phenotypes and is a conservative evaluation of the predictive power of our method.

Ribosomal and core cellular process genes were heavily over-represented in the earlier rich medium study (Deutschbauer *et al.* 2005). Our six newly identified rich medium HI genes include two ribosomal genes (*RPS6B* and *RPL10*), a proteasome gene (*RPN10*), and three core cellular process genes (*BCY1*, *BRE5*, and *GCN5*), one of which is an enzyme (*GCN5*).

To see whether our rich medium phenotypes were displayed in other environments, we performed additional experiments in different nutrient-limited conditions using our 23 candidate genes. Five candidates were found to be HI in F1 medium with nitrogen limitation, and only one HI gene was detected in F1 medium with carbon limitation. These data suggest that there is some overlap of HI phenotypes between YPD and nitrogen-limited media, whereas in the carbon-limited medium the overlap is smaller. This observation was not unexpected because the HI genes in nitrogen-limited medium associate moderately well with the various gene properties in the model, whereas carbon-limited HI does not (Figure S1, C and D). One of the genes we found to be HI in nitrogen-limited medium, *SEC28*, is not HI in rich medium. Sec28p has been previously shown to interact with and stabilize Cop1p (Duden *et al.* 1998), and *COP1* was found to be HI in nitrogen-limited medium previously (Delneri *et al.* 2008). These results suggest that the genes *SEC28* and *COP1* might have a shared role in nitrogen stress response.

Previous work (Deutschbauer *et al.* 2005) showed that HI genes have a higher likelihood of being essential than other genes. Because PPI degree is known to correlate with gene essentiality (Jeong *et al.* 2001; He and Zhang 2006; Zotenko *et al.* 2008), we set out to investigate the level by which gene essentiality is a confounding factor between HI and our chosen gene properties. We divided genes into essential and nonessential groups and then examined the associations between gene properties and HI within those groups. For PPI degree, gene sequence conservation, cell-cycle expression variation, and protein abundance, relationships between HI and gene properties were significant among both essential and nonessential genes. This demonstrates that associations between these properties and HI cannot fully be explained by the over-representation of gene essentiality among HI genes. We therefore suggest that HI and gene essentiality are, in part, independent phenomena.

Promoter sequence identity was an exception among the properties tested; although it associated significantly with HI among nonessential genes, the relationship between essential gene HI and this variable was insignificant. This might be attributable to either the overall weak association between HI and promoter sequence identity or a result of gene essentiality fully explaining the relationship between promoter conservation and HI. We opted to leave promoter sequence identity in the model because it improved predictive performance.

Availability of gene property data in other organisms may allow their HI to be predicted. We show that this may be possible in *Sz. pombe* by demonstrating positive relationships between *Sz. pombe* HI and both PPI degree and ORF sequence conservation. The sequence conservation associations, although significant, were only weak. This may reflect the fact that the two *Schizosaccharomyces* clade members used to calculate conservation are more distantly related to *Sz. pombe* than the *Saccharomyces sensu stricto* clade members are to *S. cerevisiae*. This greater evolutionary distance may cause signal loss when calculating sequence conservation statistics for *Sz. pombe*. Evidence supporting this argument includes the fact that DNA sequence conservation associates less with HI than protein sequence conservation. Additionally, there is no significant relationship between *dN*/*dS* and HI in *Sz. pombe*. The association between PPI degree and HI in *Sz. pombe* is weaker than the equivalent in *S. cerevisiae*, possibly because the *Sz. pombe* interaction dataset is less complete than that of *S. cerevisiae*. In accordance with the Kim *et al.* (2010) study, we found low HI profile conservation between *S. cerevisiae* and *Sz. pombe* orthologs, suggesting that methods that implement prediction of HI based on gene properties could be crucial for identifying HI genes *a priori* in different species. It is worth noting that *Sz. pombe* only becomes diploid transiently, just before meiosis, and therefore the biological significance of HI for this organism may be limited to a defined time interval.

In conclusion, we have shown significant relationships between gene properties and HI phenotypes scored in rich medium. We have used these associations to create a model to identify novel HI genes, and we experimentally identified six new hemizygous mutant strains with compromised fitness in rich medium, along with five HI phenotypes in F1 medium with nitrogen limitation and one HI phenotype in carbon-limited medium. This method could be applied to uncover HI phenotypes in other species.

## Supplementary Material

Supporting Information
