# Supplementary material for: Characterization and Prediction of Haploinsufficiency Using Systems-Level Gene Properties in Yeast
Source: G3 (Bethesda). 2013 Nov 1;3(11):1965–77. doi: 10.1534/g3.113.008144 (PMC3815059; doi:10.1534/g3.113.008144)
Supplement: Supporting Information [file supp_3_11_1965__index.html]

Characterization and Prediction of Haploinsufficiency Using Systems-Level Gene Properties in Yeast — Supporting Information 

# Characterization and Prediction of Haploinsufficiency Using Systems-Level Gene Properties in Yeast

## Supporting Information for Norris, Lovell, and Delneri, 2013

**Files in this Data Supplement:**

- Supporting Information - Figures S1-S4, Files S1-S2, and Tables S1-S2 (PDF, 2 MB)
- Figure S1 - Relationships between HI and non-HI gene properties across 6 environments. (PDF, 1 MB)
- Figure S2 - Pearson's product-moment correlation coefficients between 8 gene properties. (PDF, 317 KB)
- Figure S3 - FPR < 0.1 AUC distribution across all combinations of gene properties, using 5 missing value handling methods. (PDF, 1 MB)
- Figure S4 - Growth curves for all strains found to be significantly unfit in our study. (PDF, 497 KB)
- Table S1 - Table describing three letter abbreviations, descriptions and data sources for gene properties shown in Figure S4. (PDF, 423 KB)
- Table S2 - Table showing phenotypes of hemizygous strains experimentally tested in this work. (PDF, 465 KB)
- File S1 - Supplementary Dataset 1 (.xlsx, 1 MB)
- File S2 - Supplementary Dataset 2 (.xlsx, 859 KB)
