# Supplementary material for: Characterization and Prediction of Haploinsufficiency Using Systems-Level Gene Properties in Yeast
Source: G3 (Bethesda). 2013 Nov 1;3(11):1965–77. doi: 10.1534/g3.113.008144 (PMC3815059; doi:10.1534/g3.113.008144)
Supplement: Supporting Information [file supp_g3.113.008144_008144SI.pdf]

G3·Genes | Genomes | Genetics

Characterization and prediction of haploinsufficiency using systems-level gene properties in yeast

Matthew Norris[1], Simon Lovell[1] and Daniela Delneri[1]

[1] Faculty of Life Sciences, The University of Manchester, Manchester, Lancashire, M13 9PT, United Kingdom

DOI: 10.1534/g3.113.008144

(A) Rich medium (YPD), from Deutschbauer et al. (2005).

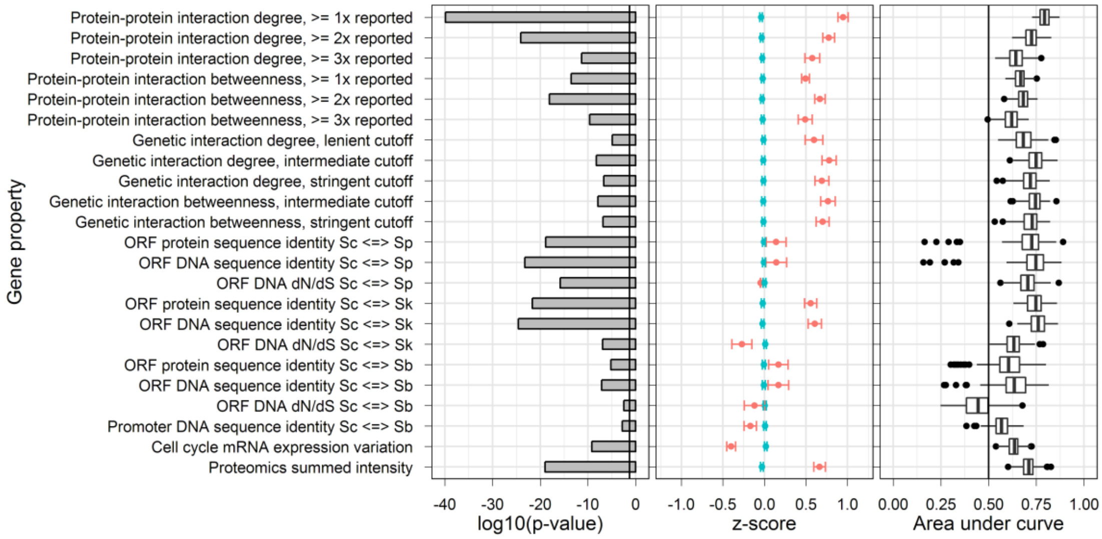

(B) Minimal medium (MM), from Deutschbauer et al. (2005).

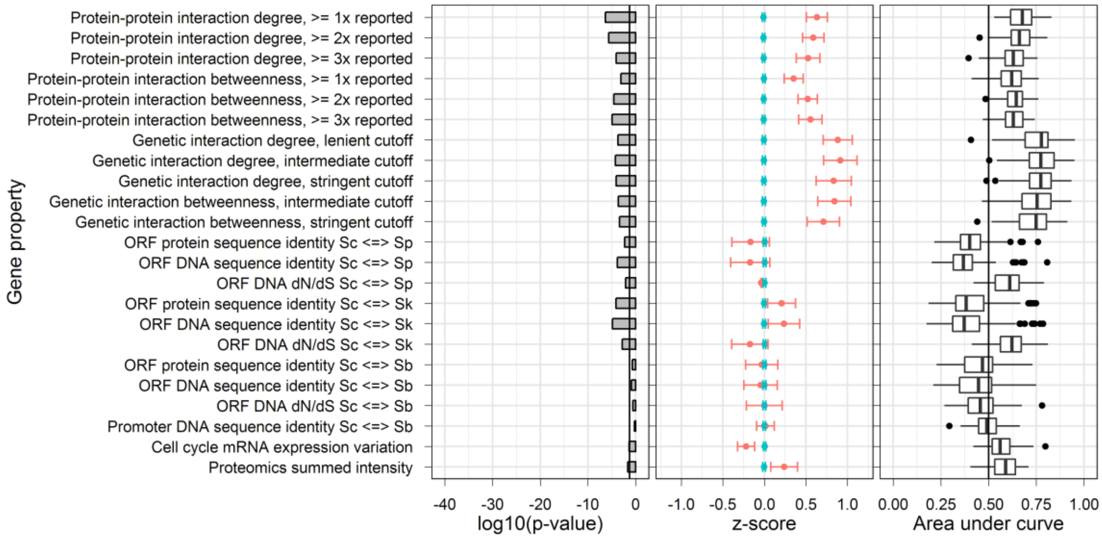

(C) F1 medium with carbon limitation. From Delneri et al. (2008).

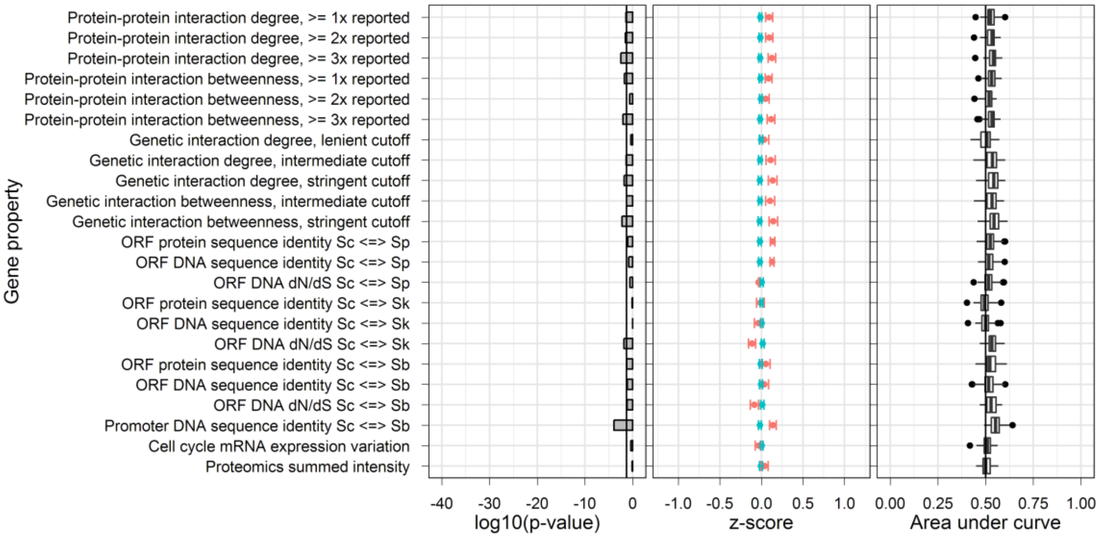

(D) F1 medium with nitrogen limitation. From Delneri et al. (2008).

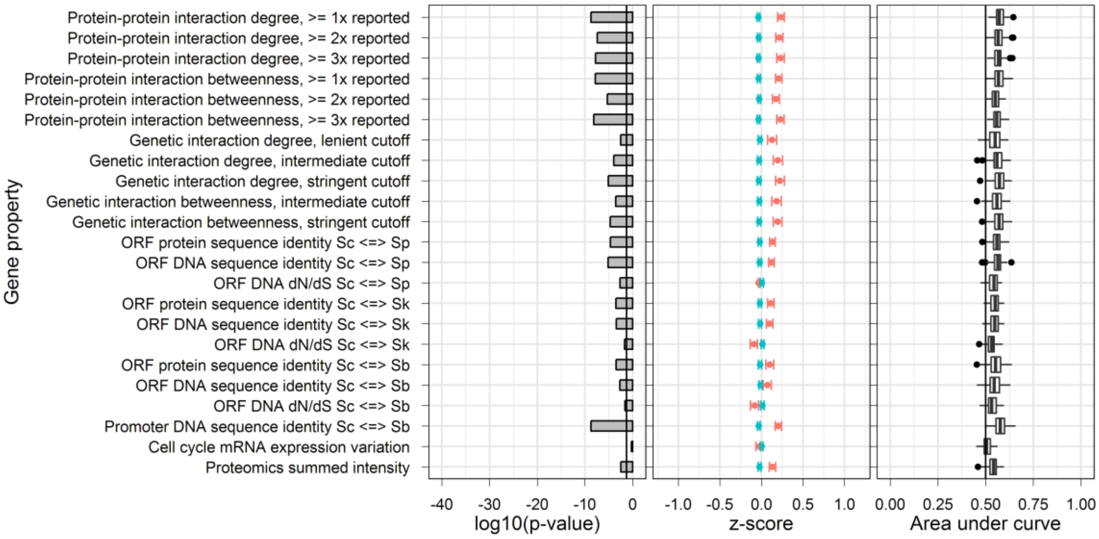

(E) F1 medium with phosphate limitation. From Delneri et al. (2008).

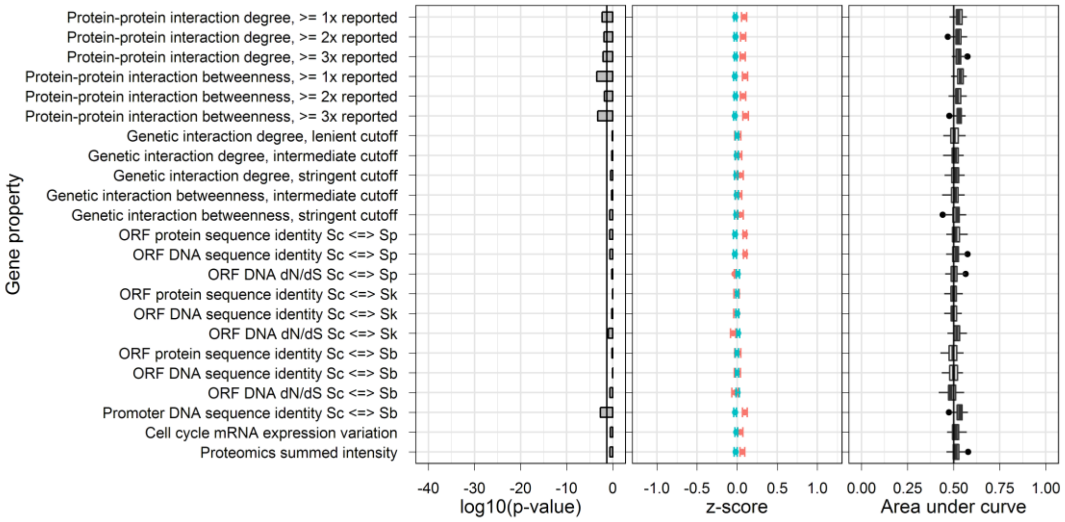

(F) White grape juice. From Delneri et al. (2008).

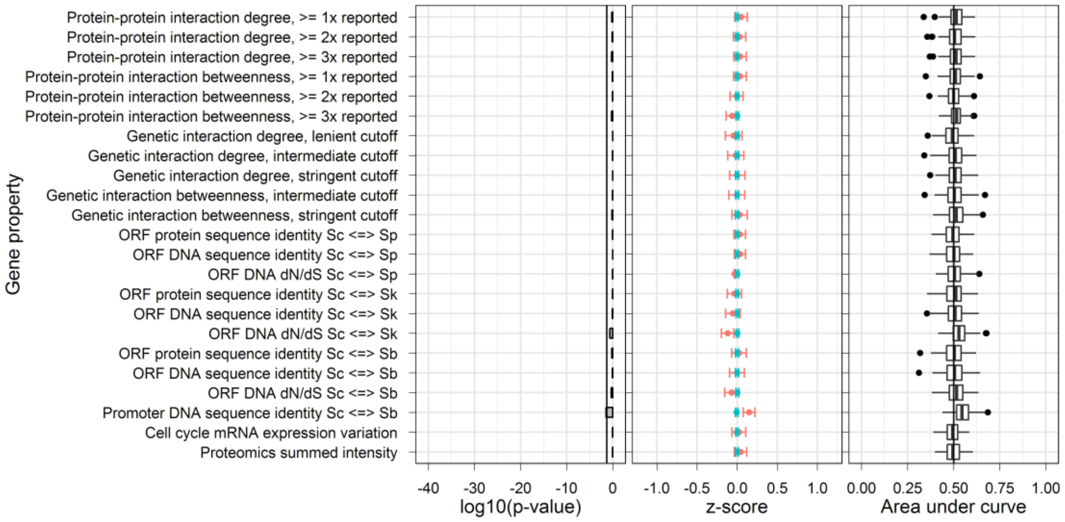

Figure S1 Relationships between HI and non-HI gene properties across 6 environments. Environments include (A) rich medium, (B) minimal medium, (C) F1 medium with carbon limitation, (D) F1 medium with nitrogen limitation, (E) F1 medium with phosphate limitation, (F) white grape juice. Left panel: Distribution differences are tested using *p*-values on a log10 scale, as estimated by the Mann-Whitley U test. The vertical line shows a *p*-value of 0.05. Center panel: Mean *z*-scores of HI (red) and non-HI (blue) gene properties are shown. Error bars represent the standard error of the mean. Right panel: ROC curve AUC distributions. These were generated using cross validation (see Methods). Whiskers represent lowest point within 1.5 interquartile range (IQR) of the lower quartile, and highest point within 1.5 IQR of the upper quartile. Dots represent outliers of the aforementioned ranges. The vertical line in the center of the chart represents the random expectation for the ROC plot.

	Protein abundance	Cell cycle mRNA variation	Promoter sequence identity	ORF DNA sequence identity	GI network betweenness	GI degree	PPI network betweenness	PPI network degree
Protein abundance								
Cell cycle mRNA variation	-0.1							
Promoter sequence identity	0.05	0.06						
ORF DNA sequence identity	0.14	-0.1	-					
GI network betweenness	0.06	-0.1	0.06	0.11				
GI degree	0.07	-0.1	0.06	0.12	0.98			
PPI network betweenness	0.26	-	0.04	0.11	0.24	0.27		
PPI network degree	0.34	-	0.05	0.15	0.24	0.26	0.7	

Figure S2 Pearson’s product-moment correlation coefficients between 8 gene properties. ”-“ denotes cases where no correlation was detected, i.e. a *p* value > 0.05.

(A) Roll back to a simpler model.

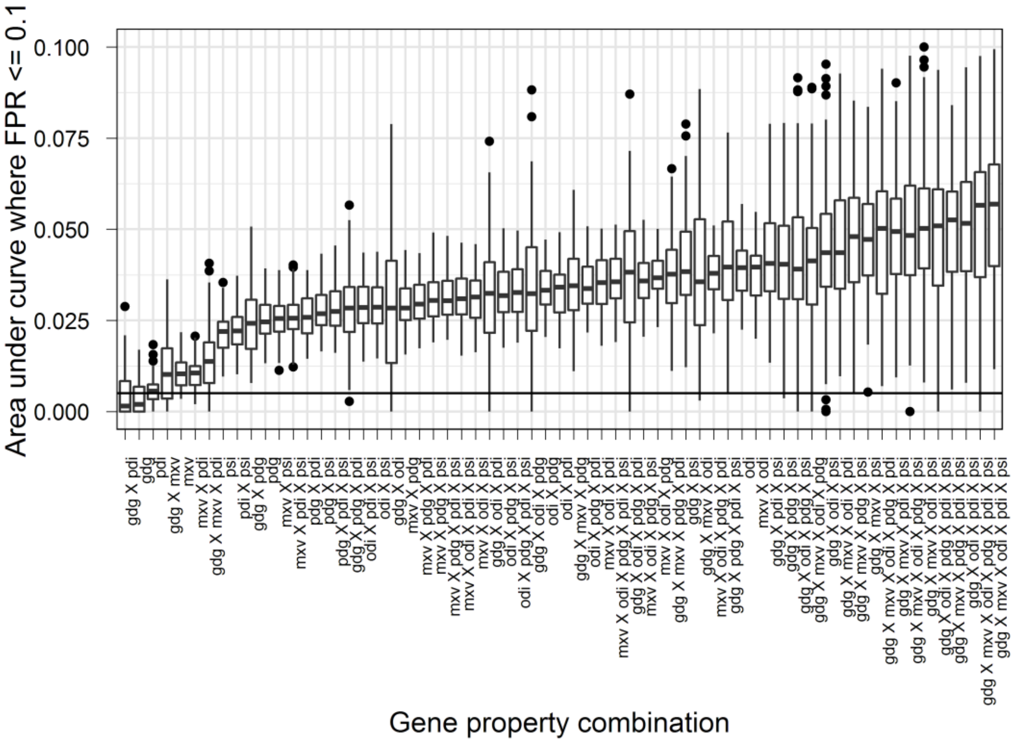

(B) Exclude incomplete cases.

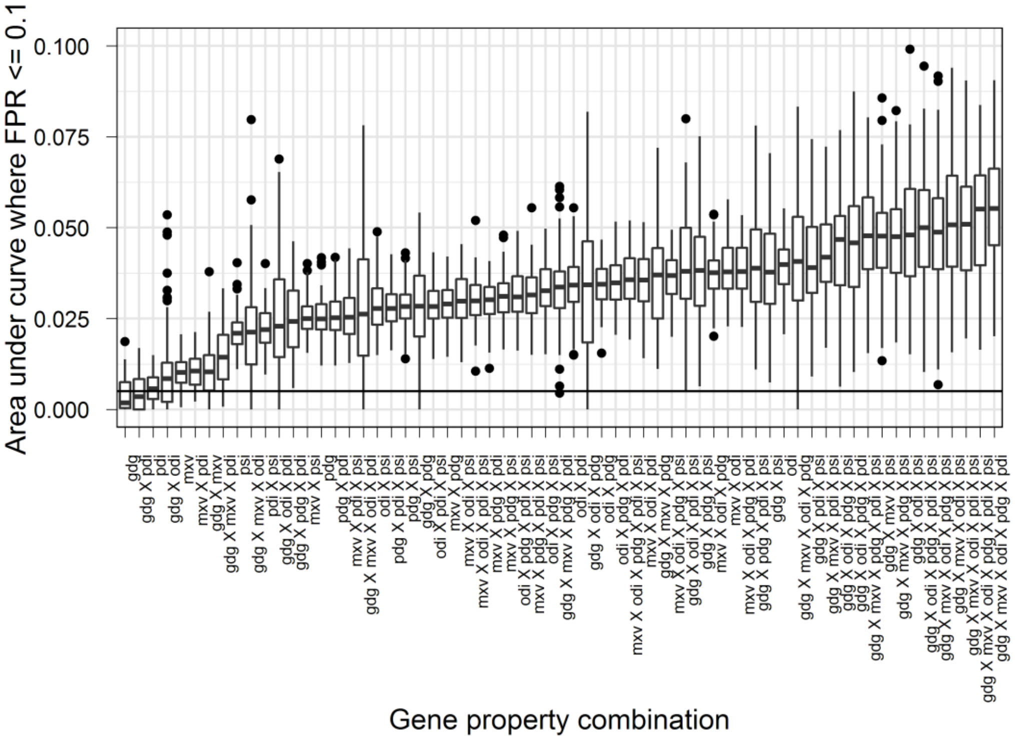

(C) Expectation-maximisation algorithm.

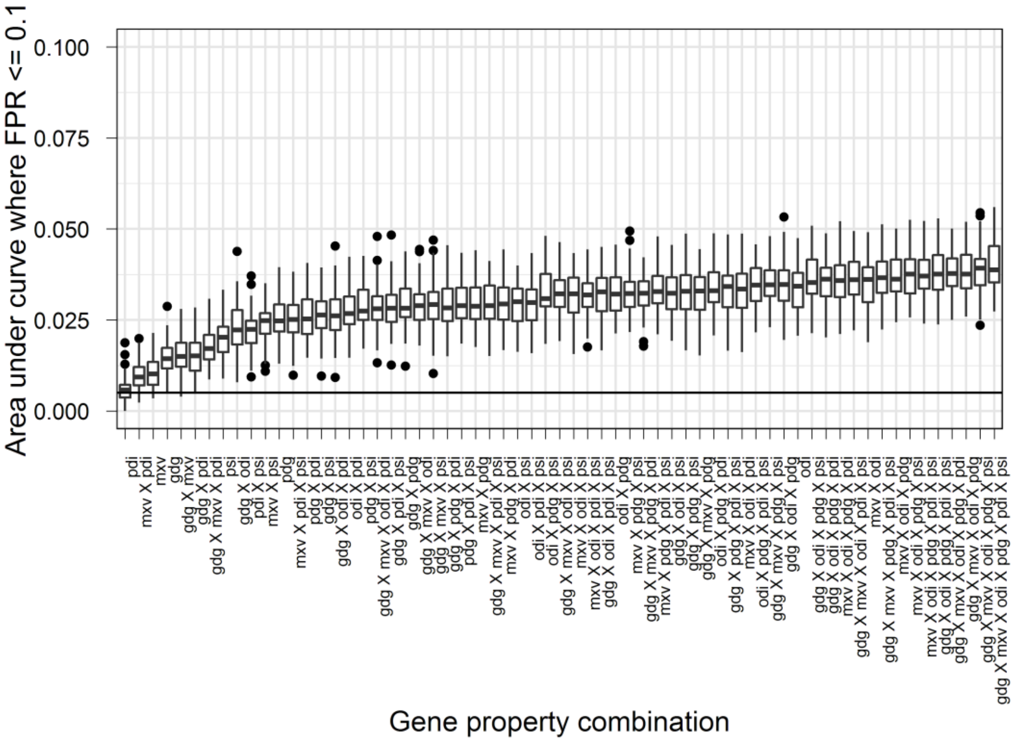

(D) Multiple imputation.

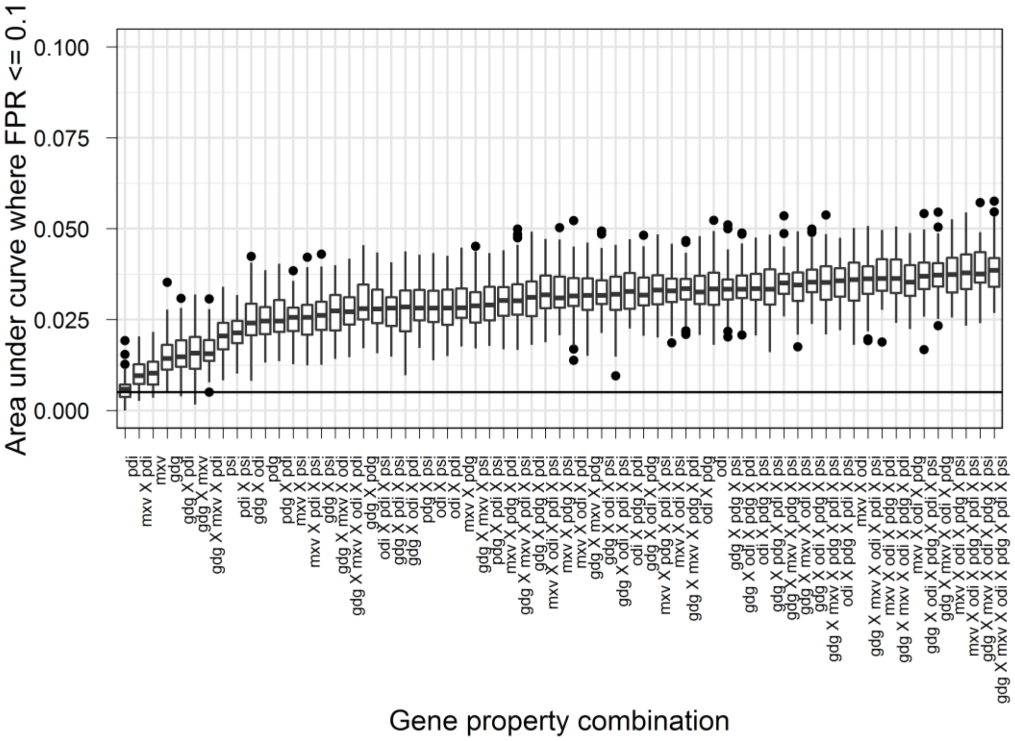

(E) Median imputation.

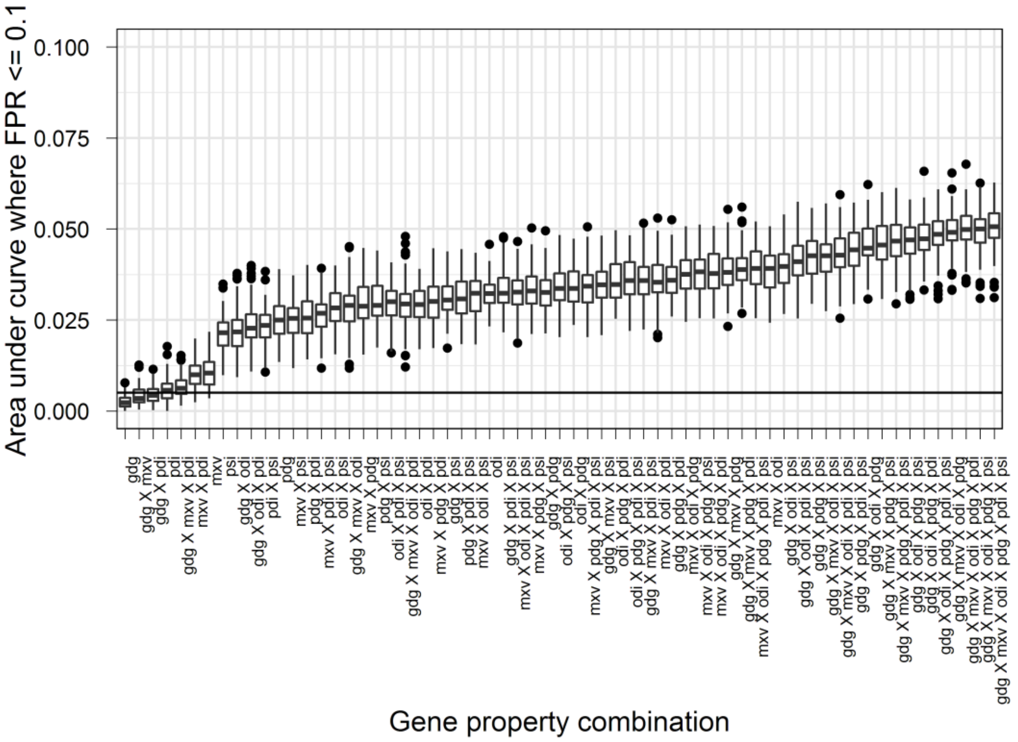

Abbreviation	Description
gdg	Genetic interaction network degree
mxv	mRNA expression variation through the yeast cell cycle
odi	ORF DNA percentage between *S. cerevisiae* and *S. kudriavzevii*
pdg	Protein interaction network degree
pdi	Promoter sequence DNA identity between *S. cerevisiae* and *S. paradoxus*
psi	Summed intensities, representing combined haploid and diploid protein abundance.

Figure S3 FPR < 0.1 AUC distribution across all combinations of gene properties, using 5 missing value handling methods. Methods for missing value treatment include (A) rolling back to a simpler model, (B) excluding incomplete cases, (C) the expectation-maximisation algorithm, (D) multiple imputation, and (E) median imputation. The three letter codes identify gene properties and are described in the legend. Distributions are for 100 ROC curves generated during cross validation (see Methods). Whiskers represent lowest point within 1.5 interquartile range (IQR) of the lower quartile, and highest point within 1.5 IQR of the upper quartile. Dots represent outliers of the aforementioned ranges. The black horizontal line represents the random expectation from the ROC plot.

A - Candidate genes in rich medium.

(A1)

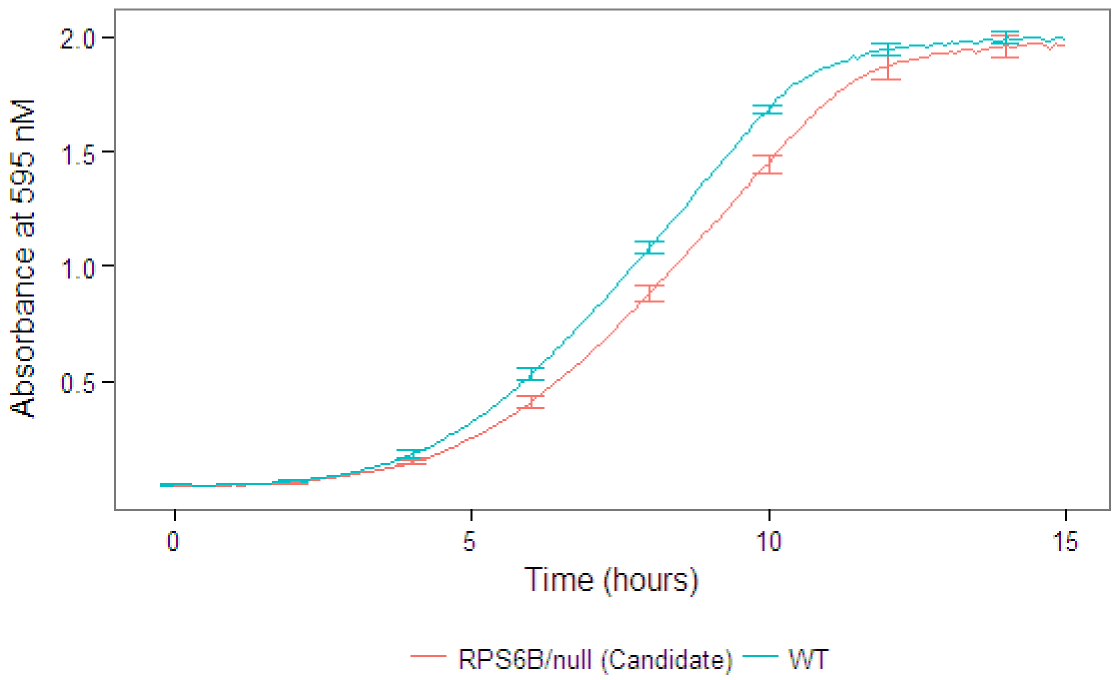

(A2)

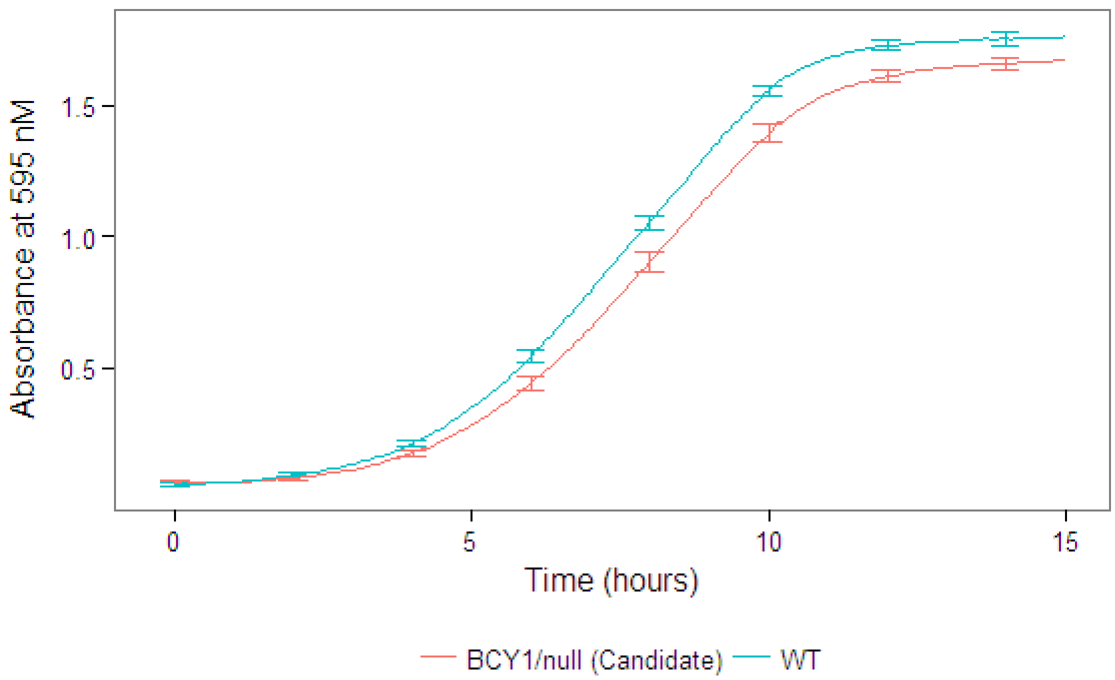

(A3)

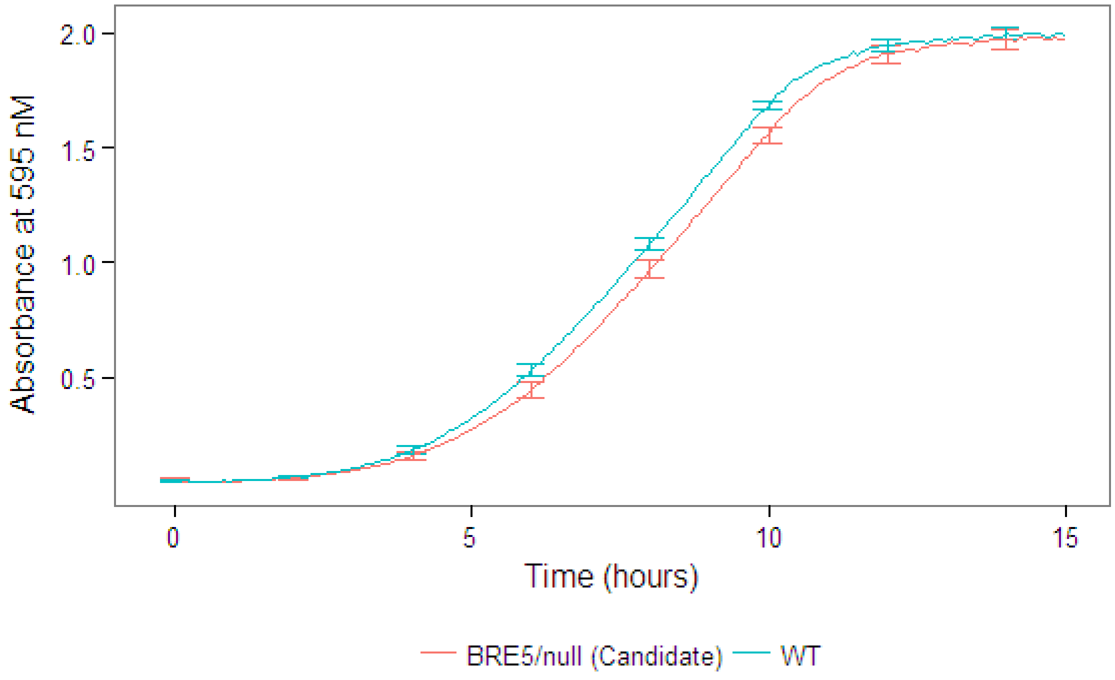

2.0
1.5
1.0
0.5
Absorbance at 595 nM
0
5
10
15
Time (hours)
BRE5/null (Candidate)
WT

(A4)

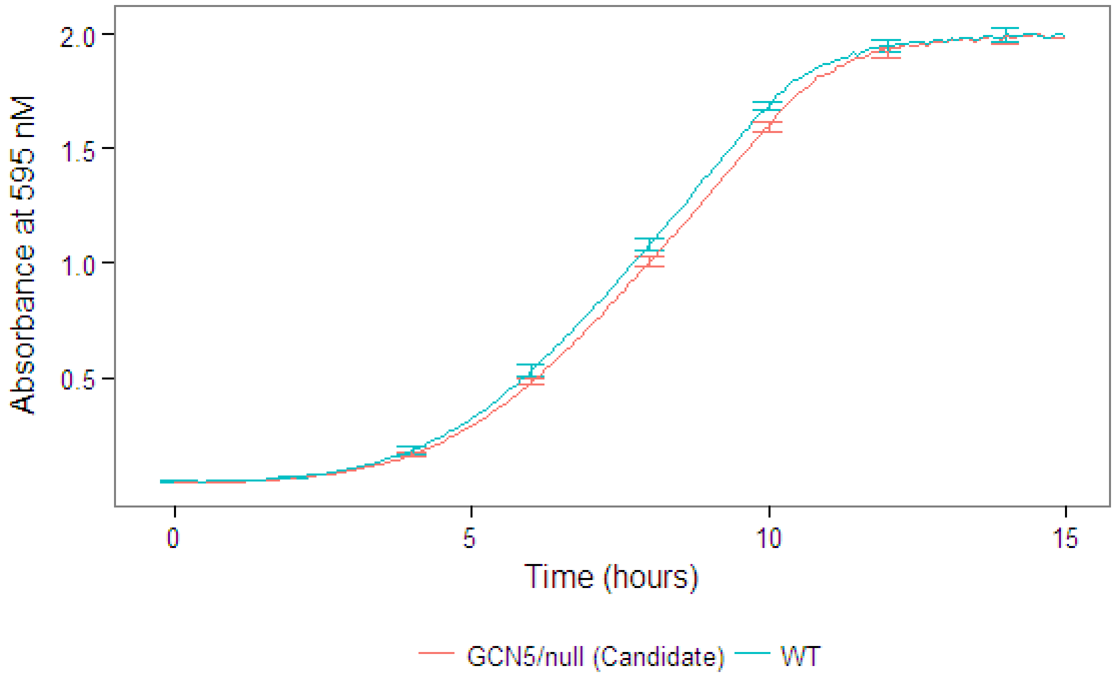

2.0
1.5
1.0
0.5
Absorbance at 595 nM
0
5
10
15
Time (hours)
GCN5/null (Candidate)
WT

(A5)

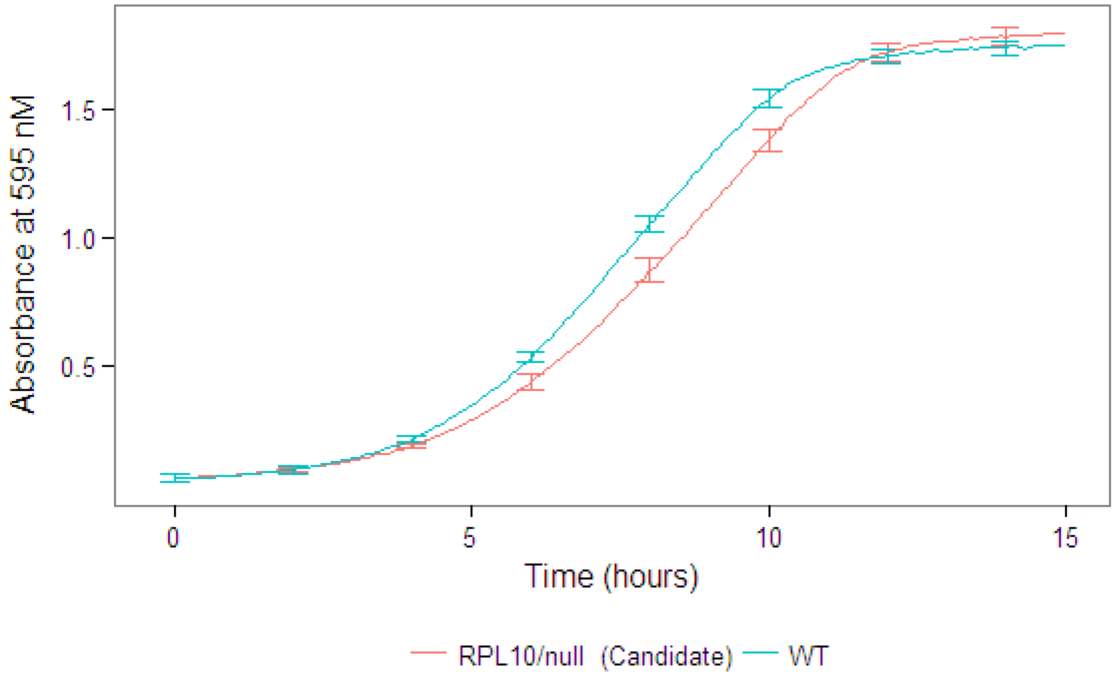

Absorbance at 595 nM
0.5
1.0
1.5
0
5
10
15
Time (hours)
RPL10/null (Candidate)
WT

(A6)

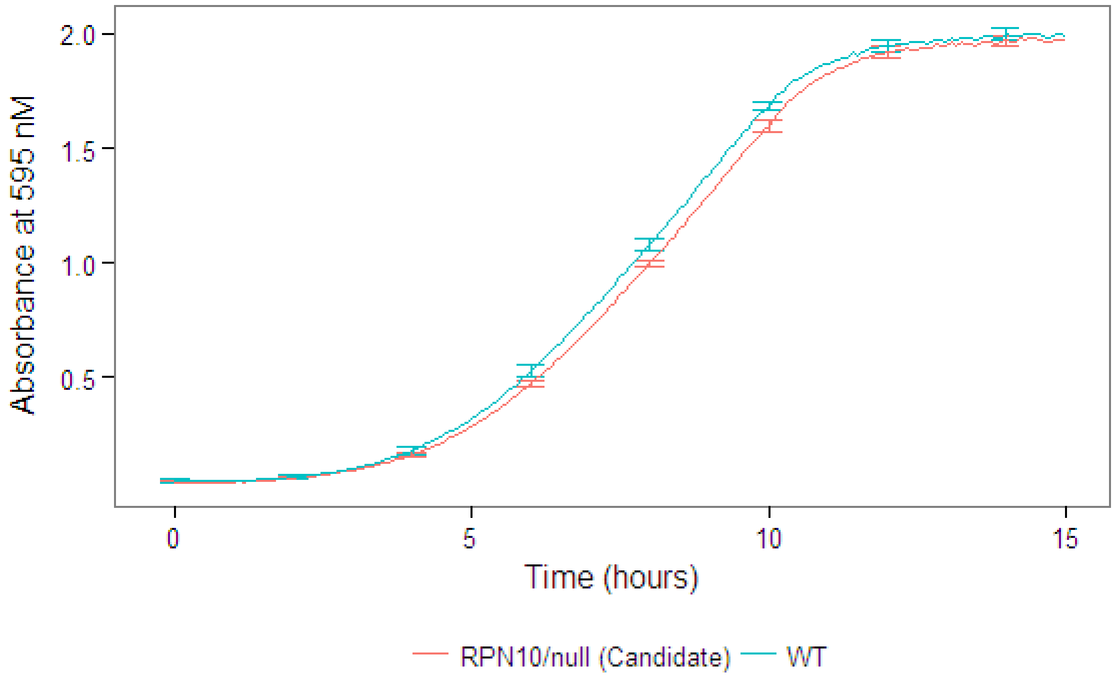

Absorbance at 595 nM
0.5
1.0
1.5
2.0
0
5
10
15
Time (hours)
RPN10/null (Candidate)
WT

B – *Candidate genes in nitrogen-limited medium.*

(B1)

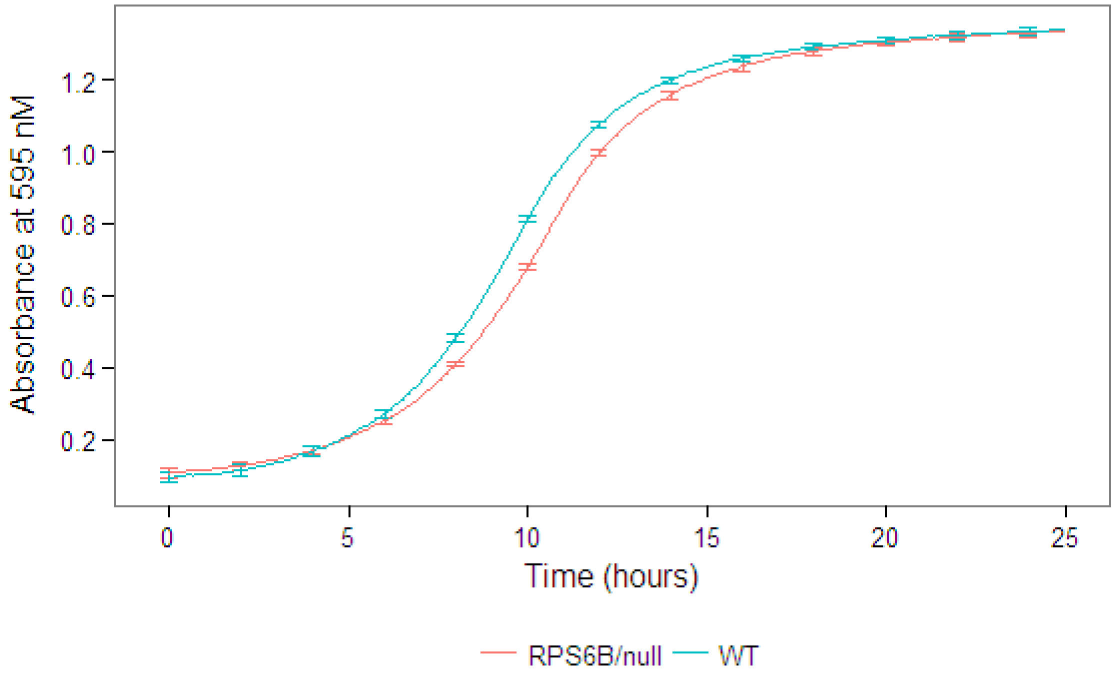

(B2)

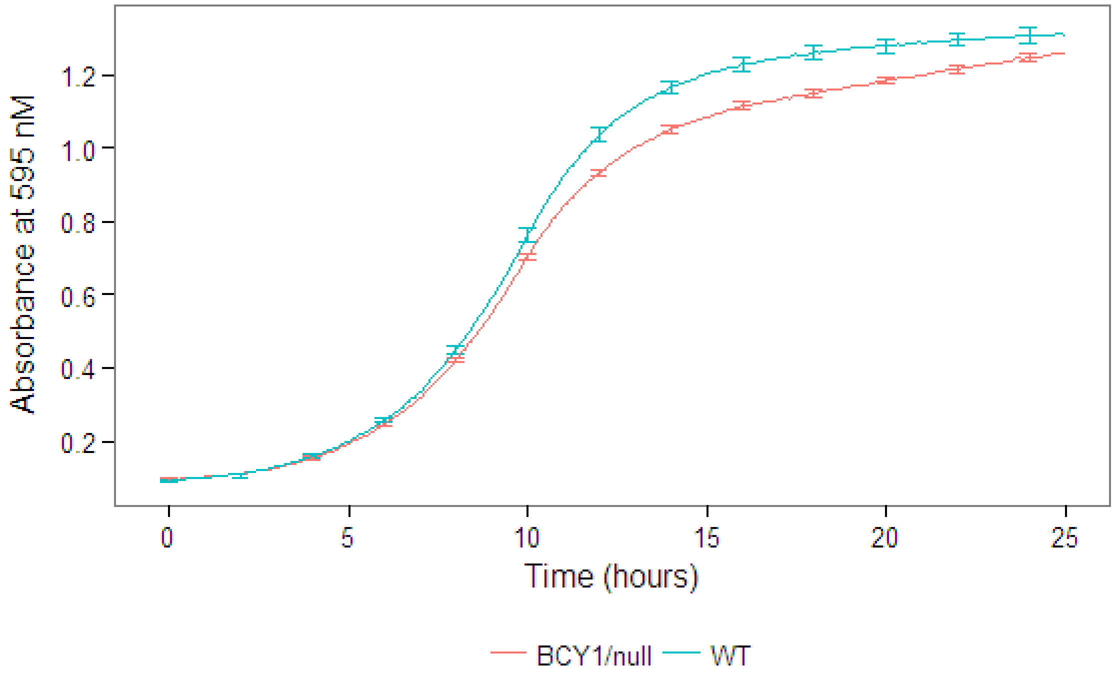

(B3)

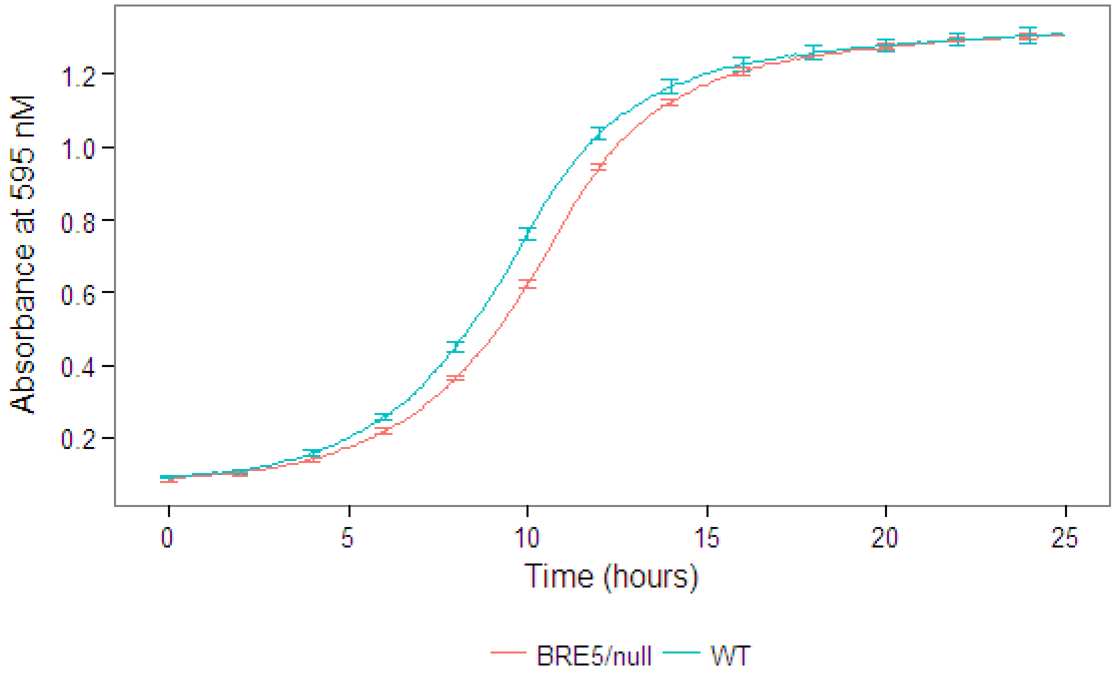
1.2
1.0
0.8
0.6
0.4
0.2
Absorbance at 595 nM
0
5
10
15
20
25
Time (hours)
BRE5/null
WT

(B4)

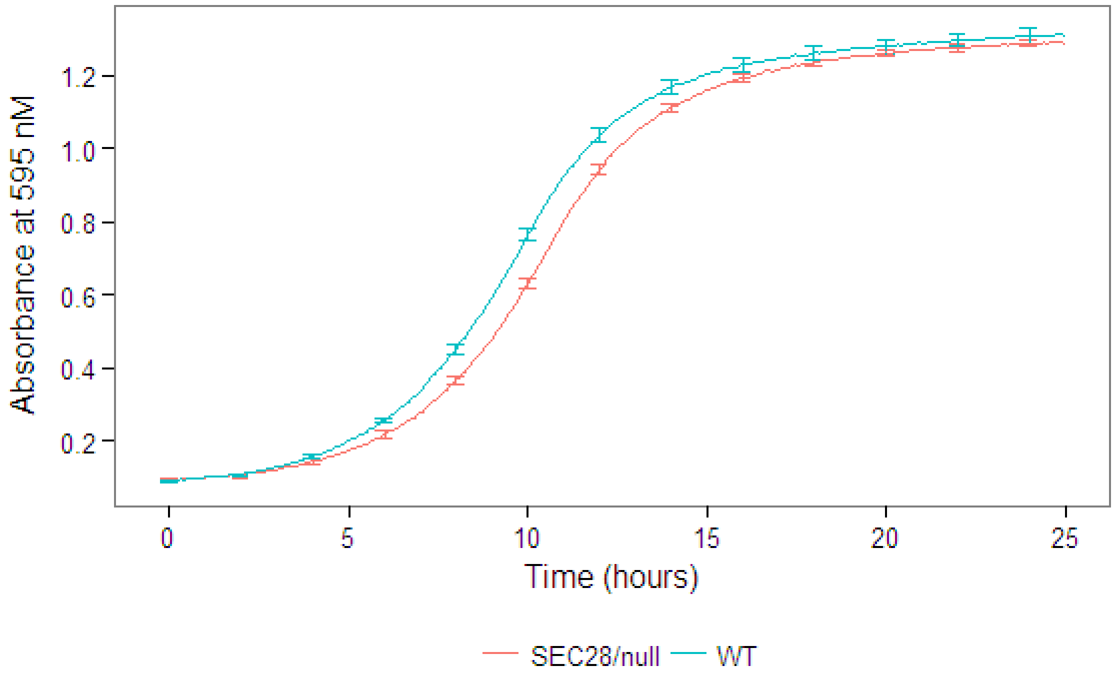
1.2
1.0
0.8
0.6
0.4
0.2
Absorbance at 595 nM
0
5
10
15
20
25
Time (hours)
SEC28/null
WT

(B5)

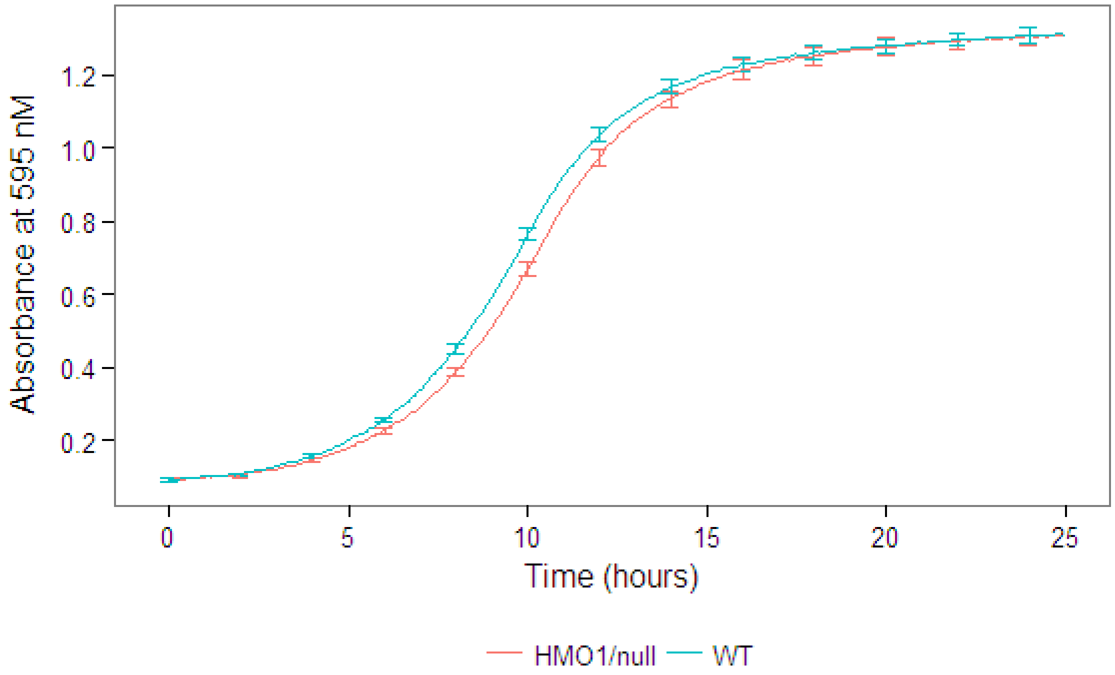

***C** – Candidate gene in carbon-limited media.*

(C)

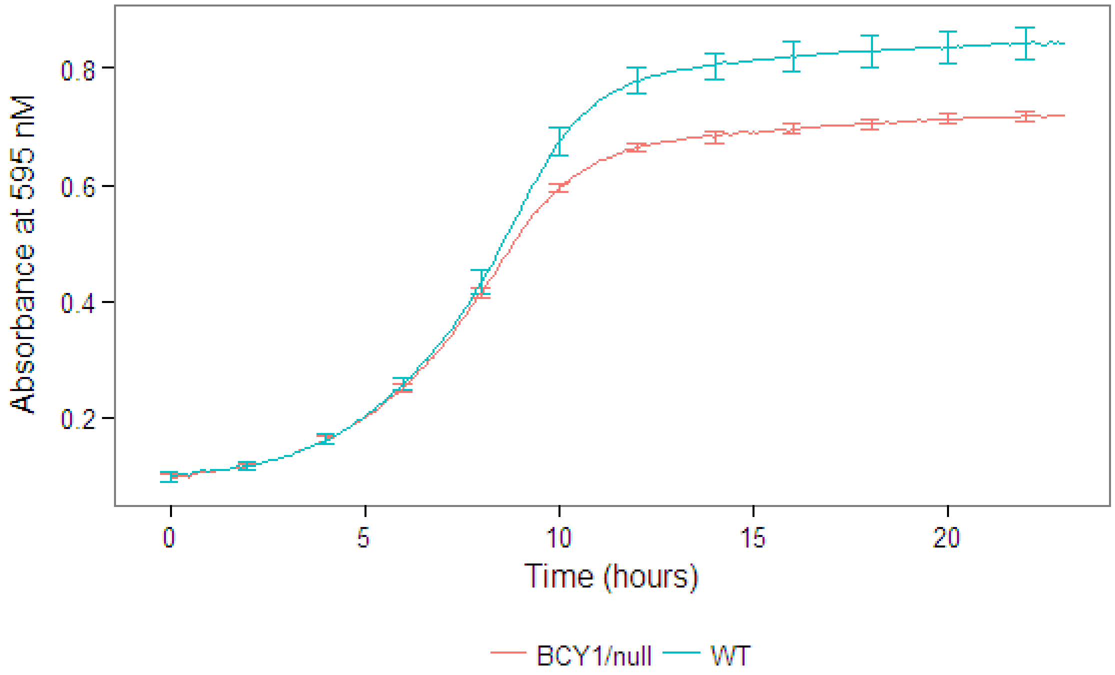

D – *Background control gene in rich medium.*

(D)

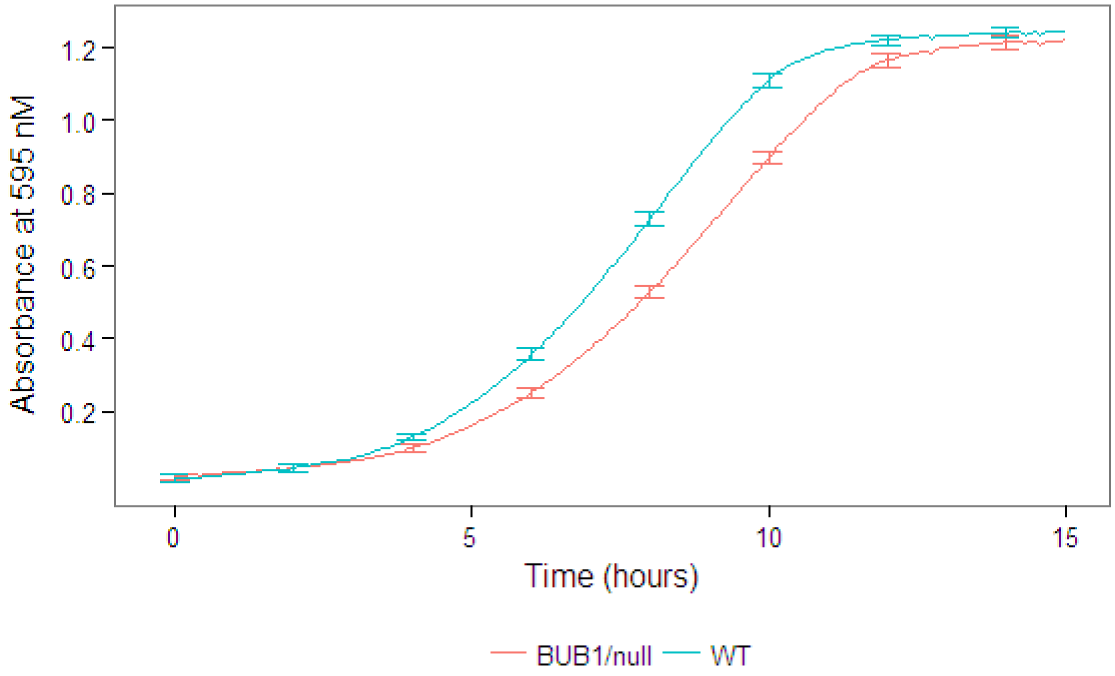

E *– Positive control genes in rich medium.*

(E1)

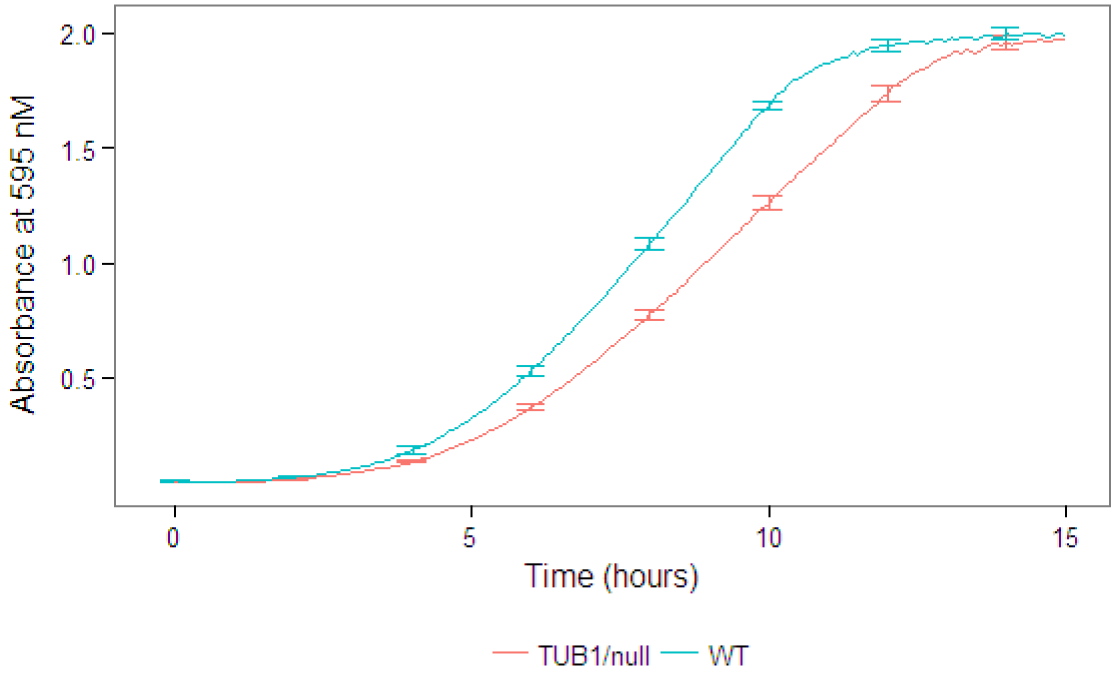

(E2)

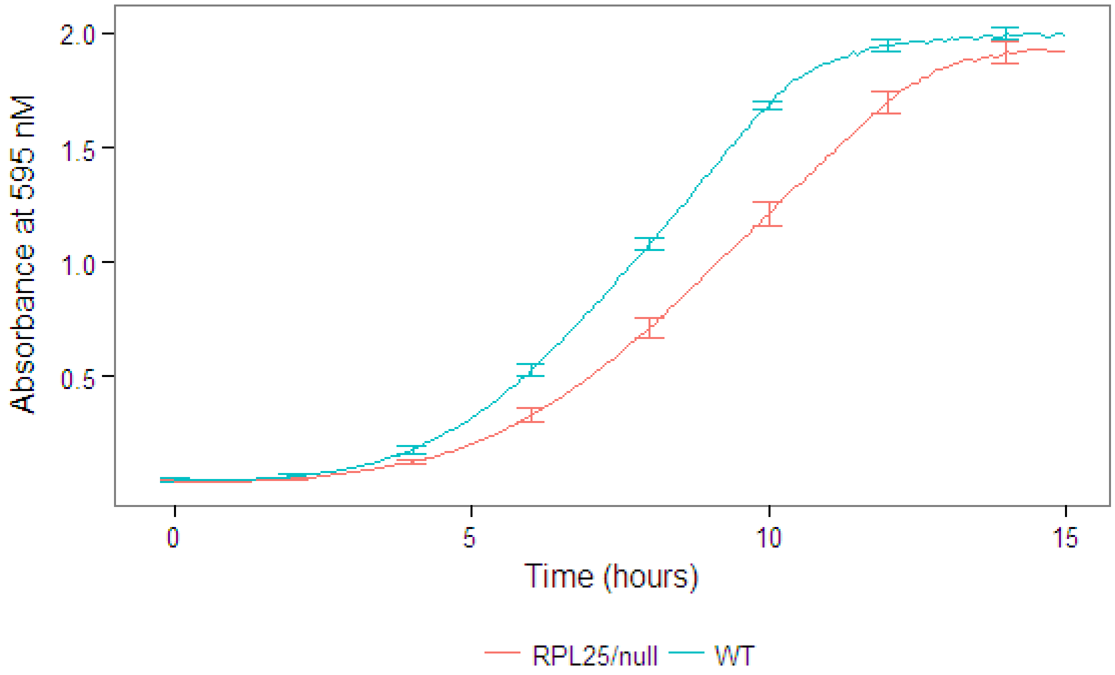

Figure S4 Growth curves for all strains found to be significantly unfit in our study. The error bars represent the 95% confidence interval under a Student's *t* distribution. The curve for the hemizygous strain is indicated by the gene name followed by "/null", alongside the wild type strain ("WT"), for comparison. (A1-6) Candidate genes in rich (YPD) media. (B1-5) Candidate genes in nitrogen-limited media. (C) A significantly unfit candidate gene in carbon-limited media. (D) A significantly unfit background control gene in rich media. (E1-2) Positive control genes in rich media.

Files S1-S2

Available for download at http://www.g3journal.org/lookup/suppl/doi:10.1534/g3.113.008144/-/DC1

File S1

Supplementary Dataset 1

The “Legend” sheet shows a legend for the gene properties that are represented as columns of the table in the “Gene Properties” sheet. In the sheet “Gene Properties,” each row is a single gene, and values for gene properties are shown across all genes considered in this work. “NA” denotes cases where gene property values are missing. The details and sources of the gene properties are described in detail in the text.

File S2

Supplementary Dataset 2

The “Legend” sheet shows a legend for the tables that appear in the sheets labeled “All Genes” and “Tested Genes.” In both sheets, the posterior probabilities of HI come from cross validation runs produced using the chosen “best” 6GP LDA model, which is described in detail in the text. The sheet “All Genes” shows posterior probabilities for all genes analysed, while the sheet “Tested Genes” shows these probabilities only for the genes tested experimentally in this work.

Table S1 Table describing three letter abbreviations, descriptions and data sources for gene properties shown in Figure S4.

Abbreviation	Description	Source of data
gdg	Genetic interaction network degree	Generated from data in the DryGIN database (Koh et al., 2009)
mxv	mRNA expression variation through the yeast cell cycle	Microarray data (Spellman et al., 1998)
odi	ORF DNA percentage between *S. cerevisiae* and *S. kudriavzevii*	Generated from cDNA gene sequences of two yeast organisms
pdg	Protein interaction network degree	Generated from data in the BioGRID database (Stark et. al., 2005)
pdi	Promoter sequence DNA identity between *S. cerevisiae* and *S. paradoxus*	Generated from intergenic sequences of two yeast organisms
psi	Summed intensities, representing combined haploid and diploid protein abundance.	Proteomics data (de Godoy et al., 2008)

Table S2 Table showing phenotypes of hemizygous strains experimentally tested in this work. The first column shows the strain tested. The second column indicates the experiment type, with “Candidate gene” denoting a gene that was selected according to its high HI probability, “Negative control” indicating a gene that was selected according to its low HI probability, “Background control” denoting a gene that was selected randomly, and “Positive control” indicating a gene selected because it was found to be HI in earlier work. The third column describes the type of media the strain was tested in. The fourth column shows the mutant AUGC relative to the WT AUGC, and the fifth shows the p-value describing the significance of the difference between the mutant and WT AUGCs. Rows highlighted green indicate strains that were found to be significantly HI, i.e. those with *p*-value < 0.05.

Strain	Experiment type	Media	AUGC mutant / AUGC WT	*p*-value
BEM2/null	Candidate gene	YPD	1.011	0.315
ASC1/null	Candidate gene	YPD	1.017	0.627
RPL16A/null	Candidate gene	YPD	1.007	0.590
RPL8B/null	Candidate gene	YPD	0.999	0.952
UBI4/null	Candidate gene	YPD	0.967	5.33×10^{-2}
RPL8A/null	Candidate gene	YPD	1.009	0.698
UBP3/null	Candidate gene	YPD	1.003	0.899
EFT2/null	Candidate gene	YPD	1.004	0.794
RPS6B/null	Candidate gene	YPD	0.908	8.31×10^{-4}
BCY1/null	Candidate gene	YPD	0.903	3.87×10^{-5}
BRE5/null	Candidate gene	YPD	0.947	1.48×10^{-2}
RPS19B/null	Candidate gene	YPD	0.998	0.899
RPL3/null	Candidate gene	YPD	0.996	0.821
ADH1/null	Candidate gene	YPD	0.998	0.922
GCN5/null	Candidate gene	YPD	0.966	3.47×10^{-2}
ERG6/null	Candidate gene	YPD	0.990	0.627
SEC28/null	Candidate gene	YPD	0.994	0.698
RPL28/null	Candidate gene	YPD	0.994	0.718
HOM6/null	Candidate gene	YPD	1.035	0.510×10^{-2}
HMO1/null	Candidate gene	YPD	0.994	0.846
PHO23/null	Candidate gene	YPD	1.036	0.118
RPL10/null	Candidate gene	YPD	0.942	1.48×10^{-2}
RPN10/null	Candidate gene	YPD	0.961	1.48×10^{-2}
BEM2/null	Candidate gene	F1 nitrogen-limited	0.987	0.462
ASC1/null	Candidate gene	F1 nitrogen-limited	1.008	0.445
RPL16A/null	Candidate gene	F1 nitrogen-limited	0.999	0.962
RPL8B/null	Candidate gene	F1 nitrogen-limited	0.995	0.445
UBI4/null	Candidate gene	F1 nitrogen-limited	0.996	0.682
RPL8A/null	Candidate gene	F1 nitrogen-limited	1.008	0.431
UBP3/null	Candidate gene	F1 nitrogen-limited	1.009	0.642
EFT2/null	Candidate gene	F1 nitrogen-limited	1.019	0.104
RPS6B/null	Candidate gene	F1 nitrogen-limited	0.965	1.35×10^{-4}
BCY1/null	Candidate gene	F1 nitrogen-limited	0.925	5.39×10^{-5}
BRE5/null	Candidate gene	F1 nitrogen-limited	0.956	1.00×10^{-3}
RPS19B/null	Candidate gene	F1 nitrogen-limited	0.991	0.404
RPL3/null	Candidate gene	F1 nitrogen-limited	0.973	0.158
ADH1/null	Candidate gene	F1 nitrogen-limited	0.993	0.445
GCN5/null	Candidate gene	F1 nitrogen-limited	0.982	0.445
ERG6/null	Candidate gene	F1 nitrogen-limited	1.003	0.720
SEC28/null	Candidate gene	F1 nitrogen-limited	0.948	3.33×10^{-4}
RPL28/null	Candidate gene	F1 nitrogen-limited	1.001	0.992
HOM6/null	Candidate gene	F1 nitrogen-limited	1.003	0.791
HMO1/null	Candidate gene	F1 nitrogen-limited	0.970	3.47×10^{-2}
PHO23/null	Candidate gene	F1 nitrogen-limited	0.986	0.158

RPL10/null	Candidate gene	F1 nitrogen-limited	0.986	0.104
RPN10/null	Candidate gene	F1 nitrogen-limited	0.994	0.431
BEM2/null	Candidate gene	F1 carbon-limited	0.972	0.544
ASC1/null	Candidate gene	F1 carbon-limited	0.972	0.242
RPL16A/null	Candidate gene	F1 carbon-limited	1.002	0.996
RPL8B/null	Candidate gene	F1 carbon-limited	1.007	0.976
UBI4/null	Candidate gene	F1 carbon-limited	1.015	0.841
RPL8A/null	Candidate gene	F1 carbon-limited	1.057	6.82×10^{-2}
UBP3/null	Candidate gene	F1 carbon-limited	1.003	0.976
EFT2/null	Candidate gene	F1 carbon-limited	1.027	0.544
RPS6B/null	Candidate gene	F1 carbon-limited	1.000	0.996
BCY1/null	Candidate gene	F1 carbon-limited	0.874	3.52×10^{-4}
BRE5/null	Candidate gene	F1 carbon-limited	0.963	0.107
RPS19B/null	Candidate gene	F1 carbon-limited	0.959	0.159
RPL3/null	Candidate gene	F1 carbon-limited	0.978	0.611
ADH1/null	Candidate gene	F1 carbon-limited	0.998	0.976
GCN5/null	Candidate gene	F1 carbon-limited	0.982	0.752
ERG6/null	Candidate gene	F1 carbon-limited	1.016	0.840
SEC28/null	Candidate gene	F1 carbon-limited	0.990	0.824
RPL28/null	Candidate gene	F1 carbon-limited	1.000	0.996
HOM6/null	Candidate gene	F1 carbon-limited	0.995	0.958
HMO1/null	Candidate gene	F1 carbon-limited	0.991	0.841
PHO23/null	Candidate gene	F1 carbon-limited	0.989	0.824
RPL10/null	Candidate gene	F1 carbon-limited	0.990	0.841
RPN10/null	Candidate gene	F1 carbon-limited	1.002	0.996
UPF3/null	Negative control	YPD	0.998	0.899
VAN1/null	Negative control	YPD	1.011	0.459
PUS1/null	Negative control	YPD	1.004	0.899
CFD1/null	Negative control	YPD	1.021	0.529
TRM82/null	Negative control	YPD	1.024	0.459
ATM1/null	Negative control	YPD	1.018	0.251
FET4/null	Negative control	YPD	1.035	5.33×10^{-2}
SLG1/null	Negative control	YPD	0.991	0.529
PRM10/null	Negative control	YPD	1.014	0.435
MPD2/null	Negative control	YPD	0.993	0.633
TMA64/null	Negative control	YPD	1.024	0.155
GPM3/null	Negative control	YPD	1.008	0.698
RBG2/null	Negative control	YPD	1.028	0.132
SEN54/null	Negative control	YPD	1.013	0.426
PAN6/null	Negative control	YPD	1.012	0.590
YEL1/null	Negative control	YPD	1.027	0.140
CNN1/null	Negative control	YPD	1.022	0.399
AIM36/null	Negative control	YPD	1.021	0.251
COS10/null	Negative control	YPD	0.990	0.719
ICS3/null	Negative control	YPD	1.002	0.952
PLB3/null	Negative control	YPD	1.000	0.972
BNA4/null	Negative control	YPD	0.991	0.633
FRE2/null	Negative control	YPD	1.004	0.719
CTS2/null	Negative control	YPD	1.009	0.633
SSD1/null	Background control	YPD	1.016	0.577
ZUO1/null	Background control	YPD	0.974	0.210
SFA1/null	Background control	YPD	1.011	0.595
CDC60/null	Background control	YPD	1.004	0.890
ATP11/null	Background control	YPD	1.023	0.324

RVB1/null	Background control	YPD	1.007	0.785
UBP15/null	Background control	YPD	0.995	0.770
ATP4/null	Background control	YPD	1.011	0.785
YAR1/null	Background control	YPD	0.997	0.886
YFH1/null	Background control	YPD	0.985	0.376
ARC40/null	Background control	YPD	1.028	0.210
MRP51/null	Background control	YPD	0.992	0.785
RSC9/null	Background control	YPD	0.969	0.157
APS3/null	Background control	YPD	0.987	0.605
REX4/null	Background control	YPD	1.010	0.715
KRE11/null	Background control	YPD	1.014	0.376
TAL1/null	Background control	YPD	0.996	0.881
IOC3/null	Background control	YPD	1.007	0.785
MIH1/null	Background control	YPD	1.009	0.657
CTA1/null	Background control	YPD	1.021	0.376
BUB1/null	Background control	YPD	0.871	2.35×10^{-5}
PCP1/null	Background control	YPD	0.999	0.940
YMD8/null	Background control	YPD	1.003	0.905
MRM2/null	Background control	YPD	1.016	0.553
ARK1/null	Background control	YPD	1.001	0.964
NSE3/null	Background control	YPD	1.000	0.964
NSG1/null	Background control	YPD	1.014	0.595
RGT1/null	Background control	YPD	0.996	0.863
VCX1/null	Background control	YPD	0.983	0.376
SMK1/null	Background control	YPD	0.979	0.376
AIM33/null	Background control	YPD	1.002	0.890
TVP38/null	Background control	YPD	0.996	0.858
ACF2/null	Background control	YPD	0.996	0.863
AAT1/null	Background control	YPD	1.020	0.577
ETP1/null	Background control	YPD	1.005	0.863
ALP1/null	Background control	YPD	1.027	6.24×10^{-2}
AIM32/null	Background control	YPD	1.007	0.657
HOR7/null	Background control	YPD	1.005	0.785
GAP1/null	Background control	YPD	1.002	0.905
PUG1/null	Background control	YPD	1.013	0.577
RTT105/null	Background control	YPD	1.028	0.210
THI20/null	Background control	YPD	0.971	0.210
RPL25/null	Positive control	YPD	0.810	1.84×10^{-5}
RPN11/null	Positive control	YPD	0.974	0.190
TUB1/null	Positive control	YPD	0.841	1.29×10^{-6}
