# Supplementary material for: Characterization and Prediction of Haploinsufficiency Using Systems-Level Gene Properties in Yeast
Source: G3 (Bethesda). 2013 Nov 1;3(11):1965–77. doi: 10.1534/g3.113.008144 (PMC3815059; doi:10.1534/g3.113.008144)
Supplement: Supporting Information [file supp_g3.113.008144_FigureS1.pdf]

(A) Rich medium (YPD), from Deutschbauer et al. (2005).

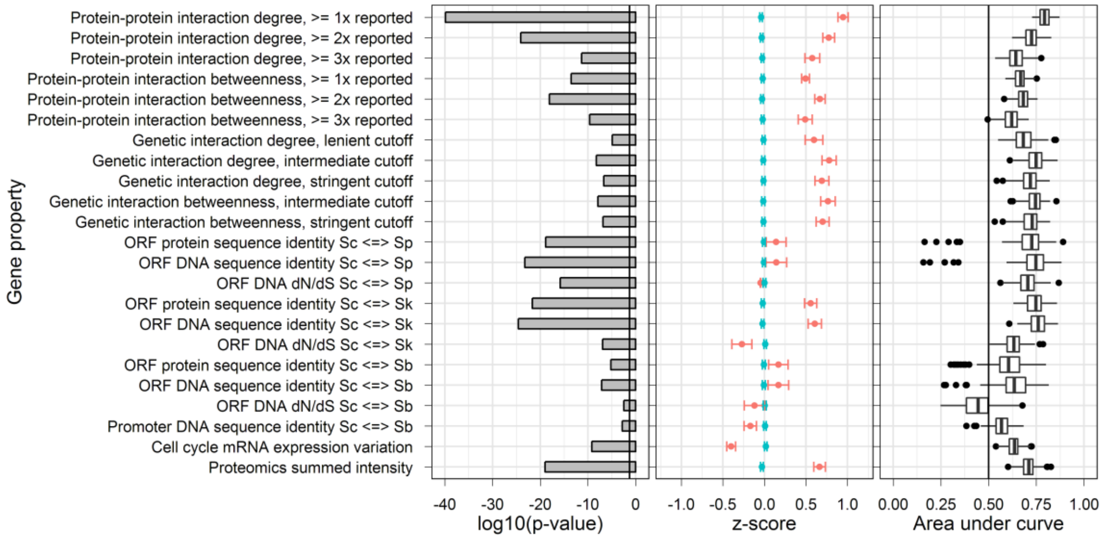

(B) Minimal medium (MM), from Deutschbauer et al. (2005).

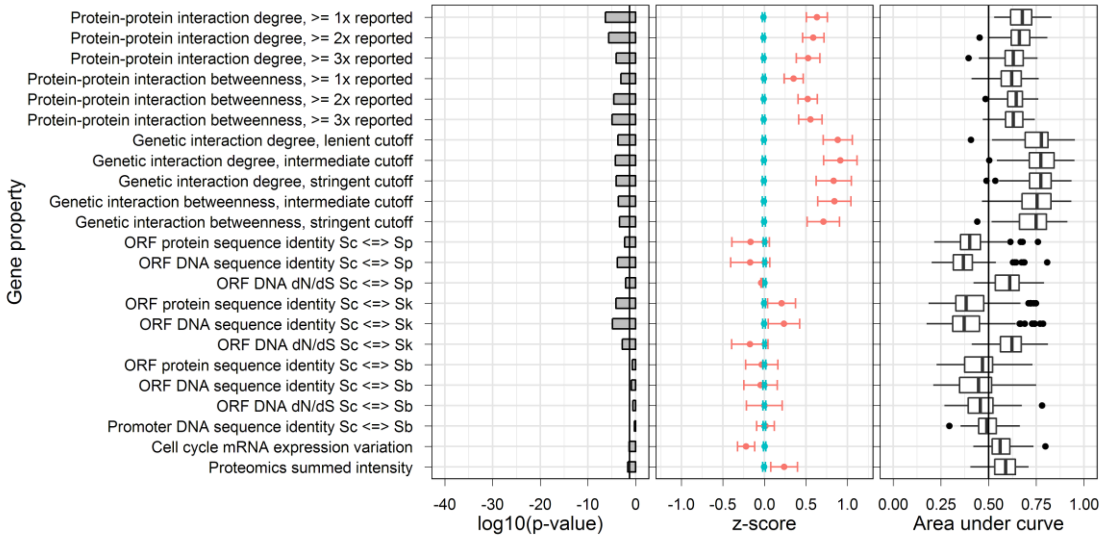

(C) F1 medium with carbon limitation. From Delneri et al. (2008).

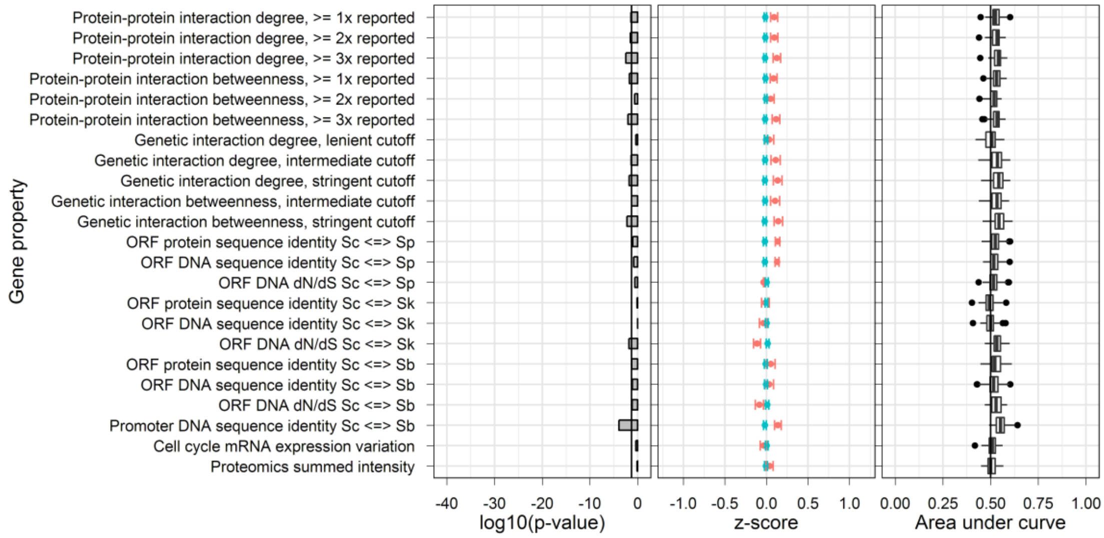

(D) F1 medium with nitrogen limitation. From Delneri et al. (2008).

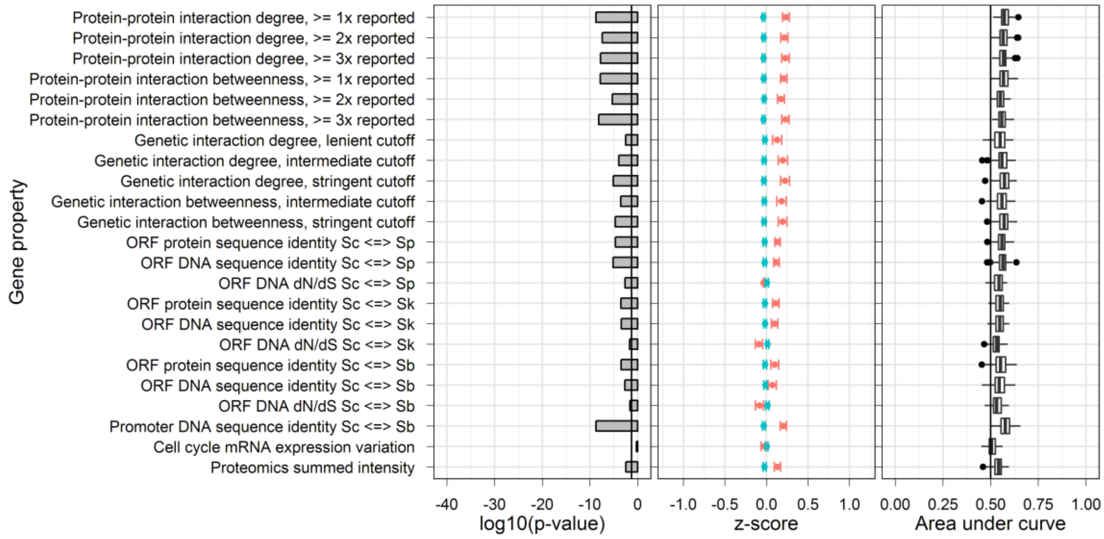

(E) F1 medium with phosphate limitation. From Delneri et al. (2008).

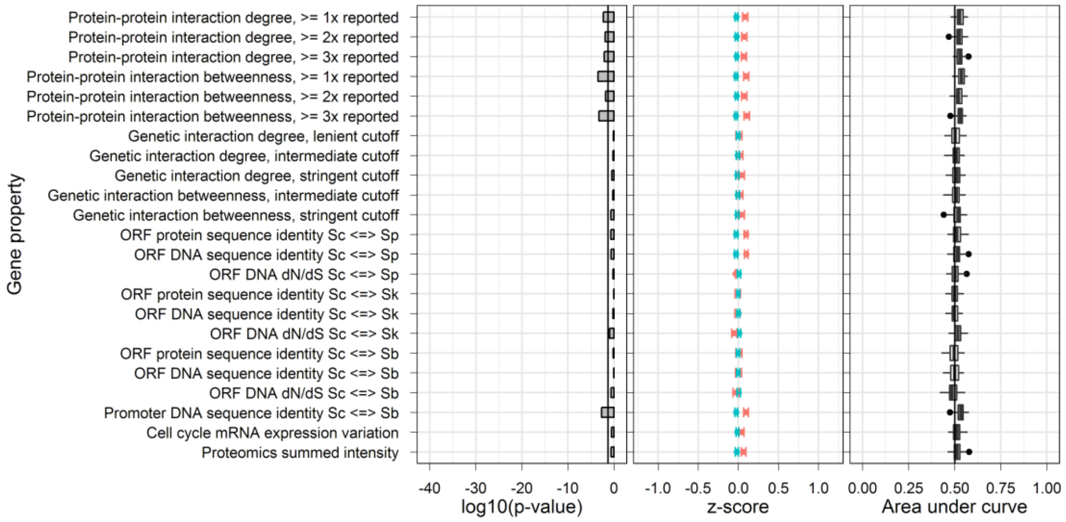

(F) White grape juice. From Delneri et al. (2008).

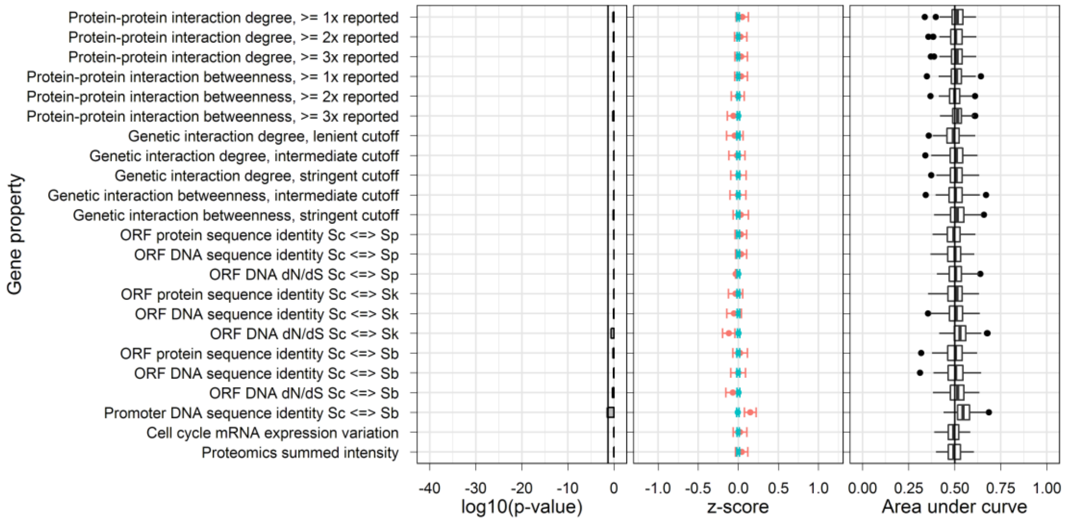

Figure S1 Relationships between HI and non-HI gene properties across 6 environments. Environments include (A) rich medium, (B) minimal medium, (C) F1 medium with carbon limitation, (D) F1 medium with nitrogen limitation, (E) F1 medium with phosphate limitation, (F) white grape juice. Left panel: Distribution differences are tested using *p*-values on a log10 scale, as estimated by the Mann-Whitley U test. The vertical line shows a *p*-value of 0.05. Center panel: Mean *z*-scores of HI (red) and non-HI (blue) gene properties are shown. Error bars represent the standard error of the mean. Right panel: ROC curve AUC distributions. These were generated using cross validation (see Methods). Whiskers represent lowest point within 1.5 interquartile range (IQR) of the lower quartile, and highest point within 1.5 IQR of the upper quartile. Dots represent outliers of the aforementioned ranges. The vertical line in the center of the chart represents the random expectation for the ROC plot.
