# Supplementary material for: Characterization and Prediction of Haploinsufficiency Using Systems-Level Gene Properties in Yeast
Source: G3 (Bethesda). 2013 Nov 1;3(11):1965–77. doi: 10.1534/g3.113.008144 (PMC3815059; doi:10.1534/g3.113.008144)
Supplement: Supporting Information [file supp_g3.113.008144_FigureS2.pdf]

	Protein abundance	Cell cycle mRNA variation	Promoter sequence identity	ORF DNA sequence identity	GI network betweenness	GI degree	PPI network betweenness	PPI network degree
Protein abundance								
Cell cycle mRNA variation	-0.1							
Promoter sequence identity	0.05	0.06						
ORF DNA sequence identity	0.14	-0.1	-					
GI network betweenness	0.06	-0.1	0.06	0.11				
GI degree	0.07	-0.1	0.06	0.12	0.98			
PPI network betweenness	0.26	-	0.04	0.11	0.24	0.27		
PPI network degree	0.34	-	0.05	0.15	0.24	0.26	0.7	

Figure S2 Pearson's product-moment correlation coefficients between 8 gene properties. "-" denotes cases where no correlation was detected, i.e. a p value > 0.05.
