# Supplementary material for: Characterization and Prediction of Haploinsufficiency Using Systems-Level Gene Properties in Yeast
Source: G3 (Bethesda). 2013 Nov 1;3(11):1965–77. doi: 10.1534/g3.113.008144 (PMC3815059; doi:10.1534/g3.113.008144)
Supplement: Supporting Information [file supp_g3.113.008144_FigureS3.pdf]

(A) Roll back to a simpler model.

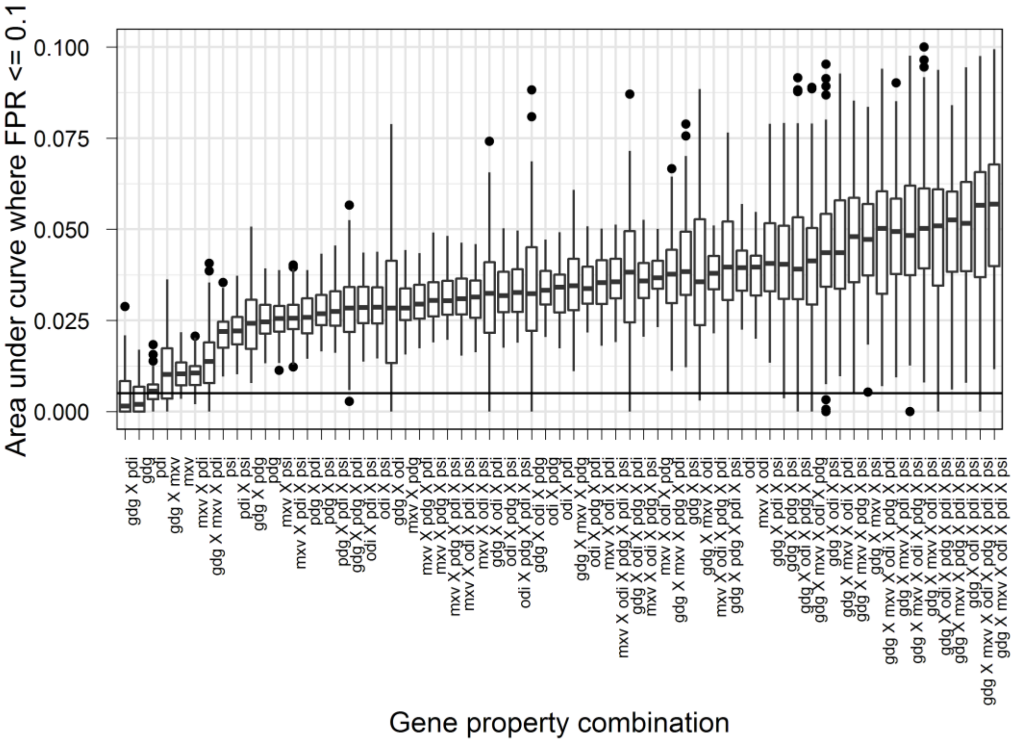

(B) Exclude incomplete cases.

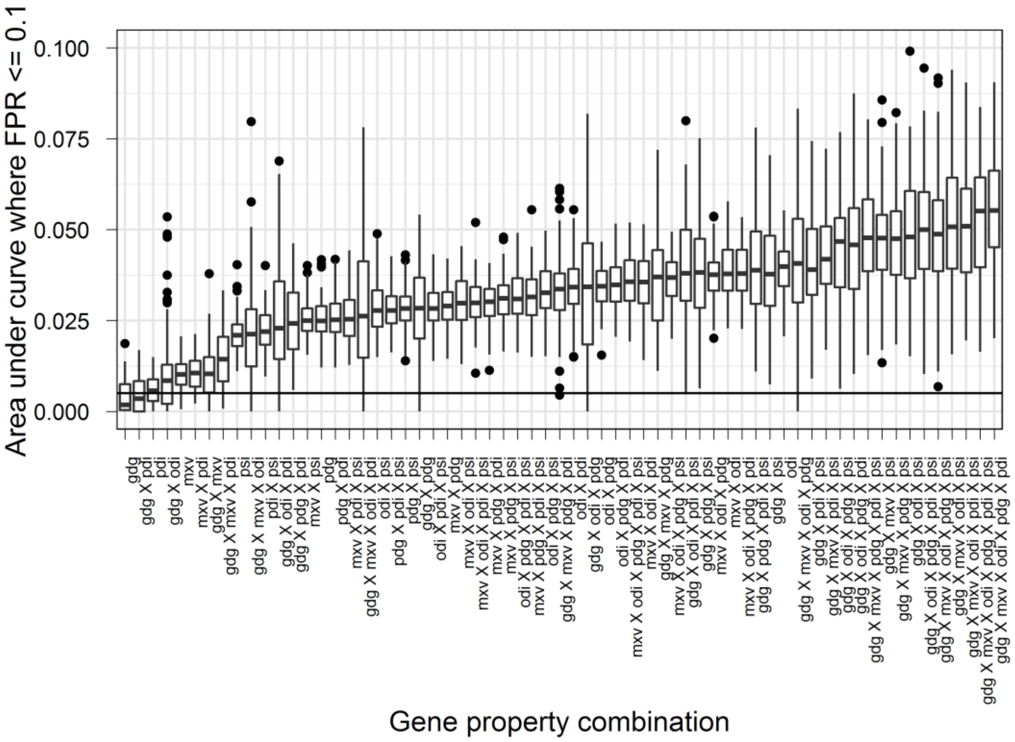

(C) Expectation-maximisation algorithm.

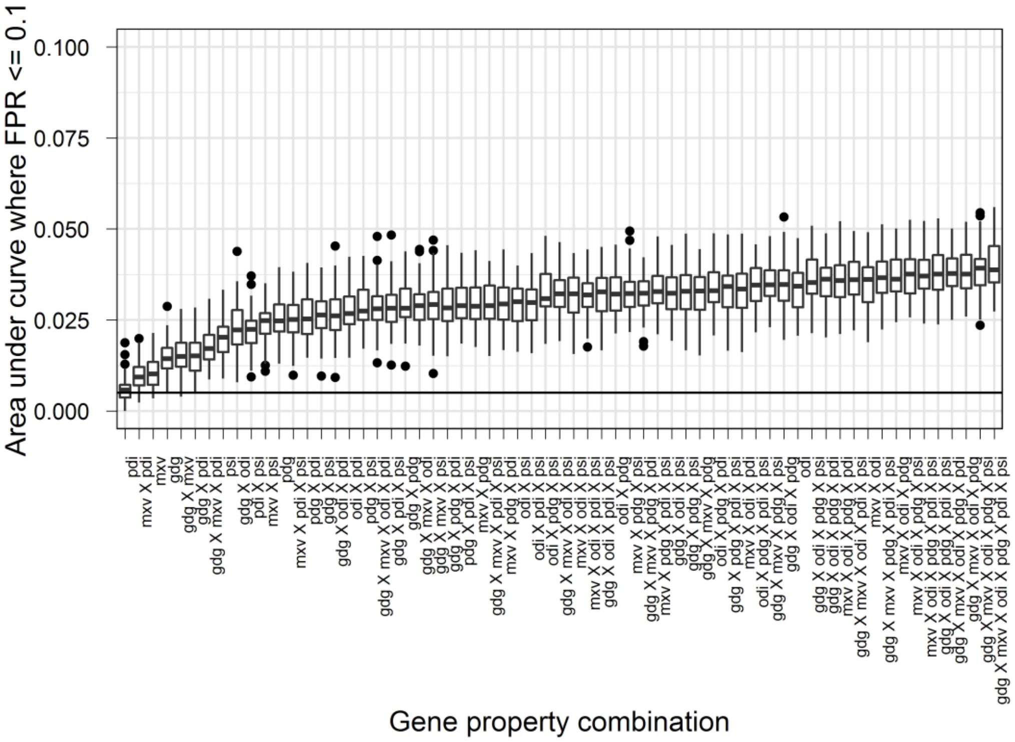

(D) Multiple imputation.

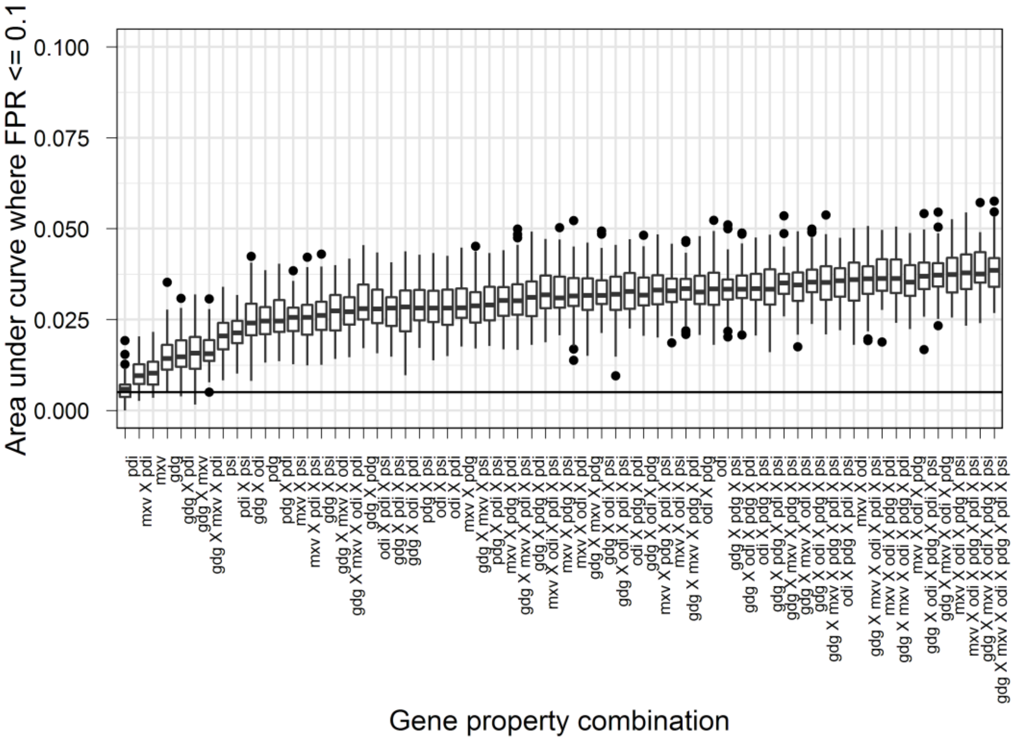

(E) Median imputation.

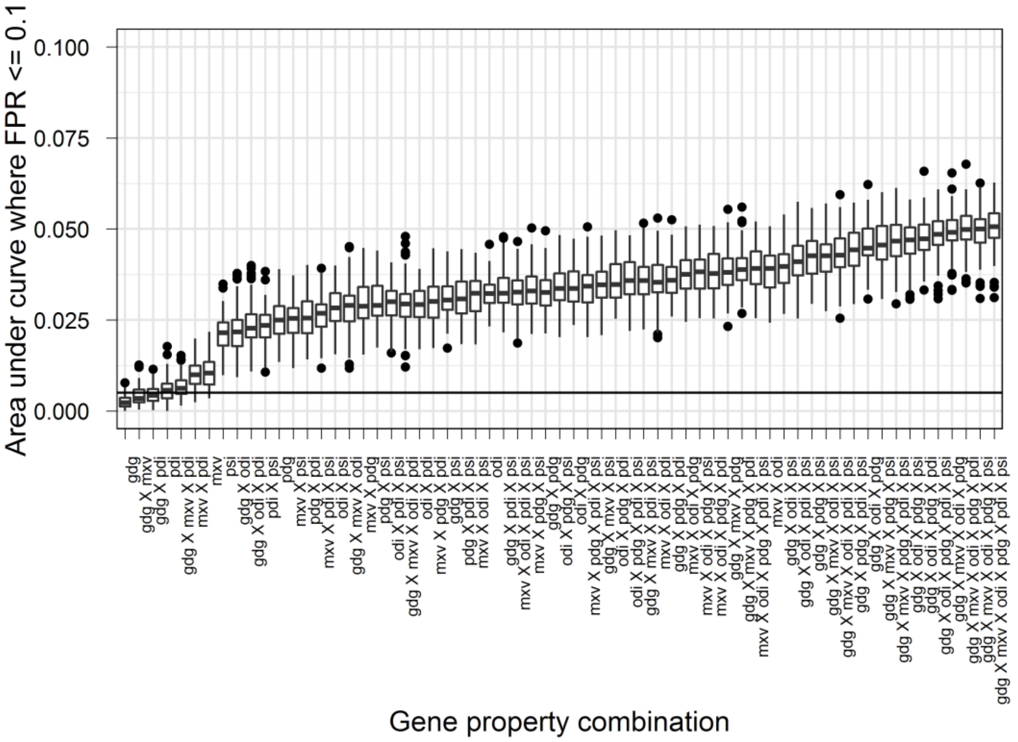

Abbreviation	Description
gdg	Genetic interaction network degree
mxv	mRNA expression variation through the yeast cell cycle
odi	ORF DNA percentage between *S. cerevisiae* and *S. kudriavzevii*
pdg	Protein interaction network degree
pdi	Promoter sequence DNA identity between *S. cerevisiae* and *S. paradoxus*
psi	Summed intensities, representing combined haploid and diploid protein abundance.

Figure S3 FPR < 0.1 AUC distribution across all combinations of gene properties, using 5 missing value handling methods. Methods for missing value treatment include (A) rolling back to a simpler model, (B) excluding incomplete cases, (C) the expectation-maximisation algorithm, (D) multiple imputation, and (E) median imputation. The three letter codes identify gene properties and are described in the legend. Distributions are for 100 ROC curves generated during cross validation (see Methods). Whiskers represent lowest point within 1.5 interquartile range (IQR) of the lower quartile, and highest point within 1.5 IQR of the upper quartile. Dots represent outliers of the aforementioned ranges. The black horizontal line represents the random expectation from the ROC plot.
