# Supplementary material for: Characterization and Prediction of Haploinsufficiency Using Systems-Level Gene Properties in Yeast
Source: G3 (Bethesda). 2013 Nov 1;3(11):1965–77. doi: 10.1534/g3.113.008144 (PMC3815059; doi:10.1534/g3.113.008144)
Supplement: Supporting Information [file supp_g3.113.008144_FigureS4.pdf]

__A__ - Candidate genes in rich medium.

(A1)

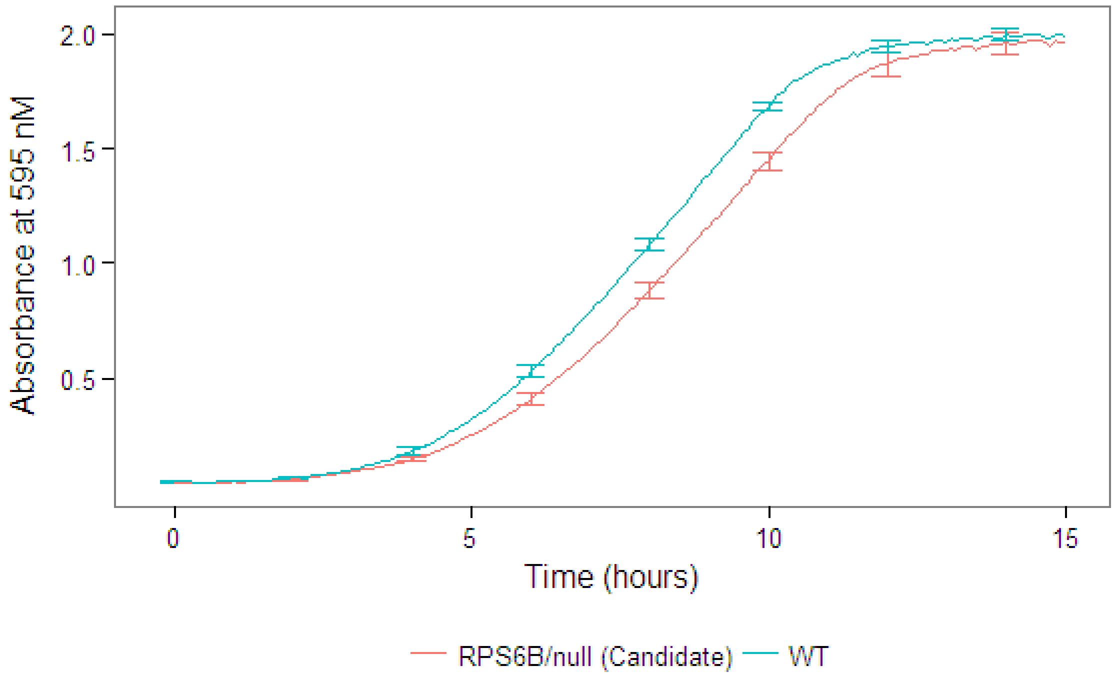

(A2)

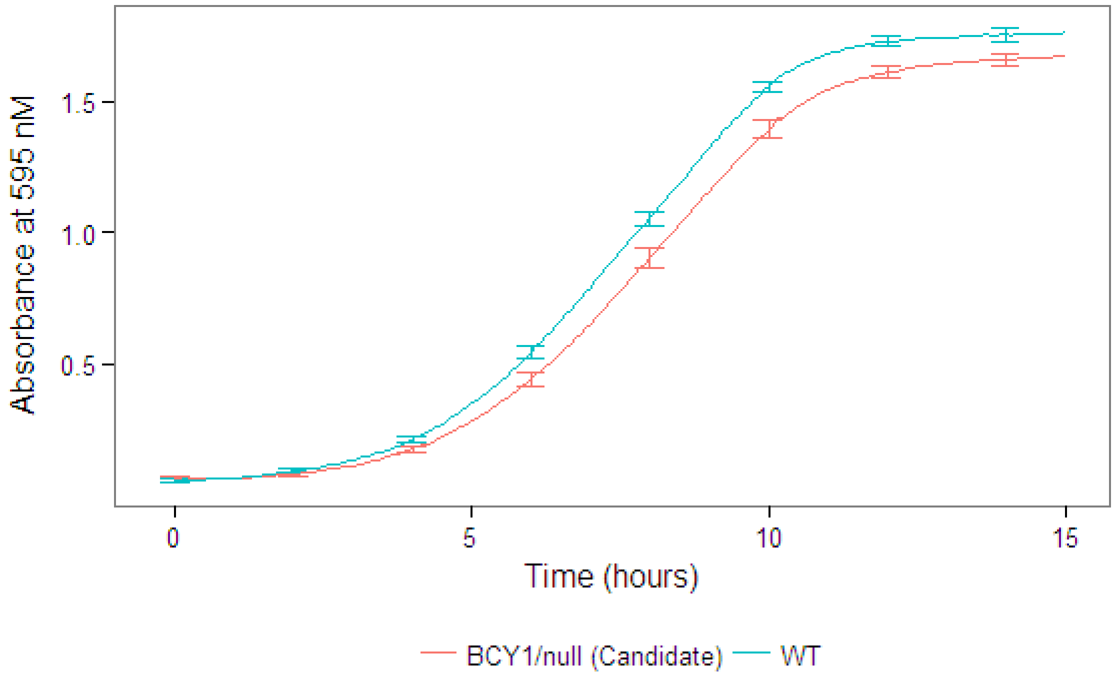

(A3)

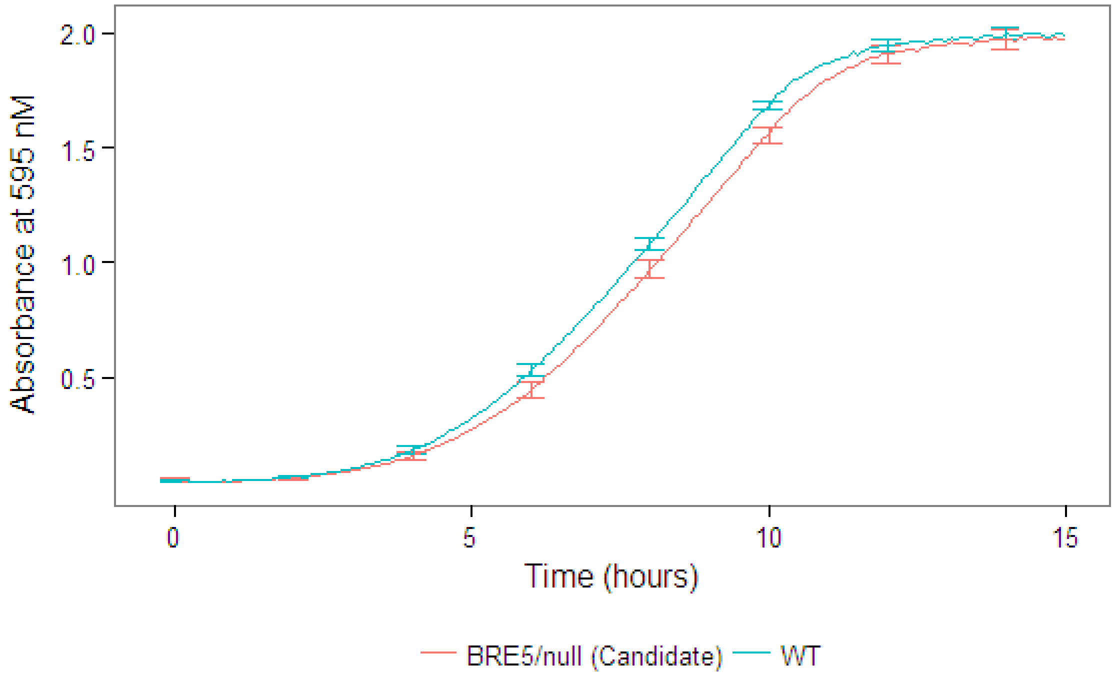

2.0
1.5
1.0
0.5
Absorbance at 595 nM
0
5
10
15
Time (hours)
BRE5/null (Candidate)
WT

(A4)

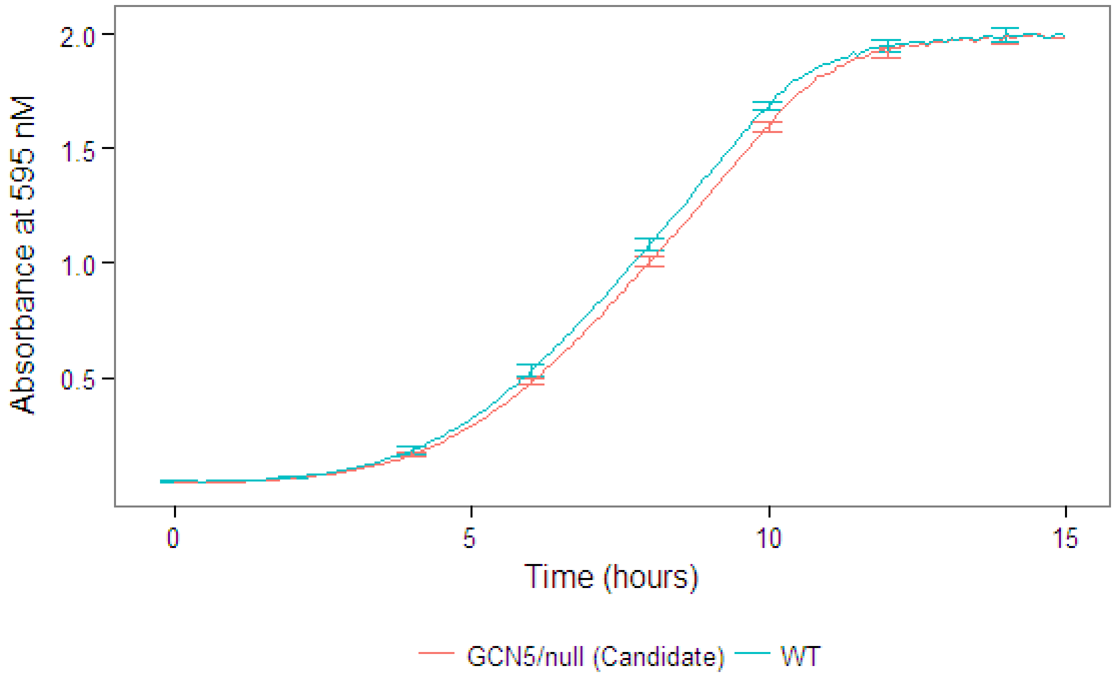

2.0
1.5
1.0
0.5
Absorbance at 595 nM
0
5
10
15
Time (hours)
GCN5/null (Candidate)
WT

(A5)

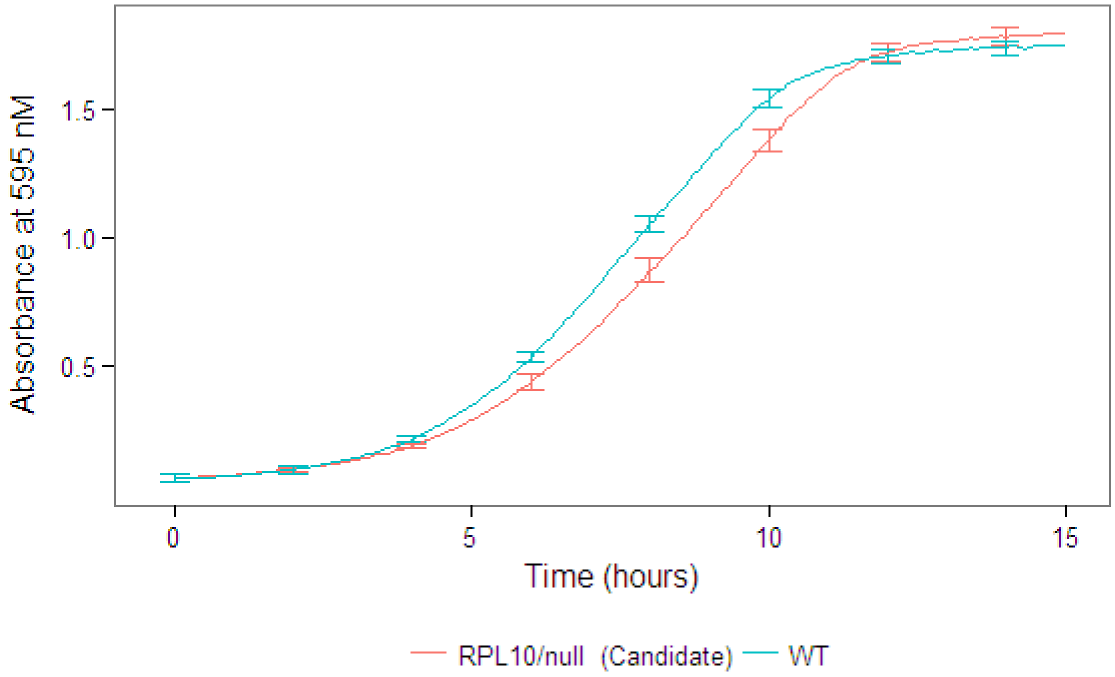
Absorbance at 595 nM
1.5
1.0
0.5
0
5
10
15
Time (hours)
RPL10/null (Candidate)
WT

(A6)

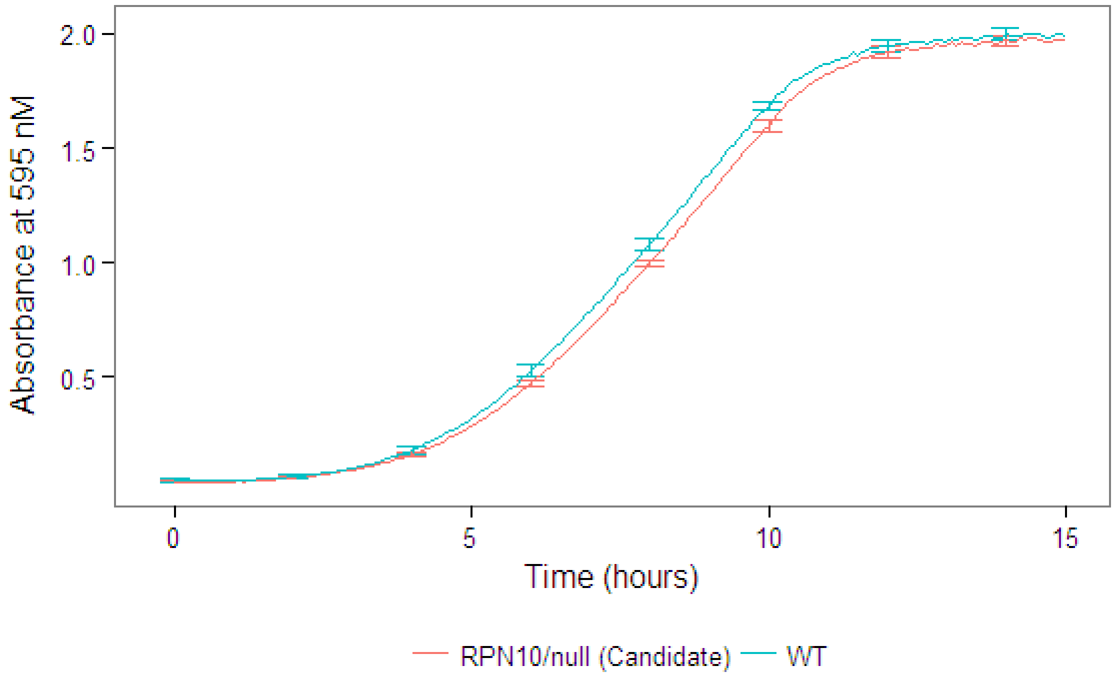
Absorbance at 595 nM
2.0
1.5
1.0
0.5
0
5
10
15
Time (hours)
RPN10/null (Candidate)
WT

***B –** Candidate genes in nitrogen-limited medium.*

(B1)

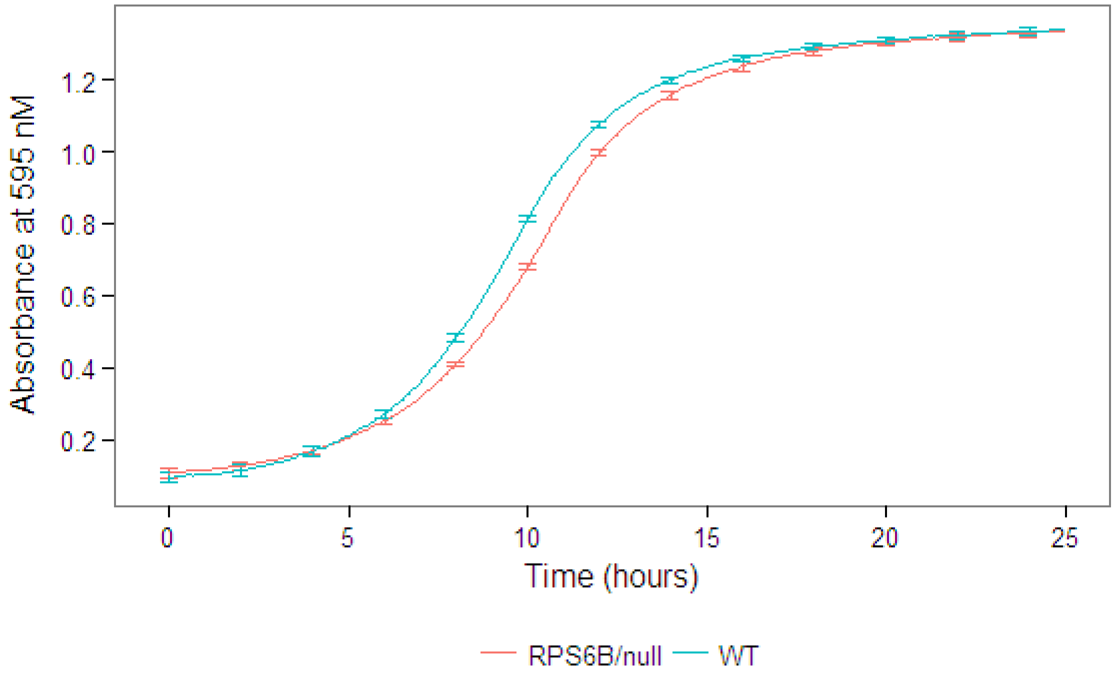

(B2)

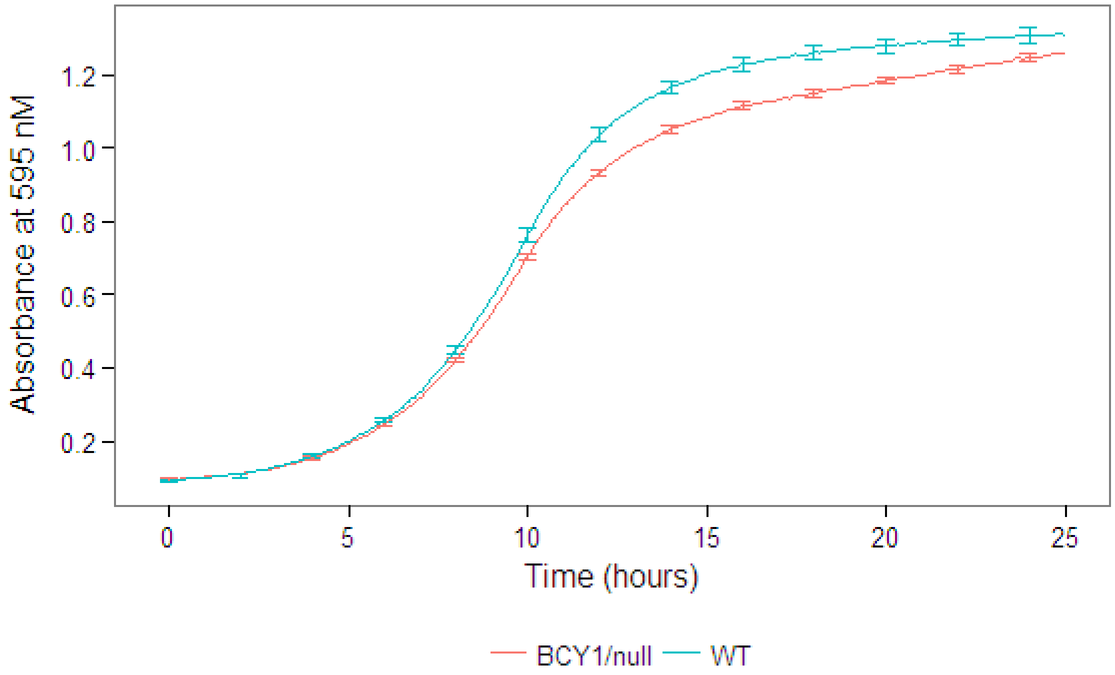

(B3)

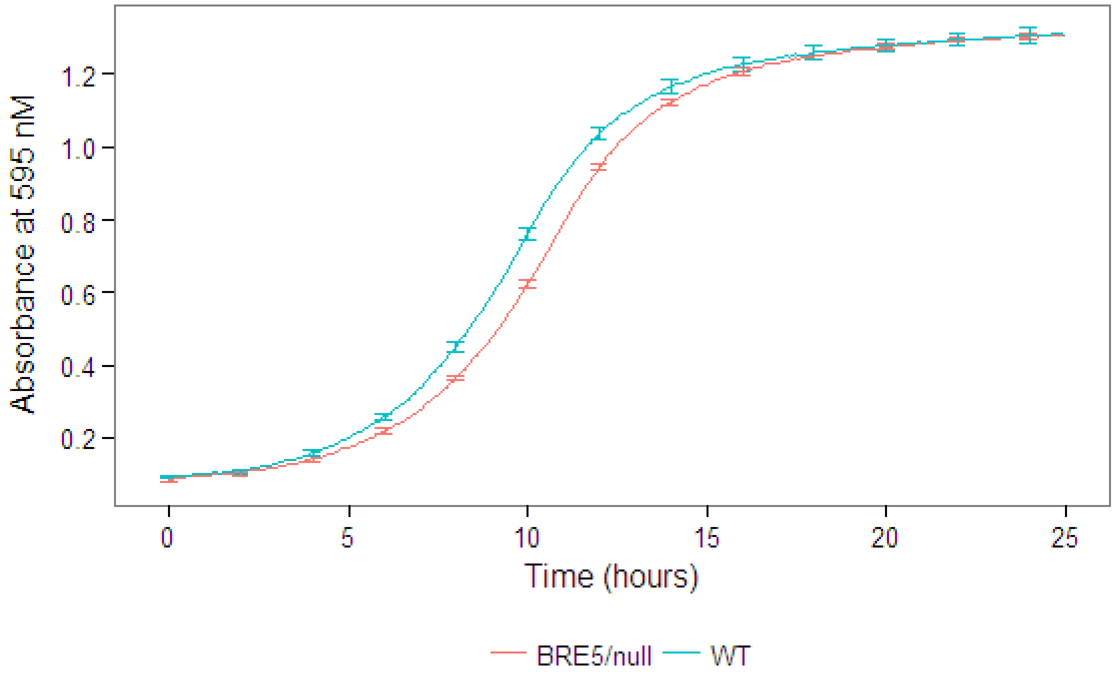
1.2
1.0
0.8
0.6
0.4
0.2
Absorbance at 595 nM
0
5
10
15
20
25
Time (hours)
BRE5/null
WT

(B4)

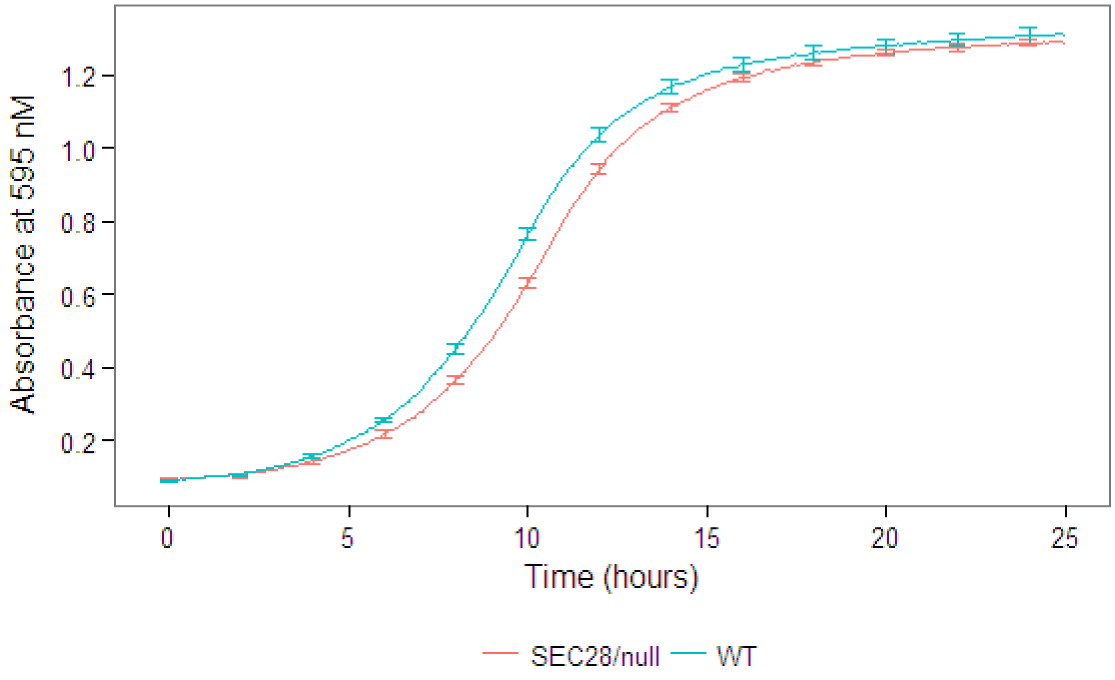
1.2
1.0
0.8
0.6
0.4
0.2
Absorbance at 595 nM
0
5
10
15
20
25
Time (hours)
SEC28/null
WT

(B5)

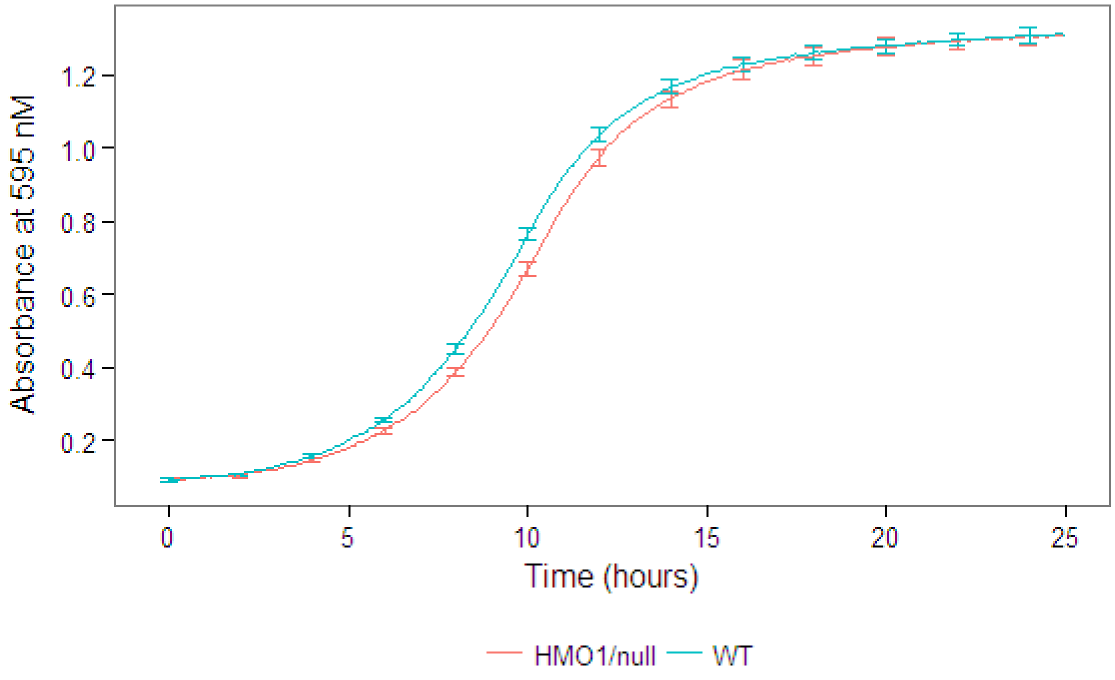

***C** – Candidate gene in carbon-limited media.*

(C)

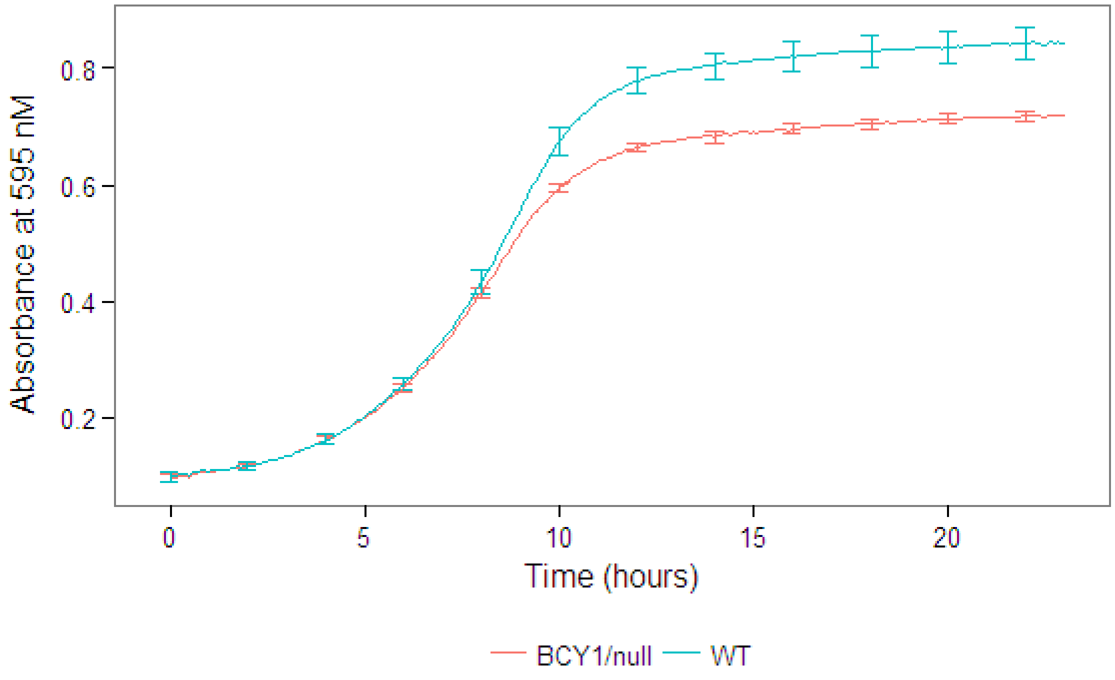

D – Background control gene in rich medium.

(D)

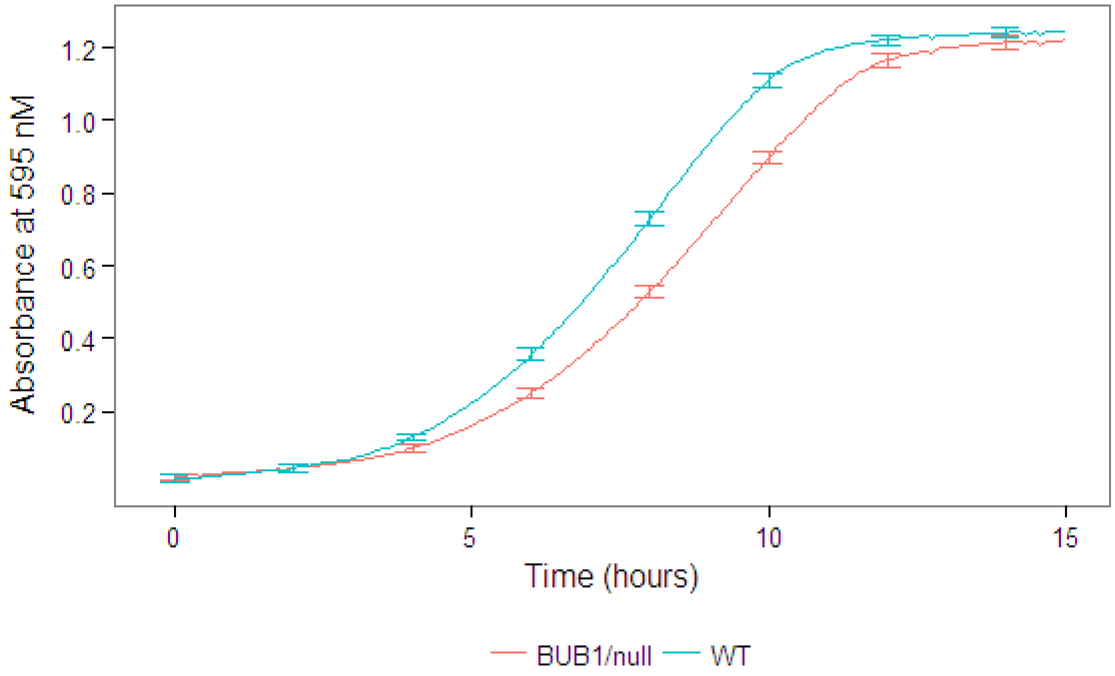

***E** – Positive control genes in rich medium.*

(E1)

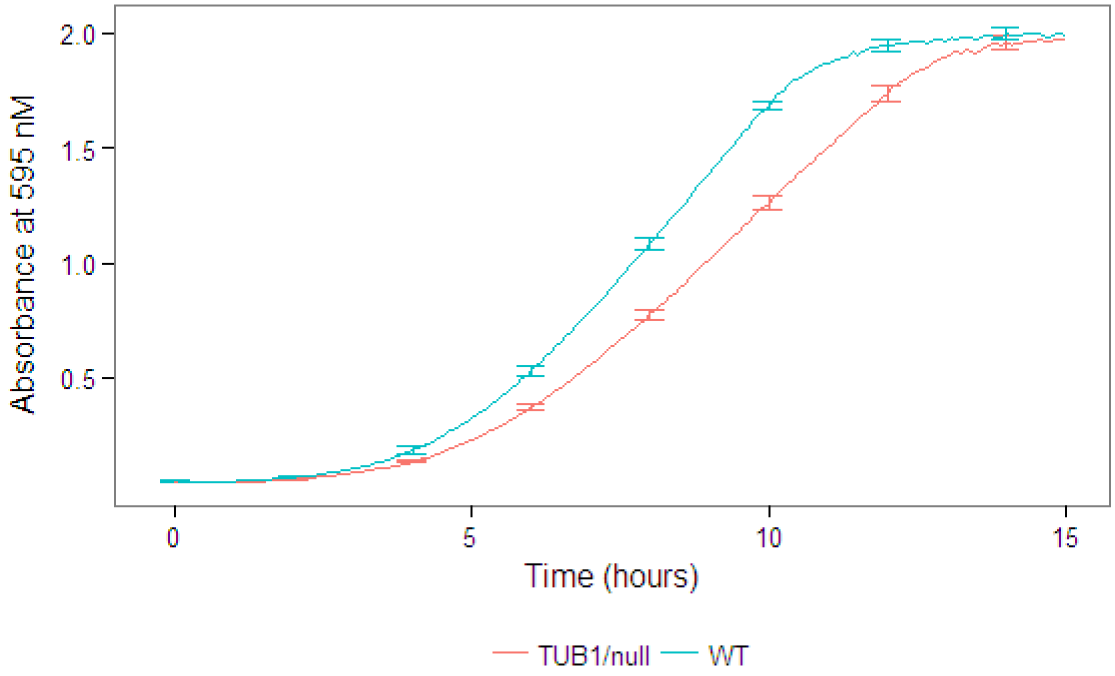

(E2)

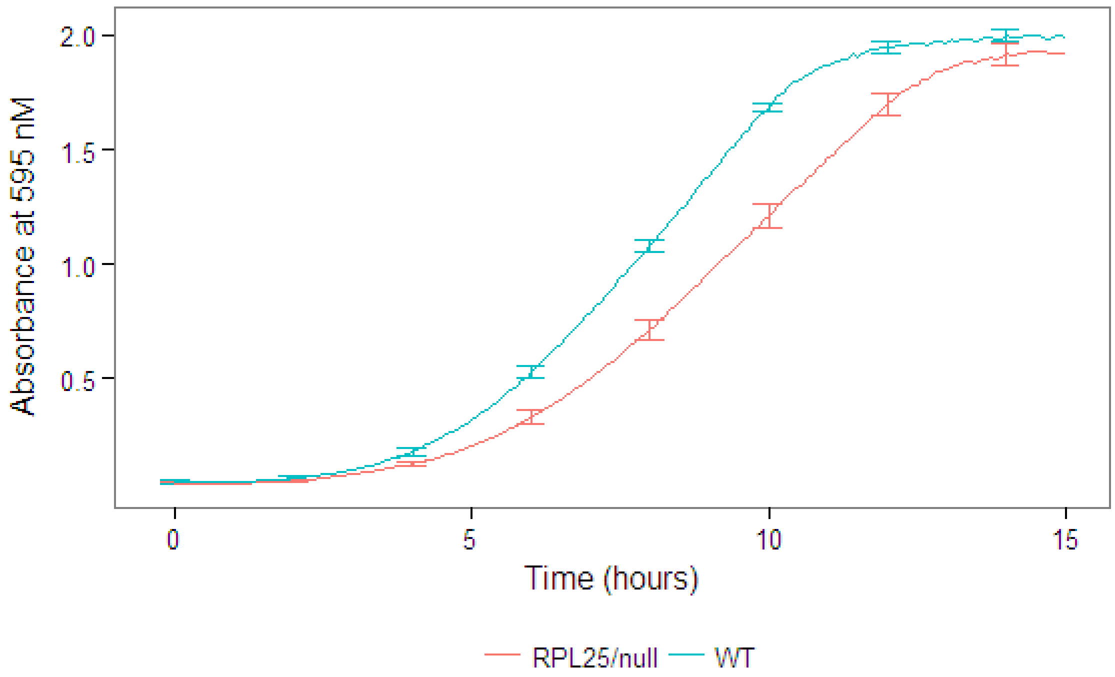

Figure S4 Growth curves for all strains found to be significantly unfit in our study. The error bars represent the 95% confidence interval under a Student's *t* distribution. The curve for the hemizygous strain is indicated by the gene name followed by "/null", alongside the wild type strain ("WT"), for comparison. (A1-6) Candidate genes in rich (YPD) media. (B1-5) Candidate genes in nitrogen-limited media. (C) A significantly unfit candidate gene in carbon-limited media. (D) A significantly unfit background control gene in rich media. (E1-2) Positive control genes in rich media.
