# Supplementary material for: Characterization and Prediction of Haploinsufficiency Using Systems-Level Gene Properties in Yeast
Source: G3 (Bethesda). 2013 Nov 1;3(11):1965–77. doi: 10.1534/g3.113.008144 (PMC3815059; doi:10.1534/g3.113.008144)
Supplement: Supporting Information [file supp_g3.113.008144_TableS1.pdf]

Table S1 Table describing three letter abbreviations, descriptions and data sources for gene properties shown in Figure S4.

Abbreviation	Description	Source of data
gdg	Genetic interaction network degree	Generated from data in the DryGIN database (Koh et al., 2009)
mxv	mRNA expression variation through the yeast cell cycle	Microarray data (Spellman et al., 1998)
odi	ORF DNA percentage between *S. cerevisiae* and *S. kudriavzevii*	Generated from cDNA gene sequences of two yeast organisms
pdg	Protein interaction network degree	Generated from data in the BioGRID database (Stark et. al., 2005)
pdi	Promoter sequence DNA identity between *S. cerevisiae* and *S. paradoxus*	Generated from intergenic sequences of two yeast organisms
psi	Summed intensities, representing combined haploid and diploid protein abundance.	Proteomics data (de Godoy et al., 2008)
