# Supplementary material for: Characterization and Prediction of Haploinsufficiency Using Systems-Level Gene Properties in Yeast
Source: G3 (Bethesda). 2013 Nov 1;3(11):1965–77. doi: 10.1534/g3.113.008144 (PMC3815059; doi:10.1534/g3.113.008144)
Supplement: Supporting Information [file supp_g3.113.008144_TableS2.pdf]

Table S2 Table showing phenotypes of hemizygous strains experimentally tested in this work. The first column shows the strain tested. The second column indicates the experiment type, with “Candidate gene” denoting a gene that was selected according to its high HI probability, “Negative control” indicating a gene that was selected according to its low HI probability, “Background control” denoting a gene that was selected randomly, and “Positive control” indicating a gene selected because it was found to be HI in earlier work. The third column describes the type of media the strain was tested in. The fourth column shows the mutant AUGC relative to the WT AUGC, and the fifth shows the p-value describing the significance of the difference between the mutant and WT AUGCs. Rows highlighted green indicate strains that were found to be significantly HI, i.e. those with *p*-value < 0.05.

Strain	Experiment type	Media	AUGC mutant / AUGC WT	*p*-value
BEM2/null	Candidate gene	YPD	1.011	0.315
ASC1/null	Candidate gene	YPD	1.017	0.627
RPL16A/null	Candidate gene	YPD	1.007	0.590
RPL8B/null	Candidate gene	YPD	0.999	0.952
UBI4/null	Candidate gene	YPD	0.967	5.33×10^{-2}
RPL8A/null	Candidate gene	YPD	1.009	0.698
UBP3/null	Candidate gene	YPD	1.003	0.899
EFT2/null	Candidate gene	YPD	1.004	0.794
RPS6B/null	Candidate gene	YPD	0.908	8.31×10^{-4}
BCY1/null	Candidate gene	YPD	0.903	3.87×10^{-5}
BRE5/null	Candidate gene	YPD	0.947	1.48×10^{-2}
RPS19B/null	Candidate gene	YPD	0.998	0.899
RPL3/null	Candidate gene	YPD	0.996	0.821
ADH1/null	Candidate gene	YPD	0.998	0.922
GCN5/null	Candidate gene	YPD	0.966	3.47×10^{-2}
ERG6/null	Candidate gene	YPD	0.990	0.627
SEC28/null	Candidate gene	YPD	0.994	0.698
RPL28/null	Candidate gene	YPD	0.994	0.718
HOM6/null	Candidate gene	YPD	1.035	0.510×10^{-2}
HMO1/null	Candidate gene	YPD	0.994	0.846
PHO23/null	Candidate gene	YPD	1.036	0.118
RPL10/null	Candidate gene	YPD	0.942	1.48×10^{-2}
RPN10/null	Candidate gene	YPD	0.961	1.48×10^{-2}
BEM2/null	Candidate gene	F1 nitrogen-limited	0.987	0.462
ASC1/null	Candidate gene	F1 nitrogen-limited	1.008	0.445
RPL16A/null	Candidate gene	F1 nitrogen-limited	0.999	0.962
RPL8B/null	Candidate gene	F1 nitrogen-limited	0.995	0.445
UBI4/null	Candidate gene	F1 nitrogen-limited	0.996	0.682
RPL8A/null	Candidate gene	F1 nitrogen-limited	1.008	0.431
UBP3/null	Candidate gene	F1 nitrogen-limited	1.009	0.642
EFT2/null	Candidate gene	F1 nitrogen-limited	1.019	0.104
RPS6B/null	Candidate gene	F1 nitrogen-limited	0.965	1.35×10^{-4}
BCY1/null	Candidate gene	F1 nitrogen-limited	0.925	5.39×10^{-5}
BRE5/null	Candidate gene	F1 nitrogen-limited	0.956	1.00×10^{-3}
RPS19B/null	Candidate gene	F1 nitrogen-limited	0.991	0.404
RPL3/null	Candidate gene	F1 nitrogen-limited	0.973	0.158
ADH1/null	Candidate gene	F1 nitrogen-limited	0.993	0.445
GCN5/null	Candidate gene	F1 nitrogen-limited	0.982	0.445
ERG6/null	Candidate gene	F1 nitrogen-limited	1.003	0.720
SEC28/null	Candidate gene	F1 nitrogen-limited	0.948	3.33×10^{-4}
RPL28/null	Candidate gene	F1 nitrogen-limited	1.001	0.992
HOM6/null	Candidate gene	F1 nitrogen-limited	1.003	0.791
HMO1/null	Candidate gene	F1 nitrogen-limited	0.970	3.47×10^{-2}
PHO23/null	Candidate gene	F1 nitrogen-limited	0.986	0.158

RPL10/null	Candidate gene	F1 nitrogen-limited	0.986	0.104
RPN10/null	Candidate gene	F1 nitrogen-limited	0.994	0.431
BEM2/null	Candidate gene	F1 carbon-limited	0.972	0.544
ASC1/null	Candidate gene	F1 carbon-limited	0.972	0.242
RPL16A/null	Candidate gene	F1 carbon-limited	1.002	0.996
RPL8B/null	Candidate gene	F1 carbon-limited	1.007	0.976
UBI4/null	Candidate gene	F1 carbon-limited	1.015	0.841
RPL8A/null	Candidate gene	F1 carbon-limited	1.057	6.82×10^{-2}
UBP3/null	Candidate gene	F1 carbon-limited	1.003	0.976
EFT2/null	Candidate gene	F1 carbon-limited	1.027	0.544
RPS6B/null	Candidate gene	F1 carbon-limited	1.000	0.996
BCY1/null	Candidate gene	F1 carbon-limited	0.874	3.52×10^{-4}
BRE5/null	Candidate gene	F1 carbon-limited	0.963	0.107
RPS19B/null	Candidate gene	F1 carbon-limited	0.959	0.159
RPL3/null	Candidate gene	F1 carbon-limited	0.978	0.611
ADH1/null	Candidate gene	F1 carbon-limited	0.998	0.976
GCN5/null	Candidate gene	F1 carbon-limited	0.982	0.752
ERG6/null	Candidate gene	F1 carbon-limited	1.016	0.840
SEC28/null	Candidate gene	F1 carbon-limited	0.990	0.824
RPL28/null	Candidate gene	F1 carbon-limited	1.000	0.996
HOM6/null	Candidate gene	F1 carbon-limited	0.995	0.958
HMO1/null	Candidate gene	F1 carbon-limited	0.991	0.841
PHO23/null	Candidate gene	F1 carbon-limited	0.989	0.824
RPL10/null	Candidate gene	F1 carbon-limited	0.990	0.841
RPN10/null	Candidate gene	F1 carbon-limited	1.002	0.996
UPF3/null	Negative control	YPD	0.998	0.899
VAN1/null	Negative control	YPD	1.011	0.459
PUS1/null	Negative control	YPD	1.004	0.899
CFD1/null	Negative control	YPD	1.021	0.529
TRM82/null	Negative control	YPD	1.024	0.459
ATM1/null	Negative control	YPD	1.018	0.251
FET4/null	Negative control	YPD	1.035	5.33×10^{-2}
SLG1/null	Negative control	YPD	0.991	0.529
PRM10/null	Negative control	YPD	1.014	0.435
MPD2/null	Negative control	YPD	0.993	0.633
TMA64/null	Negative control	YPD	1.024	0.155
GPM3/null	Negative control	YPD	1.008	0.698
RBG2/null	Negative control	YPD	1.028	0.132
SEN54/null	Negative control	YPD	1.013	0.426
PAN6/null	Negative control	YPD	1.012	0.590
YEL1/null	Negative control	YPD	1.027	0.140
CNN1/null	Negative control	YPD	1.022	0.399
AIM36/null	Negative control	YPD	1.021	0.251
COS10/null	Negative control	YPD	0.990	0.719
ICS3/null	Negative control	YPD	1.002	0.952
PLB3/null	Negative control	YPD	1.000	0.972
BNA4/null	Negative control	YPD	0.991	0.633
FRE2/null	Negative control	YPD	1.004	0.719
CTS2/null	Negative control	YPD	1.009	0.633
SSD1/null	Background control	YPD	1.016	0.577
ZUO1/null	Background control	YPD	0.974	0.210
SFA1/null	Background control	YPD	1.011	0.595
CDC60/null	Background control	YPD	1.004	0.890
ATP11/null	Background control	YPD	1.023	0.324

RVB1/null	Background control	YPD	1.007	0.785
UBP15/null	Background control	YPD	0.995	0.770
ATP4/null	Background control	YPD	1.011	0.785
YAR1/null	Background control	YPD	0.997	0.886
YFH1/null	Background control	YPD	0.985	0.376
ARC40/null	Background control	YPD	1.028	0.210
MRP51/null	Background control	YPD	0.992	0.785
RSC9/null	Background control	YPD	0.969	0.157
APS3/null	Background control	YPD	0.987	0.605
REX4/null	Background control	YPD	1.010	0.715
KRE11/null	Background control	YPD	1.014	0.376
TAL1/null	Background control	YPD	0.996	0.881
IOC3/null	Background control	YPD	1.007	0.785
MIH1/null	Background control	YPD	1.009	0.657
CTA1/null	Background control	YPD	1.021	0.376
BUB1/null	Background control	YPD	0.871	2.35×10^{-5}
PCP1/null	Background control	YPD	0.999	0.940
YMD8/null	Background control	YPD	1.003	0.905
MRM2/null	Background control	YPD	1.016	0.553
ARK1/null	Background control	YPD	1.001	0.964
NSE3/null	Background control	YPD	1.000	0.964
NSG1/null	Background control	YPD	1.014	0.595
RGT1/null	Background control	YPD	0.996	0.863
VCX1/null	Background control	YPD	0.983	0.376
SMK1/null	Background control	YPD	0.979	0.376
AIM33/null	Background control	YPD	1.002	0.890
TVP38/null	Background control	YPD	0.996	0.858
ACF2/null	Background control	YPD	0.996	0.863
AAT1/null	Background control	YPD	1.020	0.577
ETP1/null	Background control	YPD	1.005	0.863
ALP1/null	Background control	YPD	1.027	6.24×10^{-2}
AIM32/null	Background control	YPD	1.007	0.657
HOR7/null	Background control	YPD	1.005	0.785
GAP1/null	Background control	YPD	1.002	0.905
PUG1/null	Background control	YPD	1.013	0.577
RTT105/null	Background control	YPD	1.028	0.210
THI20/null	Background control	YPD	0.971	0.210
RPL25/null	Positive control	YPD	0.810	1.84×10^{-5}
RPN11/null	Positive control	YPD	0.974	0.190
TUB1/null	Positive control	YPD	0.841	1.29×10^{-6}
